# Monitoring DNA Contamination in Handled vs. Directly Excavated Ancient Human Skeletal Remains

**DOI:** 10.1371/journal.pone.0052524

**Published:** 2013-01-25

**Authors:** Elena Pilli, Alessandra Modi, Ciro Serpico, Alessandro Achilli, Hovirag Lancioni, Barbara Lippi, Francesca Bertoldi, Sauro Gelichi, Martina Lari, David Caramelli

**Affiliations:** 1 Dipartimento di Biologia Evoluzionistica Laboratori di Antropologia, Università di Firenze, Firenze, Italy; 2 Dipartimento di Biologia Cellulare e Ambientale, Università di Perugia, Perugia, Italy; 3 Dipartimento di Studi Umanistici, Università Ca' Foscari Venezia, Venezia, Italy; Institut de Biologia Evolutiva - Universitat Pompeu Fabra, Spain

## Abstract

Bones, teeth and hair are often the only physical evidence of human or animal presence at an archaeological site; they are also the most widely used sources of samples for ancient DNA (aDNA) analysis. Unfortunately, the DNA extracted from ancient samples, already scarce and highly degraded, is widely susceptible to exogenous contaminations that can affect the reliability of aDNA studies. We evaluated the molecular effects of sample handling on five human skeletons freshly excavated from a cemetery dated between the 11 to the 14^th^ century. We collected specimens from several skeletal areas (teeth, ribs, femurs and ulnas) from each individual burial. We then divided the samples into two different sets: one labeled as “virgin samples” (i.e. samples that were taken by archaeologists under contamination-controlled conditions and then immediately sent to the laboratory for genetic analyses), and the second called “lab samples”(i.e. samples that were handled without any particular precautions and subject to normal washing, handling and measuring procedures in the osteological lab). Our results show that genetic profiles from “lab samples” are incomplete or ambiguous in the different skeletal areas while a different outcome is observed in the “virgin samples” set. Generally, all specimens from different skeletal areas in the exception of teeth present incongruent results between “lab” and “virgin” samples. Therefore teeth are less prone to contamination than the other skeletal areas we analyzed and may be considered a material of choice for classical aDNA studies. In addition, we showed that bones can also be a good candidate for human aDNA analysis if they come directly from the excavation site and are accompanied by a clear taphonomic history.

## Introduction

DNA analysis from ancient human remains is now a common practice even if contamination remains a serious problem. Modern human DNA is often found in highly degraded ancient DNA samples [1]–[8] but fortunately methods have been developed to recognize contaminant DNA [9]–[11]. Evidence suggests that one likely source of contamination is the direct handling and/or washing of the samples by the archaeologists and anthropologists. Their DNA may permeate through dentinal tubules into the pulp cavity of teeth and the Haversian system of bones [12]. It is less likely that DNA contaminants permeate as far as the osteocytes level [4], [5]. Other studies suggest that contamination most likely occurs during or immediately after excavation [12], [13]. The use of sterile gloves, masks and laboratory coats are fundamental precautions to avoid contamination especially since ancient human bones usually have only minute amounts of DNA which is often highly degraded. However, many current techniques used to decontaminate specimens - bleach, UV light, grinding or shot-blasting- reflect the mistaken belief that contamination is concentrated on the surfaces. Despite the importance of the contamination problem it has not yet been thoroughly studied. Indeed, studies of ancient human remains [14], even under the most stringent criteria for validating ancient human DNA sequences, did not provide conclusive proof that the sequences were authentic and without contamination [15]. Contamination can be ruled out only when the sequences are radically different from modern DNA, such as for Neandertals. Alternatively, it is possible to monitor contamination by the novel molecular approach of PEC (Primers Extension Capture) and ultra-deep sequencing methods (i.e. FLXing 454) [11].

In this study we investigated exogenous contamination, principally due to handling, on human skeletal remains freshly excavated from the cemetery of San Bartolomeo, Formigine (MO) in Italy. For each of the five individuals exhumed we collected samples from four skeletal areas: jaws with included teeth, ribs, femurs and ulnas. Samples were divided into “virgin samples” and “lab samples” based on the different handling approaches. All individuals that handled the samples were also typed. We focused our analysis on the HVS-I region of mitochondrial DNA which may help in discriminating contamination due its high variability. The aim of our study was to investigate: i. how handling samples without precautions can affect the final sequence results in term of mtDNA haplotype quality; ii. if the current standard techniques/precautions used in ancient DNA analysis are adequate for removing and/or identifying contamination from modern human mtDNA; iii. if some skeletal areas are more prone to contamination.

## Materials and Methods

### Ethics Statement

All necessary permits for the excavations and genetic analyses were obtained from Soprintendenza Archeologica per l'Emilia Romagna (Archaeological Authority for Emilia Romagna), Bologna, Italy.

### Ancient samples

We collected various remains of five different individuals derived from five different single burials (T148, T164, T170, T176, T189) excavated in the cemetery dated between the 11^th^ and 14th century near the church of San Bartolomeo, Formigine, in the province of Modena, Italy. For each individual we selected samples from four different skeletal areas (teeth, ribs, femurs and ulnas). Sampling followed protocols for the ancient DNA analysis of remains [16]. Those involved with the excavation wore sterile gloves, masks and special (or disposable) clothes. Specimens were immediately sent for analysis to the ancient DNA facility, where, in contamination-controlled conditions, small pieces were taken by using a drill with rotator blades from the bones and teeth were removed from the jaws. This set of samples was labelled as “virgin samples”. The second set of skeletal remains labelled “lab samples” were sent to the osteological laboratory where they were washed and studied by personnel who did not take any particular precautions on avoiding contaminations during handling. Following the osteological examinations, the remains were returned to the ancient DNA laboratory for genetic characterization using the same contamination-free protocols.

#### General equipment

Standard criteria for ancient samples were followed [17], [18]. All DNA extractions and PCR were carried out in a laboratory that was physically separated from the one where PCR cycling and post-PCR analysis was conducted. Disposable masks, gloves, and sterilized laboratory coats were worn throughout the process and were replaced regularly. The ceramic disposables used to pulverise the samples were rinsed after each use with 10% bleach, followed by ddH2O, and then UV-irradiated. Dedicated reagents and pipettes were utilized together with filter-plugged tips and were UV-irradiated after each use. All DNA extractions and PCR reactions included negative controls.

#### DNA extraction

To prevent contamination from prior handling, the outer layer of each bone was removed with a rotary tool, while the teeth were briefly soaked in 10% bleach. After the brushing and soaking procedure, each sample was irradiated for one hour under UV light, manually powdered in a mortar and extracted by means of a silica-based protocol [14, modified]. At least two independent extracts were obtained from each remain. A negative control was included for each extraction.

#### UNG treatment

In ancient DNA templates, uracil bases caused by hydrolytic deamination of cytosines, result in apparent C to T/G to A mutation in the final sequence results [2]. In order to excise these uracil residues and thereby minimize misincorporations due to this common post-mortem damage, 10 µl of DNA extracted from each sample was treated with 1 U of uracil-N-glycosylase (UNG) for 30 min at 37°C according to the manufacturer's instructions. After inactivation of the enzyme for 10 min at 94°C, the extracts were subjected to the same PCR, cloning and sequencing conditions as described above.

#### Quantification of DNA molecules

Real-time PCR amplification was performed using Brilliant1 SYBR1 Green QPCR Master Mix (Stratagene) in MX3000P (Stratagene), using 0.5 mM of appropriate primers (forward primer located at L16107 position and reverse primer located at H16261 position) following the same thermal cycling conditions reported in [19]. Ten-fold serial dilutions of the purified and quantified standard were included in the experiment to create the standard curve and determine the number of initial DNA molecules in the samples. For each individual of each sample set (“virgin samples” and “lab samples”) one extract from each skeletal area was quantified by Real-time amplification.

#### Amplification of mtDNA

Two microliters of extracted DNA were amplified for 50 cycles as described previously [19]. The 360 bp long HVS-I was subdivided into three overlapping fragments using the following primer pairs: L15995/H16132; L16107/H16261; L16247/H16402. Each extract was amplified at least twice with each primer set.

#### Cloning and sequencing

PCR products were cloned using TOPO TA Cloning Kit (Invitrogen) according to the manufacturer's instructions. Screening of white recombinant colonies was accomplished by PCR as reported in [19]. After purification with MinElute PCR purification Kit (Qiagen) a volume of 1.5 µl was cycle-sequenced following the BigDye Terminator kit (Applied Biosystems) supplier's instructions. The sequence was determined using an Applied BioSystems 3130 DNA Sequencer.

### Modern samples

In order to type all the people who handled the ancient samples, we collected oral swabs from the archaeologists, anthropologists and geneticists involved with this study. DNA extraction, as well as PCR and sequence reaction setup involving modern samples, was carried out in a laboratory that was physically separated from the laboratory where the ancient samples were analyzed. DNA was extracted using the Chelex® 100 (Bio-Rad Laboratories, Hercules, CA) [20] extraction protocol, followed by the amplification of two microliters by 28 cycles of PCR under the same conditions of the ancient samples. The entire mtDNA HVS-I region was amplified using a single primer pair named L15995-H16402. PCR products were purified with the MinElute PCR purification Kit (Qiagen) and then sequenced directly with the same amplification primer following the BigDye Terminator Kit supplier's instructions.

### Data analyses

A paired *t*-test was performed to calculate the significance of variation in quantitative PCR results between “virgin” and “lab” samples for each skeletal area. The t-test was run on Microsoft Office 2007 Excel with the suite MegaStat.

Each sample sequence derived from separate clones of different amplicons were aligned and compared against each other. Nucleotide substitutions observed in only one or few clones at a particular position were considered *Taq*-polymerase errors or cloning artifacts. Substitutions observed in the vast majority of clones were considered real mutations in the original template and reported in the final consensus sequences [2]. Finally the mutational differences relative to the revised Cambridge Reference Sequence (CRS) [21] were accurately analyzed in order to identify HVS-I mutational motifs for a possible haplogroup classification following the most updated human mitochondrial phylogeny [22].

## Results

### Quantitative PCR

Sporadic contamination and incorrect sequence determination is considered unlikely when the number of PCR template molecules (*target DNA*) is greater than 1,000 [2], [23]. Quantitative Real Time PCR results showed that sufficient amount of DNA for amplification was present in all the “virgin samples” except for rib T148 where quantification failed (table 1). We noted that for each burial site, teeth usually showed a greater number of starting DNA templates than other bones areas, but this feature was not observed in the “lab samples” set (table 1). When comparing results between the homologous “virgin” and “lab” samples, standard deviation values of starting template molecules in teeth were in the vast majority of comparison one to two orders of magnitude smaller than the same values in the other skeletal areas (table 1). We also noted that only the teeth presented a constant number of mtDNA molecules, while other fragments showed different patterns of template molecules. For example the T170 “lab” rib sample reported a low number of DNA molecules (780 mol/ul) when compared with the value from T170 “virgin” rib sample (1980 mol/ul). This results indicates that during the cleaning, DNA was probably washed out and that probably no exogenous DNA molecules were added by handling. On the contrary, sample T148 “lab” femur presented three times the number of mtDNA molecules of the corresponding T148 “virgin” femur; the increased DNA is probably due to exogenous molecules from handling as shown by the sequence motif results (see below).

**Table 1:** Results of quantitative Real-time PCR results.

		VIRGIN	LAB	MEAN	STD.DEV.
**148**	**TOOTH**	2900	2780	2840.0	8.49E+01
	**RIB**	0	7800	3900.0	5.52E+03
	**FEMUR**	1780	5200	3490.0	2.42E+03
	**ULNA**	1350	6510	3930.0	3.65E+03
**164**	**TOOTH**	3100	3200	3150.0	7.07E+01
	**RIB**	2580	6400	4490.0	2.70E+03
	**FEMUR**	2690	4890	3790.0	1.56E+03
	**ULNA**	2670	680	1675.0	1.41E+03
**170**	**TOOTH**	2480	2560	2520.0	5.66E+01
	**RIB**	1980	780	1380.0	8.49E+02
	**FEMUR**	2100	8540	5320.0	4.55E+03
	**ULNA**	1890	582	1236.0	9.25E+02
**176**	**TOOTH**	4250	4889	4569.5	4.52E+02
	**RIB**	2980	8520	5750.0	3.92E+03
	**FEMUR**	2450	7890	5170.0	3.85E+03
	**ULNA**	2650	9850	6250.0	5.09E+03
**189**	**TOOTH**	3700	3848	3774.0	1.05E+02
	**RIB**	2340	9540	5940.0	5.09E+03
	**FEMUR**	1980	8570	5275.0	4.66E+03
	**ULNA**	2040	896	1468.0	8.09E+02

Number of molecules per microliter (mol/µl) of the target DNA in the extracts are listed for each individual and each skeletal area in both “virgin” and “lab” sample sets. Mean values and standard deviations (mol/µl) calculated for each “virgin” sample and the corresponding “lab” sample are reported in the last two columns.

### HVS-I motif

We performed a double extraction for each skeletal area (tooth, rib, femur and ulna) of each sample set (“virgin” and “lab”) and each extract was amplified twice for each primer pair. Altogether, we sequenced at least 60 clones for each tooth or bone fragment (Datasets S1 and S2). Consensus sequences results for “virgin” and “lab” sample sets are described below and reported in table 2.

**Table 2:** Ancient samples HVS-I haplotypes between positions 16024 and 16384 and putative haplogroup classification for each burial and each skeletal area.

	T148		T164		T170		T176		T189	
	VIRGIN	LAB	VIRGIN	LAB	VIRGIN	LAB	VIRGIN	LAB	VIRGIN	LAB
**TOOTH**	16126	16126	CRS	CRS	CRS	CRS	16172	16172	CRS	CRS
**RIB**	N.A.	CRS	CRS	CRS	CRS	N.A.	CRS	CRS	CRS	CRS
**FEMUR**	16126	16126	CRS	16304	CRS	16093	16356	16304	CRS	CRS
**ULNA**	16126	CRS	CRS	N.A.	CRS	N.A.	16356	CRS	CRS	N.A.
**Putative Haplogroup(s)**	R0a or H	R0aH (16126)H (CRS)	H	H (CRS)H5 (16304)	H	H (CRS)H (16093)	H (16172)U4 (16356)	H (16172)H5 (16304)	H	H

In each haplotype, only positions that differ from CRS are listed. CRS means no differences.

### “Virgin samples” set

#### a. Same haplotypes among teeth and bones

T148, T164, T170 and T189. The misincorporation rate for samples T148, T164, T170 and T189 suggests that DNA templates were not highly damaged in all cases, with a large percentage of the clones (between 95% and 96.5%) showing the consensus nucleotide at each position (Dataset S1). No PCR results were obtained from the sample of rib T148. For each sample, the obtained haplotype was confirmed in all bones. All sample haplotypes were different from individuals who handled the sample (table 3).

**Table 3:** HVS-I haplotypes of modern samples between positions 16024 and 16384 and putative haplogroup classification.

ID	Haplotype	Putative Haplogroup
**Paleogeneticists**		
CS	16224, 16311	K
EP	16069, 16126, 16145, 16189, 16231, 16240, 16260, 16261	J1b
**Archeologists who recovered the samples**		
BL	16126, 16163, 16186, 16189, 16294	T1a
FB	16245, 16309	H
**Archeologists who handled the samples in the archeological laboratory**		
AS	CRS	H
BL	16126, 16163, 16186, 16189, 16294	T1a
FB	16245, 16309	H
MG	CRS	H
SL	CRS	H

See table 2 for further details.

#### b. Different haplotypes among teeth and bones

T176. The misincorporation rate suggests that the DNA templates were not highly damaged in teeth, with 95% of the clones showing the consensus nucleotide at each position while the other three bone elements presented higher rates of Taq misincorporations (between 85% and 90%, Dataset S1). This pattern was also reflected by the HVS-I motifs. As reported in table 2, tooth motif presented a single point mutation at np 16172, while the femur and the ulna samples matched the CRS control region motif and the rib showed a single mutation at 16356. In this latter case, all the motifs differed from the haplotypes of all the individuals who handled the sample both at the excavation and in the ancient DNA laboratory (table 3).

### “Lab sample” set

#### a. Different haplotypes among teeth and bones

T148, T164, T170 and T176. The misincorporation rate for samples T148, T164, T170 and T176 suggests that the starting DNA templates presented several differences between the four skeletal areas. While teeth always showed a misincorporation rate very similar to the corresponding “virgin” teeth samples (between 95% and 96.5%) and have the same haplotype, the other bone fragments presented different features (Dataset S2 and table 2). In the T148 sample rib, femur and ulna there wasn't any misincorporation. The femur showed a haplotype, with the 16126 transition, identical to both the T148 “virgin” samples and the “lab” tooth, while the ulna and rib samples displayed the CRS motif (table 2). Similarly, in the T164 samples, the rib and femur did not have any misincorporations while no PCR and sequence results were obtained from the ulna (N.A. in table 2). The haplotype obtained from the rib (CRS) is identical to the unique haplotype obtained from all the T164 “virgin” samples and from the tooth, while the femur presented a single mutation in position 16304 (table 2). In the T170 samples, no results were obtained from the rib and the ulna while no misincorporation was evident in the femur that presented an haplotype, with a single mutation in position 16093, different from that (CRS) obtained from all the T70 “virgin” samples and from the tooth (table 2). Finally, in the T176 samples all the three bone areas did not have any misincorporations and gave two different haplotypes: rib and ulna were CRS, and the femur presented a single substitution at position 16304. Both haplotypes are different from the single haplotype obtained from all the T176 “virgin” samples and from the tooth (single mutation at 16172, see table 2). Virtually, all the HVS-I motifs recovered from “lab” samples, except CRS, differed from the haplotypes of all the people who came in contact with the samples. It is worth noting that CRS was also the haplotype of three of the archaeologists who handled (but did not excavate) the remains (AS, MG and SL, see table 3).

#### b. Same haplotypes among teeth and bones

T189. The misincorporation rate suggests that the DNA templates present several differences between tooth and the other three bone fragments (rib, femur, and ulna); even in this case the tooth has the same misincorporation rate of the corresponding “virgin“ sample (i.e. 95%); the others 2 bone areas (rib and femur), on the contrary, did not present any misincorporation rates (Dataset S2). The same CRS haplotype was obtained from tooth, rib and femur while no results were obtained from the ulna (table 2). A CRS haplotype was also identified in all the T170 “virgin” samples and in three archaeologists (table 3).

## Discussion

This paper documents, for the first time, the effects on DNA contamination on human skeletal remains of handling by archaeologists and osteologists. Bones and teeth were followed from the excavation through to ancient DNA analysis. Unlike previous studies [9], [24], the samples came directly from excavation site and were specifically selected for examining these variables. We were able to directly monitor the effect of handling on the same sample set both during and after excavation. In addition we tested the effects on various skeletal areas analyzed collecting both teeth and different bone areas from the same and from different individuals. Other studies reported the intentional contamination of freshly excavated samples by exogenous source of DNA in the genetics lab just before ancient DNA analysis [13], [25]. In the present work samples were not intentionally ‘contaminated’ but were subjected to routine treatments (washing and measurement), thus better reflecting the actual conditions of the vast majority of archaeological samples subject to both osteological and genetic analyses.

Our results showed that teeth are less prone to contamination than the other skeletal areas. This result was inferred from both quantitative PCR and from reproducible sequence results comparing “virgin” and “lab” teeth for each individual (table 2). On the contrary, for the other skeletal areas quantitative PCR results in each individual showed a wide variation between the two samples sets (table 1). This variation was statistically significant in ribs [P (*t* test paired) = 0.046] and femurs [P (*t* test paired) = 0.005], but not in ulnas and teeth.

The reproducibility of sequencing results obtained from the three bone fragments (rib, femur and ulna) showed different patterns in the different burial sites (table 2). For example, in the T148 “lab” samples only the femur had the same haplotype observed in all the “virgin” samples and in the “lab” tooth from the same burial. A similar pattern was seen in the T164 “lab” samples in which only the rib showed the same haplotype observed in all the “virgin” samples and in the “lab” tooth. Among the T170 “lab” samples only the tooth provided a reproducible sequence compared to the results obtained from the “virgin” samples set. Burial T176 is a particular case. Here the “virgin” samples set presented very biased results probably due to the high DNA degradation of the sample as suggested also by the high level of nucleotide misincorporations. The same haplotypes for all the skeletal fragments in both “virgin” and “lab” sample sets were obtained only for the T189 burial site.

If we compare the HVS-I sequence data of both sample sets with the quantitative PCR results, we can observe a proportional increase in the number of mtDNA molecules in all “lab” samples in which appear the CRS motif (see table 1, T148 rib and ulna, T164 rib, T176 rib and ulna and T189 rib and femur); this is probably due to exogenous DNA contributions by the anthropologist who handled the samples in the osteological laboratory (but not during excavation) considering that three out of five of them have the CRS motif. Exogenous contaminations from modern DNA can be further supported by misincorporation rate that, in these cases, is zero (Dataset S1).

The different results from teeth and bones reported in this study can be explained by the fact that tooth enamel is impermeable to water [26] and the root is located in the alveolus making it more difficult for contaminants to penetrate the inner core of the teeth [12], [13]. An alternative explanation may be that different methods of surface decontamination for teeth and bones can influence the results. Due to the different morphology of the samples selected for this study (intact teeth and bone fragments), we used slightly different decontamination methods: the outer layer of each bone fragment was initially removed with a rotary tool, while teeth were briefly soaked in 10% bleach, then all the samples were UV irradiated. Bleach was not used to decontaminate bone surfaces because, due to fragmentation and degrees of compactness, it can differentially penetrate into bone fragments and influence the final results. On the other hand, bleach was preferred to decontaminate intact teeth mainly because not all surfaces are accessible to the rotary tool. It is unlikely that these different methods influenced our results because both decontamination methods provided in “virgin samples” the same results for all bone areas of the same individual (table 2). Moreover, previous studies showed that bleach treatment more effectively reduces contamination if bone powder, and not intact or fragmented bones, is soaked in bleach just before DNA extraction [27], [28]. Unfortunately, the amount of authentic ancient DNA also decreased in the samples when bone powder was treated with bleach [27]. For these reasons, the treatments performed in this study were the best choices to effectively decontaminate sample surfaces from exogenous DNA by avoiding any differential reduction of endogenous ancient molecules.

Our results clearly indicate that practices most frequently followed to sterilize ancient samples before ancient DNA analysis, by removing the outside layer by drilling and/or with bleach treatment and subsequent UV irradiation, work well only for teeth still located in the alveolus and for bone samples freshly excavated and not extensively handled. If bones were subjected to handling, as during a typical osteological study, the sterilization procedures, which followed in the ancient DNA lab were not effective. Apparently, exogenous contaminations had penetrated the internal part of the bones most likely due to washing. More critically, we showed that standard ancient DNA procedures, consisting of multiple extractions and PCRs, cloning and sequencing of multiple clones if multiple extractions derive only from one skeletal area were not sufficient to detect contamination. Consistent sequence results in fact were obtained from the double extractions and amplifications derived from each bone fragments in the handled “lab” samples. Importantly, incongruences between the motifs were highlighted comparing results from different bone areas and it was not possible to determine with certainty which haplotype is endogenous.

## Conclusion

Our study demonstrated that teeth should be the materials of choice in classical ancient DNA studies because they are more refractory to contamination by exogenous DNA than bones. In addition, we showed that bones can also be good candidates for molecular analyses, if they come directly from the excavation site and have a known taphonomic history. A complete record must be kept of all individuals who handled the remains during and after the excavation. Alternatively, when dealing with samples handled without precautions, it is highly recommended to adopt new approaches able to discriminate between endogenous and contaminant DNA [11].

## Supporting Information

Dataset S1 DNA sequences of clones analysed respectively in “Virgin samples” set and “Lab samples” set. The first lines report the Cambridge Reference Sequence (CRS) with the numbering of the nucleotide positions. In the clones sequences nucleotides identical to CRS are indicated by dots. Clones are identified by sample name and 3 digits indicating respectively the number of extraction, the number of PCR and the number of clone. Also the skeletal district from which each sequence derives is reported.
(DOC)

Dataset S2 DNA sequences of clones analysed respectively in “Virgin samples” set and “Lab samples” set. The first lines report the Cambridge Reference Sequence (CRS) with the numbering of the nucleotide positions. In the clones sequences nucleotides identical to CRS are indicated by dots. Clones are identified by sample name and 3 digits indicating respectively the number of extraction, the number of PCR and the number of clone. Also the.
(DOC)
