# Supplementary material for: Monitoring DNA Contamination in Handled vs. Directly Excavated Ancient Human Skeletal Remains
Source: PLoS One. 2013 Jan 25;8(1):e52524. doi: 10.1371/journal.pone.0052524 (PMC3556025; doi:10.1371/journal.pone.0052524)
Supplement: Dataset S1 — DNA sequences of clones analysed respectively in “Virgin samples” set and “Lab samples” set. The first lines report the Cambridge Reference Sequence (CRS) with the numbering of the nucleotide positions. In the clones sequences nucleotides identical to CRS are indicated by dots. Clones are identified by sample name and 3 digits indicating respectively the number of extraction, the number of PCR and the number of clone. Also the skeletal district from which each sequence derives is reported. (DOC) [file pone.0052524.s001.doc]

"Virgin samples" set

1111111111111111111111111111111111111111111111111111111111111111111111111111111111111111111111111111111111111111111111111111111111111111111111111111111111111111111111111111111111111111111111111111111111111111111111111111111111111111111111111111111111111111111111111111111111111111111111111111111111111111111111111111111111111111111111111111111111111111111111111

6666666666666666666666666666666666666666666666666666666666666666666666666666666666666666666666666666666666666666666666666666666666666666666666666666666666666666666666666666666666666666666666666666666666666666666666666666666666666666666666666666666666666666666666666666666666666666666666666666666666666666666666666666666666666666666666666666666666666666666666666

0000000000000000000000000000000000000000000000000000000000000000000000000000111111111111111111111111111111111111111111111111111111111111111111111111111111111111111111111111111122222222222222222222222222222222222222222222222222222222222222222222222222222222222222222222222222223333333333333333333333333333333333333333333333333333333333333333333333333333333333333

2222223333333333444444444455555555556666666666777777777788888888889999999999000000000011111111112222222222333333333344444444445555555555666666666677777777778888888888999999999900000000001111111111222222222233333333334444444444555555555566666666667777777777888888888899999999990000000000111111111122222222223333333333444444444455555555556666666666777777777788888

4567890123456789012345678901234567890123456789012345678901234567890123456789012345678901234567890123456789012345678901234567890123456789012345678901234567890123456789012345678901234567890123456789012345678901234567890123456789012345678901234567890123456789012345678901234567890123456789012345678901234567890123456789012345678901234567890123456789012345678901234

CRS TTCTTTCATGGGGAAGCAGATTTGGGTACCACCCAAGTATTGACTCACCCATCAACAACCGCTATGTATTTCGTACATTACTGCCAGCCACCATGAATATTGTACGGTACCATAAATACTTGACCACCTGTAGTACATAAAAACCCAATCCACATCAAAACCCCCTCCCCATGCTTACAAGCAAGTACAGCAATCAACCCTCAACTATCACACATCAACTGCAACTCCAAAGCCACCCCTCACCCACTAGGATACCAACAAACCTACCCACCCTTAACAGTACATAGTACATAAAGCCATTTACCGTACATAGCACATTACAGTCAAATCCCTTCTCGTCCCCATGGATGACCCCCCTCAG

>T148

T148.1.1.1_tooth ......................................................................................................C.....ACCATAAATACTTGACCACCTGTAGTAC

T148.1.1.2_tooth ......................................................................................................C.....

T148.1.1.3_tooth ...........................................T..........................................................C.....

T148.1.1.4_tooth ......................................................................................................C.....

T148.1.1.5_tooth ......................................................................................................C.....

T148.1.2.1_tooth ......................................................................................................C.....

T148.1.2.2_tooth ......................................................................................................C.....

T148.1.2.3_tooth ......................................................................................................C.....

T148.1.2.4_tooth ......................................................................................................C.....

T148.1.2.5_tooth ......................................................................................................C.....

T148.2.1.1_tooth ......................................................................................................C.....

T148.2.1.2_tooth ......................................................................................................C.....

T148.2.1.3_tooth ......................................................................................................C.....

T148.2.1.4_tooth ......................................................................................................C.....

T148.2.1.5_tooth ......................................................................................................C.....

T148.2.2.1_tooth ......................................................................................................C.....

T148.2.2.2_tooth ......................................................................................................C.....

T148.2.2.3_tooth ......................................................................................................C.....

T148.2.2.4_tooth ......................................................................................................C.....

T148.2.2.5_tooth ......................................................................................................C.....

T148.1.1.1_tooth CGCTATGTATTTCGTACATTACTGC..................C......................................................................................................................................CCTCACCCACTAGGATACCA

T148.1.1.2_tooth ..................C......................................................................................................................................

T148.1.1.3_tooth ..................C......................................................................................................................................

T148.1.1.4_tooth ..................C......................................................................................................................................

T148.1.1.5_tooth ..................C......................................................................................................................................

T148.1.2.1_tooth ..................C......................................................................................................................................

T148.1.2.2_tooth ..................C......................................................................................................................................

T148.1.2.3_tooth ..................C......................................................................................................................................

T148.1.2.4_tooth ..................C......................................................................................................................................

T148.1.2.5_tooth ..................C......................................................................................................................................

T148.2.1.1_tooth ..................C......................................................................................................................................

T148.2.1.2_tooth ..................C......................................................................................................................................

T148.2.1.3_tooth ..................C......................................................................................................................................

T148.2.1.4_tooth ..................C......................................................................................................................................

T148.2.1.5_tooth ..................C......................................................................................................................................

T148.2.2.1_tooth ..................C......................................................................................................................................

T148.2.2.2_tooth ..................C...................................................................................................................T..................

T148.2.2.3_tooth ..................C......................................................................................................................................

T148.2.2.4_tooth ..................C......................................................................................................................................

T148.2.2.5_tooth ..................C......................................................................................................................................

T148.1.1.1_tooth CAACTATCACACATCAACTGCAA.........................................................................................................................................

T148.1.1.2_tooth .........................................................................................................................................

T148.1.1.3_tooth .........................................................................................................................................

T148.1.1.4_tooth .........................................................................................................................................

T148.1.1.5_tooth .........................................................................................................................................

T148.1.2.1_tooth .........................................................................................................................................

T148.1.2.2_tooth .........................................................................................................................................

T148.1.2.3_tooth .........................................................................................................................................

T148.1.2.4_tooth .........................................................................................................................................

T148.1.2.5_tooth .........................................................................................................................................

T148.2.1.1_tooth .........................................................................................................................................

T148.2.1.2_tooth .........................................................................................................................................

T148.2.1.3_tooth .........................................................................................................................................

T148.2.1.4_tooth .........................................................................................................................................

T148.2.1.5_tooth .........................................................................................................................................

T148.2.2.1_tooth .........................................................................................................................................

T148.2.2.2_tooth .........................................................................................................................................

T148.2.2.3_tooth .........................................................................................................................................

T148.2.2.4_tooth .........................................................................................................................................

T148.2.2.5_tooth .........................................................................................................................................

consensus TTCTTTCATGGGGAAGCAGATTTGGGTACCACCCAAGTATTGACTCACCCATCAACAACCGCTATGTATTTCGTACATTACTGCCAGCCACCATGAATATTGCACGGTACCATAAATACTTGACCACCTGTAGTACATAAAAACCCAATCCACATCAAAACCCCCTCCCCATGCTTACAAGCAAGTACAGCAATCAACCCTCAACTATCACACATCAACTGCAACTCCAAAGCCACCCCTCACCCACTAGGATACCAACAAACCTACCCACCCTTAACAGTACATAGTACATAAAGCCATTTACCGTACATAGCACATTACAGTCAAATCCCTTCTCGTCCCCATGGATGACCCCCCTCAG

1111111111111111111111111111111111111111111111111111111111111111111111111111111111111111111111111111111111111111111111111111111111111111111111111111111111111111111111111111111111111111111111111111111111111111111111111111111111111111111111111111111111111111111111111111111111111111111111111111111111111111111111111111111111111111111111111111111111111111111111111

6666666666666666666666666666666666666666666666666666666666666666666666666666666666666666666666666666666666666666666666666666666666666666666666666666666666666666666666666666666666666666666666666666666666666666666666666666666666666666666666666666666666666666666666666666666666666666666666666666666666666666666666666666666666666666666666666666666666666666666666666

0000000000000000000000000000000000000000000000000000000000000000000000000000111111111111111111111111111111111111111111111111111111111111111111111111111111111111111111111111111122222222222222222222222222222222222222222222222222222222222222222222222222222222222222222222222222223333333333333333333333333333333333333333333333333333333333333333333333333333333333333

2222223333333333444444444455555555556666666666777777777788888888889999999999000000000011111111112222222222333333333344444444445555555555666666666677777777778888888888999999999900000000001111111111222222222233333333334444444444555555555566666666667777777777888888888899999999990000000000111111111122222222223333333333444444444455555555556666666666777777777788888

4567890123456789012345678901234567890123456789012345678901234567890123456789012345678901234567890123456789012345678901234567890123456789012345678901234567890123456789012345678901234567890123456789012345678901234567890123456789012345678901234567890123456789012345678901234567890123456789012345678901234567890123456789012345678901234567890123456789012345678901234

CRS TTCTTTCATGGGGAAGCAGATTTGGGTACCACCCAAGTATTGACTCACCCATCAACAACCGCTATGTATTTCGTACATTACTGCCAGCCACCATGAATATTGTACGGTACCATAAATACTTGACCACCTGTAGTACATAAAAACCCAATCCACATCAAAACCCCCTCCCCATGCTTACAAGCAAGTACAGCAATCAACCCTCAACTATCACACATCAACTGCAACTCCAAAGCCACCCCTCACCCACTAGGATACCAACAAACCTACCCACCCTTAACAGTACATAGTACATAAAGCCATTTACCGTACATAGCACATTACAGTCAAATCCCTTCTCGTCCCCATGGATGACCCCCCTCAG

>T148

T148.1.1.1_femur ......................................................................................................C.....ACCATAAATACTTGACCACCTGTAGTAC

T148.1.1.2_femur ......................................................................................................C.....

T148.1.1.3_femur ......................................................................................................C.....

T148.1.1.4_femur ......................................................................................................C.....

T148.1.1.5_femur ......................................................................................................C.....

T148.1.2.1_femur ......................................................................................................C.....

T148.1.2.2_femur ......................................................................................................C.....

T148.1.2.3_femur ......................................................................................................C.....

T148.1.2.4_femur ......................................................................................................C.....

T148.1.2.5_femur ......................................................................................................C.....

T148.2.1.1_femur ......................................................................................................C.....

T148.2.1.2_femur ......................................................................................................C.....

T148.2.1.3_femur ................T.....................................................................................C.....

T148.2.1.4_femur ......................................................................................................C.....

T148.2.1.5_femur ......................................................................................................C.....

T148.2.2.1_femur ......................................................................................................C.....

T148.2.2.2_femur ......................................................................................................C.....

T148.2.2.3_femur ......................................................................................................C.....

T148.2.2.4_femur ......................................................................................................C.....

T148.2.2.5_femur ......................................................................................................C.....

T148.1.1.1_femur CGCTATGTATTTCGTACATTACTGC..................C......................................................................................................................................CCTCACCCACTAGGATACCA

T148.1.1.2_femur ..................C......................................................................................................................................

T148.1.1.3_femur ..................C......................................................................................................................................

T148.1.1.4_femur ..................C......................................................................................................................................

T148.1.1.5_femur ..................C......................................................................................................................................

T148.1.2.1_femur ..................C......................................................................................................................................

T148.1.2.2_femur ..................C......................................................................................................................................

T148.1.2.3_femur ..................C......................................................................................................................................

T148.1.2.4_femur ..................C......................................................................................................................................

T148.1.2.5_femur ..................C......................................................................................................................................

T148.2.1.1_femur ..................C......................................................................................................................................

T148.2.1.2_femur ..................C......................................................................................................................................

T148.2.1.3_femur ..................C......................................................................................................................................

T148.2.1.4_femur ..................C.....................................................................................................T................................

T148.2.1.5_femur ..................C......................................................................................................................................

T148.2.2.1_femur ..................C......................................................................................................................................

T148.2.2.2_femur ..................C......................................................................................................................................

T148.2.2.3_femur ..................C......................................................................................................................................

T148.2.2.4_femur ..................C...................................................................................................................T..................

T148.2.2.5_femur ..................C......................................................................................................................................

T148.1.1.1_femur CAACTATCACACATCAACTGCAA.........................................................................................................................................

T148.1.1.2_femur .........................................................................................................................................

T148.1.1.3_femur .........................................................................................................................................

T148.1.1.4_femur .........................................................................................................................................

T148.1.1.5_femur .........................................................................................................................................

T148.1.2.1_femur .........................................................................................................................................

T148.1.2.2_femur .........................................................................................................................................

T148.1.2.3_femur .........................................................................................................................................

T148.1.2.4_femur .........................................................................................................................................

T148.1.2.5_femur .........................................................................................................................................

T148.2.1.1_femur .........................................................................................................................................

T148.2.1.2_femur .........................................................................................................................................

T148.2.1.3_femur .........................................................................................................................................

T148.2.1.4_femur .........................................................................................................................................

T148.2.1.5_femur .........................................................................................................................................

T148.2.2.1_femur .........................................................................................................................................

T148.2.2.2_femur .........................................................................................................................................

T148.2.2.3_femur .........................................................................................................................................

T148.2.2.4_femur .........................................................................................................................................

T148.2.2.5_femur .........................................................................................................................................

consensus TTCTTTCATGGGGAAGCAGATTTGGGTACCACCCAAGTATTGACTCACCCATCAACAACCGCTATGTATTTCGTACATTACTGCCAGCCACCATGAATATTGCACGGTACCATAAATACTTGACCACCTGTAGTACATAAAAACCCAATCCACATCAAAACCCCCTCCCCATGCTTACAAGCAAGTACAGCAATCAACCCTCAACTATCACACATCAACTGCAACTCCAAAGCCACCCCTCACCCACTAGGATACCAACAAACCTACCCACCCTTAACAGTACATAGTACATAAAGCCATTTACCGTACATAGCACATTACAGTCAAATCCCTTCTCGTCCCCATGGATGACCCCCCTCAG

1111111111111111111111111111111111111111111111111111111111111111111111111111111111111111111111111111111111111111111111111111111111111111111111111111111111111111111111111111111111111111111111111111111111111111111111111111111111111111111111111111111111111111111111111111111111111111111111111111111111111111111111111111111111111111111111111111111111111111111111111

6666666666666666666666666666666666666666666666666666666666666666666666666666666666666666666666666666666666666666666666666666666666666666666666666666666666666666666666666666666666666666666666666666666666666666666666666666666666666666666666666666666666666666666666666666666666666666666666666666666666666666666666666666666666666666666666666666666666666666666666666

0000000000000000000000000000000000000000000000000000000000000000000000000000111111111111111111111111111111111111111111111111111111111111111111111111111111111111111111111111111122222222222222222222222222222222222222222222222222222222222222222222222222222222222222222222222222223333333333333333333333333333333333333333333333333333333333333333333333333333333333333

2222223333333333444444444455555555556666666666777777777788888888889999999999000000000011111111112222222222333333333344444444445555555555666666666677777777778888888888999999999900000000001111111111222222222233333333334444444444555555555566666666667777777777888888888899999999990000000000111111111122222222223333333333444444444455555555556666666666777777777788888

4567890123456789012345678901234567890123456789012345678901234567890123456789012345678901234567890123456789012345678901234567890123456789012345678901234567890123456789012345678901234567890123456789012345678901234567890123456789012345678901234567890123456789012345678901234567890123456789012345678901234567890123456789012345678901234567890123456789012345678901234

CRS TTCTTTCATGGGGAAGCAGATTTGGGTACCACCCAAGTATTGACTCACCCATCAACAACCGCTATGTATTTCGTACATTACTGCCAGCCACCATGAATATTGTACGGTACCATAAATACTTGACCACCTGTAGTACATAAAAACCCAATCCACATCAAAACCCCCTCCCCATGCTTACAAGCAAGTACAGCAATCAACCCTCAACTATCACACATCAACTGCAACTCCAAAGCCACCCCTCACCCACTAGGATACCAACAAACCTACCCACCCTTAACAGTACATAGTACATAAAGCCATTTACCGTACATAGCACATTACAGTCAAATCCCTTCTCGTCCCCATGGATGACCCCCCTCAG

>T148

T148.1.1.1_ulna ......................................................................................................C.....ACCATAAATACTTGACCACCTGTAGTAC

T148.1.1.2_ulna ......................................................................................................C.....

T148.1.1.3_ulna ......................................................................................................C.....

T148.1.1.4_ulna ......................................................................................................C.....

T148.1.1.5_ulna ......................................................................................................C.....

T148.1.2.1_ulna ......................................................................................................C.....

T148.1.2.2_ulna ......................................................................................................C.....

T148.1.2.3_ulna ......................................................................................................C.....

T148.1.2.4_ulna ......................................................................................................C.....

T148.1.2.5_ulna ......................................................................................................C.....

T148.2.1.1_ulna ......................................................................................................C.....

T148.2.1.2_ulna ......................................................................................................C.....

T148.2.1.3_ulna ......................................................................................................C.....

T148.2.1.4_ulna ......................................................................................................C.....

T148.2.1.5_ulna ......................................................................................................C.....

T148.2.2.1_ulna ......................................................................................................C.....

T148.2.2.2_ulna ......................................................................................................C.....

T148.2.2.3_ulna ......................................................................................................C.....

T148.2.2.4_ulna ......................................................................................................C.....

T148.2.2.5_ulna ......................................................................................................C.....

T148.1.1.1_ulna CGCTATGTATTTCGTACATTACTGC..................C......................................................................................................................................CCTCACCCACTAGGATACCA

T148.1.1.2_ulna ..................C......................................................................................................................................

T148.1.1.3_ulna ..................C......................................................................................................................................

T148.1.1.4_ulna ..................C......................................................................................................................................

T148.1.1.5_ulna ..................C......................................................................................................................................

T148.1.2.1_ulna ..................C......................................................................................................................................

T148.1.2.2_ulna ..................C......................................................................................................................................

T148.1.2.3_ulna .......T..........C......................................................................................................................................

T148.1.2.4_ulna ..................C......................................................................................................................................

T148.1.2.5_ulna ..................C......................................................................................................................................

T148.2.1.1_ulna ..................C......................................................................................................................................

T148.2.1.2_ulna ..................C......................................................................................................................................

T148.2.1.3_ulna ..................C......................................................................................................................................

T148.2.1.4_ulna ..................C......................................................................................................................................

T148.2.1.5_ulna ..................C......................................................................................................................................

T148.2.2.1_ulna ..................C......................................................................................................................................

T148.2.2.2_ulna ..................C......................................................................................................................................

T148.2.2.3_ulna ..................C......................................................................................................................................

T148.2.2.4_ulna ..................C......................................................................................................................................

T148.2.2.5_ulna ..................C......................................................................................................................................

T148.1.1.1_ulna CAACTATCACACATCAACTGCAA.........................................................................................................................................

T148.1.1.2_ulna .........................................................................................................................................

T148.1.1.3_ulna .........................................................................................................................................

T148.1.1.4_ulna .........................................................................................................................................

T148.1.1.5_ulna .........................................................................................................................................

T148.1.2.1_ulna .........................................................................................................................................

T148.1.2.2_ulna .........................................................................................................................................

T148.1.2.3_ulna .........................................................................................................................................

T148.1.2.4_ulna .........................................................................................................................................

T148.1.2.5_ulna .........................................................................................................................................

T148.2.1.1_ulna ...........................................................................................T.............................................

T148.2.1.2_ulna .........................................................................................................................................

T148.2.1.3_ulna .........................................................................................................................................

T148.2.1.4_ulna .........................................................................................................................................

T148.2.1.5_ulna .........................................................................................................................................

T148.2.2.1_ulna .........................................................................................................................................

T148.2.2.2_ulna .........................................................................................................................................

T148.2.2.3_ulna .........................................................................................................................................

T148.2.2.4_ulna .........................................................................................................................................

T148.2.2.5_ulna .........................................................................................................................................

consensus TTCTTTCATGGGGAAGCAGATTTGGGTACCACCCAAGTATTGACTCACCCATCAACAACCGCTATGTATTTCGTACATTACTGCCAGCCACCATGAATATTGCACGGTACCATAAATACTTGACCACCTGTAGTACATAAAAACCCAATCCACATCAAAACCCCCTCCCCATGCTTACAAGCAAGTACAGCAATCAACCCTCAACTATCACACATCAACTGCAACTCCAAAGCCACCCCTCACCCACTAGGATACCAACAAACCTACCCACCCTTAACAGTACATAGTACATAAAGCCATTTACCGTACATAGCACATTACAGTCAAATCCCTTCTCGTCCCCATGGATGACCCCCCTCAG

1111111111111111111111111111111111111111111111111111111111111111111111111111111111111111111111111111111111111111111111111111111111111111111111111111111111111111111111111111111111111111111111111111111111111111111111111111111111111111111111111111111111111111111111111111111111111111111111111111111111111111111111111111111111111111111111111111111111111111111111111

6666666666666666666666666666666666666666666666666666666666666666666666666666666666666666666666666666666666666666666666666666666666666666666666666666666666666666666666666666666666666666666666666666666666666666666666666666666666666666666666666666666666666666666666666666666666666666666666666666666666666666666666666666666666666666666666666666666666666666666666666

0000000000000000000000000000000000000000000000000000000000000000000000000000111111111111111111111111111111111111111111111111111111111111111111111111111111111111111111111111111122222222222222222222222222222222222222222222222222222222222222222222222222222222222222222222222222223333333333333333333333333333333333333333333333333333333333333333333333333333333333333

2222223333333333444444444455555555556666666666777777777788888888889999999999000000000011111111112222222222333333333344444444445555555555666666666677777777778888888888999999999900000000001111111111222222222233333333334444444444555555555566666666667777777777888888888899999999990000000000111111111122222222223333333333444444444455555555556666666666777777777788888

4567890123456789012345678901234567890123456789012345678901234567890123456789012345678901234567890123456789012345678901234567890123456789012345678901234567890123456789012345678901234567890123456789012345678901234567890123456789012345678901234567890123456789012345678901234567890123456789012345678901234567890123456789012345678901234567890123456789012345678901234

CRS TTCTTTCATGGGGAAGCAGATTTGGGTACCACCCAAGTATTGACTCACCCATCAACAACCGCTATGTATTTCGTACATTACTGCCAGCCACCATGAATATTGTACGGTACCATAAATACTTGACCACCTGTAGTACATAAAAACCCAATCCACATCAAAACCCCCTCCCCATGCTTACAAGCAAGTACAGCAATCAACCCTCAACTATCACACATCAACTGCAACTCCAAAGCCACCCCTCACCCACTAGGATACCAACAAACCTACCCACCCTTAACAGTACATAGTACATAAAGCCATTTACCGTACATAGCACATTACAGTCAAATCCCTTCTCGTCCCCATGGATGACCCCCCTCAG

>T164

T164.1.1.1_tooth ............................................................................................................ACCATAAATACTTGACCACCTGTAGTAC

T164.1.1.2_tooth ............................................................................................................

T164.1.1.3_tooth ............................................................................................................

T164.1.1.4_tooth ............................................................................................................

T164.1.1.5_tooth ............................................................................................................

T164.1.2.1_tooth ............................................................................................................

T164.1.2.2_tooth ............................................................................................................

T164.1.2.3_tooth ............................................................................................................

T164.1.2.4_tooth ............................................................................................................

T164.1.2.5_tooth ............................................................................................................

T164.2.1.1_tooth ............................................................................................................

T164.2.1.2_tooth ............................................................................................................

T164.2.1.3_tooth ............................................................................................................

T164.2.1.4_tooth ................T...........................................................................................

T164.2.1.5_tooth ............................................................................................................

T164.2.2.1_tooth ............................................................................................................

T164.2.2.2_tooth ............................................................................................................

T164.2.2.3_tooth ............................................................................................................

T164.2.2.4_tooth ............................................................................................................

T164.2.2.5_tooth ............................................................................................................

T164.1.1.1_tooth CGCTATGTATTTCGTACATTACTGC.........................................................................................................................................................CCTCACCCACTAGGATACCA

T164.1.1.2_tooth .........................................................................................................................................................

T164.1.1.3_tooth .........................................................................................................................................................

T164.1.1.4_tooth .........................................................................................................................................................

T164.1.1.5_tooth .........................................................................................................................................................

T164.1.2.1_tooth .........................................................................................................................................................

T164.1.2.2_tooth .........................................................................................................................................................

T164.1.2.3_tooth .........................................................................................................................................................

T164.1.2.4_tooth .........................................................................................................................................................

T164.1.2.5_tooth .........................................................................................................................................................

T164.2.1.1_tooth .........................................................................................................................................................

T164.2.1.2_tooth .........................................................................................................................................................

T164.2.1.3_tooth .........................................................................................................................................................

T164.2.1.4_tooth .........................................................................................................................................................

T164.2.1.5_tooth .........................................................................................................................................................

T164.2.2.1_tooth .........................................................................................................................................................

T164.2.2.2_tooth .........................................................................................................................................................

T164.2.2.3_tooth .........................................................................................................................................................

T164.2.2.4_tooth .........................................................................................................................................................

T164.2.2.5_tooth .........................................................................................................................................................

T164.1.1.1_tooth CAACTATCACACATCAACTGCAA.........................................................................................................................................

T164.1.1.2_tooth .........................................................................................................................................

T164.1.1.3_tooth ........T................................................................................................................................

T164.1.1.4_tooth .........................................................................................................................................

T164.1.1.5_tooth .........................................................................................................................................

T164.1.2.1_tooth .........................................................................................................................................

T164.1.2.2_tooth .........................................................................................................................................

T164.1.2.3_tooth .........................................................................................................................................

T164.1.2.4_tooth .........................................................................................................................................

T164.1.2.5_tooth .........................................................................................................................................

T164.2.1.1_tooth .........................................................................................................................................

T164.2.1.2_tooth .........................................................................................................................................

T164.2.1.3_tooth .........................................................................................................................................

T164.2.1.4_tooth .........................................................................................................................................

T164.2.1.5_tooth .........................................................................................................................................

T164.2.2.1_tooth .........................................................................................................................................

T164.2.2.2_tooth .........................................................................................................................................

T164.2.2.3_tooth ................................................................................................................A........................

T164.2.2.4_tooth .........................................................................................................................................

T164.2.2.5_tooth .........................................................................................................................................

Consensus TTCTTTCATGGGGAAGCAGATTTGGGTACCACCCAAGTATTGACTCACCCATCAACAACCGCTATGTATTTCGTACATTACTGCCAGCCACCATGAATATTGTACGGTACCATAAATACTTGACCACCTGTAGTACATAAAAACCCAATCCACATCAAAACCCCCTCCCCATGCTTACAAGCAAGTACAGCAATCAACCCTCAACTATCACACATCAACTGCAACTCCAAAGCCACCCCTCACCCACTAGGATACCAACAAACCTACCCACCCTTAACAGTACATAGTACATAAAGCCATTTACCGTACATAGCACATTACAGTCAAATCCCTTCTCGTCCCCATGGATGACCCCCCTCAG

1111111111111111111111111111111111111111111111111111111111111111111111111111111111111111111111111111111111111111111111111111111111111111111111111111111111111111111111111111111111111111111111111111111111111111111111111111111111111111111111111111111111111111111111111111111111111111111111111111111111111111111111111111111111111111111111111111111111111111111111111

6666666666666666666666666666666666666666666666666666666666666666666666666666666666666666666666666666666666666666666666666666666666666666666666666666666666666666666666666666666666666666666666666666666666666666666666666666666666666666666666666666666666666666666666666666666666666666666666666666666666666666666666666666666666666666666666666666666666666666666666666

0000000000000000000000000000000000000000000000000000000000000000000000000000111111111111111111111111111111111111111111111111111111111111111111111111111111111111111111111111111122222222222222222222222222222222222222222222222222222222222222222222222222222222222222222222222222223333333333333333333333333333333333333333333333333333333333333333333333333333333333333

2222223333333333444444444455555555556666666666777777777788888888889999999999000000000011111111112222222222333333333344444444445555555555666666666677777777778888888888999999999900000000001111111111222222222233333333334444444444555555555566666666667777777777888888888899999999990000000000111111111122222222223333333333444444444455555555556666666666777777777788888

4567890123456789012345678901234567890123456789012345678901234567890123456789012345678901234567890123456789012345678901234567890123456789012345678901234567890123456789012345678901234567890123456789012345678901234567890123456789012345678901234567890123456789012345678901234567890123456789012345678901234567890123456789012345678901234567890123456789012345678901234

CRS TTCTTTCATGGGGAAGCAGATTTGGGTACCACCCAAGTATTGACTCACCCATCAACAACCGCTATGTATTTCGTACATTACTGCCAGCCACCATGAATATTGTACGGTACCATAAATACTTGACCACCTGTAGTACATAAAAACCCAATCCACATCAAAACCCCCTCCCCATGCTTACAAGCAAGTACAGCAATCAACCCTCAACTATCACACATCAACTGCAACTCCAAAGCCACCCCTCACCCACTAGGATACCAACAAACCTACCCACCCTTAACAGTACATAGTACATAAAGCCATTTACCGTACATAGCACATTACAGTCAAATCCCTTCTCGTCCCCATGGATGACCCCCCTCAG

>T164

T164.1.1.1_rib ............................................................................................................ACCATAAATACTTGACCACCTGTAGTAC

T164.1.1.2_rib ............................................................................................................

T164.1.1.3_rib ............................................................................................................

T164.1.1.4_rib ...............A............................................................................................

T164.1.1.5_rib ............................................................................................................

T164.1.2.1_rib ............................................................................................................

T164.1.2.2_rib ............................................................................................................

T164.1.2.3_rib ............................................................................................................

T164.1.2.4_rib ............................................................................................................

T164.1.2.5_rib ............................................................................................................

T164.2.1.1_rib ............................................................................................................

T164.2.1.2_rib ............................................................................................................

T164.2.1.3_rib ............................................................................................................

T164.2.1.4_rib ............................................................................................................

T164.2.1.5_rib ............................................................................................................

T164.2.2.1_rib ............................................................................................................

T164.2.2.2_rib ............................................................................................................

T164.2.2.3_rib ............................................................................................................

T164.2.2.4_rib ............................................................................................................

T164.2.2.5_rib ............................................................................................................

T164.1.1.1_rib CGCTATGTATTTCGTACATTACTGC.........................................................................................................................................................CCTCACCCACTAGGATACCA

T164.1.1.2_rib .........................................................................................................................................................

T164.1.1.3_rib .........................................................................................................................................................

T164.1.1.4_rib .........................................................................................................................................................

T164.1.1.5_rib .........................................................................................................................................................

T164.1.2.1_rib ....................T....................................................................................................................................

T164.1.2.2_rib .........................................................................................................................................................

T164.1.2.3_rib .........................................................................................................................................................

T164.1.2.4_rib .........................................................................................................................................................

T164.1.2.5_rib .........................................................................................................................................................

T164.2.1.1_rib .........................................................................................................................................................

T164.2.1.2_rib .........................................................................................................................................................

T164.2.1.3_rib .........................................................................................................................................................

T164.2.1.4_rib ...................................................................................................................................................A.....

T164.2.1.5_rib .........................................................................................................................................................

T164.2.2.1_rib .........................................................................................................................................................

T164.2.2.2_rib .........................................................................................................................................................

T164.2.2.3_rib .........................................................................................................................................................

T164.2.2.4_rib .........................................................................................................................................................

T164.2.2.5_rib .........................................................................................................................................................

T164.1.1.1_rib CAACTATCACACATCAACTGCAA.........................................................................................................................................

T164.1.1.2_rib .........................................................................................................................................

T164.1.1.3_rib .........................................................................................................................................

T164.1.1.4_rib .........................................................................................................................................

T164.1.1.5_rib .........................................................................................................................................

T164.1.2.1_rib .........................................................................................................................................

T164.1.2.2_rib .........................................................................................................................................

T164.1.2.3_rib .........................................................................................................................................

T164.1.2.4_rib .........................................................................................................................................

T164.1.2.5_rib .........................................................................................................................................

T164.2.1.1_rib .........................................................................................................................................

T164.2.1.2_rib .........................................................................................................................................

T164.2.1.3_rib .........................................................................................................................................

T164.2.1.4_rib .........................................................................................................................................

T164.2.1.5_rib .........................................................................................................................................

T164.2.2.1_rib .........................................................................................................................................

T164.2.2.2_rib .........................................................................................................................................

T164.2.2.3_rib .........................................................................................................................................

T164.2.2.4_rib .........................................................................................................................................

T164.2.2.5_rib .........................................................................................................................................

Consensus TTCTTTCATGGGGAAGCAGATTTGGGTACCACCCAAGTATTGACTCACCCATCAACAACCGCTATGTATTTCGTACATTACTGCCAGCCACCATGAATATTGTACGGTACCATAAATACTTGACCACCTGTAGTACATAAAAACCCAATCCACATCAAAACCCCCTCCCCATGCTTACAAGCAAGTACAGCAATCAACCCTCAACTATCACACATCAACTGCAACTCCAAAGCCACCCCTCACCCACTAGGATACCAACAAACCTACCCACCCTTAACAGTACATAGTACATAAAGCCATTTACCGTACATAGCACATTACAGTCAAATCCCTTCTCGTCCCCATGGATGACCCCCCTCAG

1111111111111111111111111111111111111111111111111111111111111111111111111111111111111111111111111111111111111111111111111111111111111111111111111111111111111111111111111111111111111111111111111111111111111111111111111111111111111111111111111111111111111111111111111111111111111111111111111111111111111111111111111111111111111111111111111111111111111111111111111

6666666666666666666666666666666666666666666666666666666666666666666666666666666666666666666666666666666666666666666666666666666666666666666666666666666666666666666666666666666666666666666666666666666666666666666666666666666666666666666666666666666666666666666666666666666666666666666666666666666666666666666666666666666666666666666666666666666666666666666666666

0000000000000000000000000000000000000000000000000000000000000000000000000000111111111111111111111111111111111111111111111111111111111111111111111111111111111111111111111111111122222222222222222222222222222222222222222222222222222222222222222222222222222222222222222222222222223333333333333333333333333333333333333333333333333333333333333333333333333333333333333

2222223333333333444444444455555555556666666666777777777788888888889999999999000000000011111111112222222222333333333344444444445555555555666666666677777777778888888888999999999900000000001111111111222222222233333333334444444444555555555566666666667777777777888888888899999999990000000000111111111122222222223333333333444444444455555555556666666666777777777788888

4567890123456789012345678901234567890123456789012345678901234567890123456789012345678901234567890123456789012345678901234567890123456789012345678901234567890123456789012345678901234567890123456789012345678901234567890123456789012345678901234567890123456789012345678901234567890123456789012345678901234567890123456789012345678901234567890123456789012345678901234

CRS TTCTTTCATGGGGAAGCAGATTTGGGTACCACCCAAGTATTGACTCACCCATCAACAACCGCTATGTATTTCGTACATTACTGCCAGCCACCATGAATATTGTACGGTACCATAAATACTTGACCACCTGTAGTACATAAAAACCCAATCCACATCAAAACCCCCTCCCCATGCTTACAAGCAAGTACAGCAATCAACCCTCAACTATCACACATCAACTGCAACTCCAAAGCCACCCCTCACCCACTAGGATACCAACAAACCTACCCACCCTTAACAGTACATAGTACATAAAGCCATTTACCGTACATAGCACATTACAGTCAAATCCCTTCTCGTCCCCATGGATGACCCCCCTCAG

>T164

T164.1.1.1_femur ............................................................................................................ACCATAAATACTTGACCACCTGTAGTAC

T164.1.1.2_femur ............................................................................................................

T164.1.1.3_femur ............................................................................................................

T164.1.1.4_femur ............................................................................................................

T164.1.1.5_femur ............................................................................................................

T164.1.2.1_femur ............................................................................................................

T164.1.2.2_femur ............................................................................................................

T164.1.2.3_femur ............................................................................................................

T164.1.2.4_femur ............................................................................................................

T164.1.2.5_femur ............................................................................................................

T164.2.1.1_femur ............................................................................................................

T164.2.1.2_femur ............................................................................................................

T164.2.1.3_femur ............................................................................................................

T164.2.1.4_femur ............................................................................................................

T164.2.1.5_femur ............................................................................................................

T164.2.2.1_femur ............................................................................................................

T164.2.2.2_femur ............................................................................................................

T164.2.2.3_femur ............................................................................................................

T164.2.2.4_femur ............................................................................................................

T164.2.2.5_femur ............................................................................................................

T164.1.1.1_femur CGCTATGTATTTCGTACATTACTGC.........................................................................................................................................................CCTCACCCACTAGGATACCA

T164.1.1.2_femur .........................................................................................................................................................

T164.1.1.3_femur .........................................................................................................................................................

T164.1.1.4_femur .........................................................................................................................................................

T164.1.1.5_femur .........................................................................................................................................................

T164.1.2.1_femur .........................................................................................................................................................

T164.1.2.2_femur .........................................................................................................................................................

T164.1.2.3_femur .........................................................................................................................................................

T164.1.2.4_femur .........................................................................................................................................................

T164.1.2.5_femur .........................................................................................................................................................

T164.2.1.1_femur .........................T...............................................................................................................................

T164.2.1.2_femur .........................................................................................................................................................

T164.2.1.3_femur .........................................................................................................................................................

T164.2.1.4_femur .........................................................................................................................................................

T164.2.1.5_femur .........................................................................................................................................................

T164.2.2.1_femur .........................................................................................................................................................

T164.2.2.2_femur .........................................................................................................................................................

T164.2.2.3_femur .........................................................................................................................................................

T164.2.2.4_femur .........................................................................................................................................................

T164.2.2.5_femur .........................................................................................................................................................

T164.1.1.1_femur CAACTATCACACATCAACTGCAA.........................................................................................................................................

T164.1.1.2_femur .........................................................................................................................................

T164.1.1.3_femur .........................................................................................................................................

T164.1.1.4_femur .........................................................................................................................................

T164.1.1.5_femur .........................................................................................................................................

T164.1.2.1_femur .........................................................................................................................................

T164.1.2.2_femur .........................................................................................................................................

T164.1.2.3_femur .........................................................................................................................................

T164.1.2.4_femur .........................................................................................................................................

T164.1.2.5_femur .........................................................................................................................................

T164.2.1.1_femur .........................................................................................................................................

T164.2.1.2_femur .........................................................................................................................................

T164.2.1.3_femur .........................................................................................................................................

T164.2.1.4_femur .........................................................................................................................................

T164.2.1.5_femur .........................................................................................................................................

T164.2.2.1_femur .........................................................................................................................................

T164.2.2.2_femur .........................................................................................................................................

T164.2.2.3_femur .........................................................................................................................................

T164.2.2.4_femur .........................................................................................................................................

T164.2.2.5_femur .......A.................................................................................................................................

Consensus TTCTTTCATGGGGAAGCAGATTTGGGTACCACCCAAGTATTGACTCACCCATCAACAACCGCTATGTATTTCGTACATTACTGCCAGCCACCATGAATATTGTACGGTACCATAAATACTTGACCACCTGTAGTACATAAAAACCCAATCCACATCAAAACCCCCTCCCCATGCTTACAAGCAAGTACAGCAATCAACCCTCAACTATCACACATCAACTGCAACTCCAAAGCCACCCCTCACCCACTAGGATACCAACAAACCTACCCACCCTTAACAGTACATAGTACATAAAGCCATTTACCGTACATAGCACATTACAGTCAAATCCCTTCTCGTCCCCATGGATGACCCCCCTCAG

1111111111111111111111111111111111111111111111111111111111111111111111111111111111111111111111111111111111111111111111111111111111111111111111111111111111111111111111111111111111111111111111111111111111111111111111111111111111111111111111111111111111111111111111111111111111111111111111111111111111111111111111111111111111111111111111111111111111111111111111111

6666666666666666666666666666666666666666666666666666666666666666666666666666666666666666666666666666666666666666666666666666666666666666666666666666666666666666666666666666666666666666666666666666666666666666666666666666666666666666666666666666666666666666666666666666666666666666666666666666666666666666666666666666666666666666666666666666666666666666666666666

0000000000000000000000000000000000000000000000000000000000000000000000000000111111111111111111111111111111111111111111111111111111111111111111111111111111111111111111111111111122222222222222222222222222222222222222222222222222222222222222222222222222222222222222222222222222223333333333333333333333333333333333333333333333333333333333333333333333333333333333333

2222223333333333444444444455555555556666666666777777777788888888889999999999000000000011111111112222222222333333333344444444445555555555666666666677777777778888888888999999999900000000001111111111222222222233333333334444444444555555555566666666667777777777888888888899999999990000000000111111111122222222223333333333444444444455555555556666666666777777777788888

4567890123456789012345678901234567890123456789012345678901234567890123456789012345678901234567890123456789012345678901234567890123456789012345678901234567890123456789012345678901234567890123456789012345678901234567890123456789012345678901234567890123456789012345678901234567890123456789012345678901234567890123456789012345678901234567890123456789012345678901234

CRS TTCTTTCATGGGGAAGCAGATTTGGGTACCACCCAAGTATTGACTCACCCATCAACAACCGCTATGTATTTCGTACATTACTGCCAGCCACCATGAATATTGTACGGTACCATAAATACTTGACCACCTGTAGTACATAAAAACCCAATCCACATCAAAACCCCCTCCCCATGCTTACAAGCAAGTACAGCAATCAACCCTCAACTATCACACATCAACTGCAACTCCAAAGCCACCCCTCACCCACTAGGATACCAACAAACCTACCCACCCTTAACAGTACATAGTACATAAAGCCATTTACCGTACATAGCACATTACAGTCAAATCCCTTCTCGTCCCCATGGATGACCCCCCTCAG

>T164

T164.1.1.1_ulna ............................................................................................................ACCATAAATACTTGACCACCTGTAGTAC

T164.1.1.2_ulna ............................................................................................................

T164.1.1.3_ulna ............................................................................................................

T164.1.1.4_ulna ............................................................................................................

T164.1.1.5_ulna ............................................................................................................

T164.1.2.1_ulna ............................................................................................................

T164.1.2.2_ulna ............................................................................................................

T164.1.2.3_ulna ............................................................................................................

T164.1.2.4_ulna ............................................................................................................

T164.1.2.5_ulna ............................................................................................................

T164.2.1.1_ulna ............................................................................................................

T164.2.1.2_ulna ............................................................................................................

T164.2.1.3_ulna ............................................................................................................

T164.2.1.4_ulna ............................................................................................................

T164.2.1.5_ulna ............................................................................................................

T164.2.2.1_ulna ............................................................................................................

T164.2.2.2_ulna ............................................................................................................

T164.2.2.3_ulna ..........................................................................................T.................

T164.2.2.4_ulna ............................................................................................................

T164.2.2.5_ulna ............................................................................................................

T164.1.1.1_ulna CGCTATGTATTTCGTACATTACTGC.........................................................................................................................................................CCTCACCCACTAGGATACCA

T164.1.1.2_ulna .........................................................................................................................................................

T164.1.1.3_ulna .........................................................................................................................................................

T164.1.1.4_ulna .........................................................................................................................................................

T164.1.1.5_ulna .........................................................................................................................................................

T164.1.2.1_ulna .........................................................................................................................................................

T164.1.2.2_ulna .........................................................................................................................................................

T164.1.2.3_ulna .........................................................................................................................................................

T164.1.2.4_ulna .........................................................................................................................................................

T164.1.2.5_ulna .........................................................................................................................................................

T164.2.1.1_ulna .........................................................................................................................................................

T164.2.1.2_ulna .........................................................................................................................................................

T164.2.1.3_ulna .........................................................................................................................................................

T164.2.1.4_ulna .........................................................................................................................................................

T164.2.1.5_ulna .........................................................................................................................................................

T164.2.2.1_ulna .........................................................................................................................................................

T164.2.2.2_ulna .........................................................................................................................................................

T164.2.2.3_ulna .........................................................................................................................................................

T164.2.2.4_ulna .........................................................................................................................................................

T164.2.2.5_ulna .........................................................................................................................................................

T164.1.1.1_ulna CAACTATCACACATCAACTGCAA.........................................................................................................................................

T164.1.1.2_ulna .........................................................................................................................................

T164.1.1.3_ulna .........................................................................................................................................

T164.1.1.4_ulna .........................................................................................................................................

T164.1.1.5_ulna .........................................................................................................................................

T164.1.2.1_ulna .........................................................................................................................................

T164.1.2.2_ulna .........................A...............................................................................................................

T164.1.2.3_ulna .........................................................................................................................................

T164.1.2.4_ulna .........................................................................................................................................

T164.1.2.5_ulna .........................................................................................................................................

T164.2.1.1_ulna .........................................................................................................................................

T164.2.1.2_ulna .........................................................................................................................................

T164.2.1.3_ulna .........................................................................................................................................

T164.2.1.4_ulna .........................................................................................................................................

T164.2.1.5_ulna .........................................................................................................................................

T164.2.2.1_ulna .........................................................................................................................................

T164.2.2.2_ulna .........................................................................................................................................

T164.2.2.3_ulna .........................................................................................................................................

T164.2.2.4_ulna .........................................................................................................................................

T164.2.2.5_ulna .........................................................................................................................................

Consensus TTCTTTCATGGGGAAGCAGATTTGGGTACCACCCAAGTATTGACTCACCCATCAACAACCGCTATGTATTTCGTACATTACTGCCAGCCACCATGAATATTGTACGGTACCATAAATACTTGACCACCTGTAGTACATAAAAACCCAATCCACATCAAAACCCCCTCCCCATGCTTACAAGCAAGTACAGCAATCAACCCTCAACTATCACACATCAACTGCAACTCCAAAGCCACCCCTCACCCACTAGGATACCAACAAACCTACCCACCCTTAACAGTACATAGTACATAAAGCCATTTACCGTACATAGCACATTACAGTCAAATCCCTTCTCGTCCCCATGGATGACCCCCCTCAG

1111111111111111111111111111111111111111111111111111111111111111111111111111111111111111111111111111111111111111111111111111111111111111111111111111111111111111111111111111111111111111111111111111111111111111111111111111111111111111111111111111111111111111111111111111111111111111111111111111111111111111111111111111111111111111111111111111111111111111111111111

6666666666666666666666666666666666666666666666666666666666666666666666666666666666666666666666666666666666666666666666666666666666666666666666666666666666666666666666666666666666666666666666666666666666666666666666666666666666666666666666666666666666666666666666666666666666666666666666666666666666666666666666666666666666666666666666666666666666666666666666666

0000000000000000000000000000000000000000000000000000000000000000000000000000111111111111111111111111111111111111111111111111111111111111111111111111111111111111111111111111111122222222222222222222222222222222222222222222222222222222222222222222222222222222222222222222222222223333333333333333333333333333333333333333333333333333333333333333333333333333333333333

2222223333333333444444444455555555556666666666777777777788888888889999999999000000000011111111112222222222333333333344444444445555555555666666666677777777778888888888999999999900000000001111111111222222222233333333334444444444555555555566666666667777777777888888888899999999990000000000111111111122222222223333333333444444444455555555556666666666777777777788888

4567890123456789012345678901234567890123456789012345678901234567890123456789012345678901234567890123456789012345678901234567890123456789012345678901234567890123456789012345678901234567890123456789012345678901234567890123456789012345678901234567890123456789012345678901234567890123456789012345678901234567890123456789012345678901234567890123456789012345678901234

CRS TTCTTTCATGGGGAAGCAGATTTGGGTACCACCCAAGTATTGACTCACCCATCAACAACCGCTATGTATTTCGTACATTACTGCCAGCCACCATGAATATTGTACGGTACCATAAATACTTGACCACCTGTAGTACATAAAAACCCAATCCACATCAAAACCCCCTCCCCATGCTTACAAGCAAGTACAGCAATCAACCCTCAACTATCACACATCAACTGCAACTCCAAAGCCACCCCTCACCCACTAGGATACCAACAAACCTACCCACCCTTAACAGTACATAGTACATAAAGCCATTTACCGTACATAGCACATTACAGTCAAATCCCTTCTCGTCCCCATGGATGACCCCCCTCAG

>T170

T170.1.1.1_tooth ............................................................................................................ACCATAAATACTTGACCACCTGTAGTAC

T170.1.1.2_tooth ............................................................................................................

T170.1.1.3_tooth ............................................................................................................

T170.1.1.4_tooth ............................................................................................................

T170.1.1.5_tooth ............................................................................................................

T170.1.2.1_tooth ............................................................................................................

T170.1.2.2_tooth ............................................................................................................

T170.1.2.3_tooth ............................................................................................................

T170.1.2.4_tooth ............................................................................................................

T170.1.2.5_tooth ............................................................................................................

T170.2.1.1_tooth ............................................................................................................

T170.2.1.2_tooth ............................................................................................................

T170.2.1.3_tooth ............................................................................................................

T170.2.1.4_tooth ............................................................................................................

T170.2.1.5_tooth ............................................................................................................

T170.2.2.1_tooth ............................................................................................................

T170.2.2.2_tooth ............................T...............................................................................

T170.2.2.3_tooth ............................................................................................................

T170.2.2.4_tooth ............................................................................................................

T170.2.2.5_tooth ............................................................................................................

T170.1.1.1_tooth CGCTATGTATTTCGTACATTACTGC.........................................................................................................................................................CCTCACCCACTAGGATACCA

T170.1.1.2_tooth .........................................................................................................................................................

T170.1.1.3_tooth .........................................................................................................................................................

T170.1.1.4_tooth .........................................................................................................................................................

T170.1.1.5_tooth .........................................................................................................................................................

T170.1.2.1_tooth .........................................................................................................................................................

T170.1.2.2_tooth .........................................................................................................................................................

T170.1.2.3_tooth .........................................................................................................................................................

T170.1.2.4_tooth .........................................................................................................................................................

T170.1.2.5_tooth .........................................................................................................................................................

T170.2.1.1_tooth ........................................................................................................................................A................

T170.2.1.2_tooth .........................................................................................................................................................

T170.2.1.3_tooth .........................................................................................................................................................

T170.2.1.4_tooth .........................................................................................................................................................

T170.2.1.5_tooth .........................................................................................................................................................

T170.2.2.1_tooth .........................................................................................................................................................

T170.2.2.2_tooth .........................................................................................................................................................

T170.2.2.3_tooth .........................................................................................................................................................

T170.2.2.4_tooth .........................................................................................................................................................

T170.2.2.5_tooth .........................................................................................................................................................

T170.1.1.1_tooth CAACTATCACACATCAACTGCAA.........................................................................................................................................

T170.1.1.2_tooth .........................................................................................................................................

T170.1.1.3_tooth .........................................................................................................................................

T170.1.1.4_tooth .........................................................................................................................................

T170.1.1.5_tooth .........................................................................................................................................

T170.1.2.1_tooth .........................................................................................................................................

T170.1.2.2_tooth .........................................................................................................................................

T170.1.2.3_tooth .........................................................................................................................................

T170.1.2.4_tooth .........................................................................................................................................

T170.1.2.5_tooth .........................................................................................................................................

T170.2.1.1_tooth .........................................................................................................................................

T170.2.1.2_tooth .........................................................................................................................................

T170.2.1.3_tooth .........................................................................................................................................

T170.2.1.4_tooth .........................................................................................................................................

T170.2.1.5_tooth .........................................................................................................................................

T170.2.2.1_tooth .........................................................................................................................................

T170.2.2.2_tooth .........................................................................................................................................

T170.2.2.3_tooth .........................................................................................................................................

T170.2.2.4_tooth .........................................................................................................................................

T170.2.2.5_tooth .........................................................................................................................................

Consensus TTCTTTCATGGGGAAGCAGATTTGGGTACCACCCAAGTATTGACTCACCCATCAACAACCGCTATGTATTTCGTACATTACTGCCAGCCACCATGAATATTGTACGGTACCATAAATACTTGACCACCTGTAGTACATAAAAACCCAATCCACATCAAAACCCCCTCCCCATGCTTACAAGCAAGTACAGCAATCAACCCTCAACTATCACACATCAACTGCAACTCCAAAGCCACCCCTCACCCACTAGGATACCAACAAACCTACCCACCCTTAACAGTACATAGTACATAAAGCCATTTACCGTACATAGCACATTACAGTCAAATCCCTTCTCGTCCCCATGGATGACCCCCCTCAG

1111111111111111111111111111111111111111111111111111111111111111111111111111111111111111111111111111111111111111111111111111111111111111111111111111111111111111111111111111111111111111111111111111111111111111111111111111111111111111111111111111111111111111111111111111111111111111111111111111111111111111111111111111111111111111111111111111111111111111111111111

6666666666666666666666666666666666666666666666666666666666666666666666666666666666666666666666666666666666666666666666666666666666666666666666666666666666666666666666666666666666666666666666666666666666666666666666666666666666666666666666666666666666666666666666666666666666666666666666666666666666666666666666666666666666666666666666666666666666666666666666666

0000000000000000000000000000000000000000000000000000000000000000000000000000111111111111111111111111111111111111111111111111111111111111111111111111111111111111111111111111111122222222222222222222222222222222222222222222222222222222222222222222222222222222222222222222222222223333333333333333333333333333333333333333333333333333333333333333333333333333333333333

2222223333333333444444444455555555556666666666777777777788888888889999999999000000000011111111112222222222333333333344444444445555555555666666666677777777778888888888999999999900000000001111111111222222222233333333334444444444555555555566666666667777777777888888888899999999990000000000111111111122222222223333333333444444444455555555556666666666777777777788888

4567890123456789012345678901234567890123456789012345678901234567890123456789012345678901234567890123456789012345678901234567890123456789012345678901234567890123456789012345678901234567890123456789012345678901234567890123456789012345678901234567890123456789012345678901234567890123456789012345678901234567890123456789012345678901234567890123456789012345678901234

CRS TTCTTTCATGGGGAAGCAGATTTGGGTACCACCCAAGTATTGACTCACCCATCAACAACCGCTATGTATTTCGTACATTACTGCCAGCCACCATGAATATTGTACGGTACCATAAATACTTGACCACCTGTAGTACATAAAAACCCAATCCACATCAAAACCCCCTCCCCATGCTTACAAGCAAGTACAGCAATCAACCCTCAACTATCACACATCAACTGCAACTCCAAAGCCACCCCTCACCCACTAGGATACCAACAAACCTACCCACCCTTAACAGTACATAGTACATAAAGCCATTTACCGTACATAGCACATTACAGTCAAATCCCTTCTCGTCCCCATGGATGACCCCCCTCAG

>T170

T170.1.1.1_rib ............................................................................................................ACCATAAATACTTGACCACCTGTAGTAC

T170.1.1.2_rib ............................................................................................................

T170.1.1.3_rib ............................................................................................................

T170.1.1.4_rib ............................................................................................................

T170.1.1.5_rib ............................................................................................................

T170.1.2.1_rib ............................................................................................................

T170.1.2.2_rib ............................................................................................................

T170.1.2.3_rib ............................................................................................................

T170.1.2.4_rib ............................................................................................................

T170.1.2.5_rib ............................................................................................................

T170.2.1.1_rib ............................................................................................................

T170.2.1.2_rib ............................................................................................................

T170.2.1.3_rib ............................................................................................................

T170.2.1.4_rib ............................................................................................................

T170.2.1.5_rib ............................................................................................................

T170.2.2.1_rib ............................................................................................................

T170.2.2.2_rib ............................................................................................................

T170.2.2.3_rib ............................................................................................................

T170.2.2.4_rib ............................................................................................................

T170.2.2.5_rib ............................................................................................................

T170.1.1.1_rib CGCTATGTATTTCGTACATTACTGC.........................................................................................................................................................CCTCACCCACTAGGATACCA

T170.1.1.2_rib .........................................................................................................................................................

T170.1.1.3_rib .........................................................................................................................................................

T170.1.1.4_rib ........................................................................................................................T................................

T170.1.1.5_rib .........................................................................................................................................................

T170.1.2.1_rib .........................................................................................................................................................

T170.1.2.2_rib .........................................................................................................................................................

T170.1.2.3_rib .........................................................................................................................................................

T170.1.2.4_rib .........................................................................................................................................................

T170.1.2.5_rib .........................................................................................................................................................

T170.2.1.1_rib .........................................................................................................................................................

T170.2.1.2_rib ..................................T......................................................................................................................

T170.2.1.3_rib .........................................................................................................................................................

T170.2.1.4_rib .........................................................................................................................................................

T170.2.1.5_rib .........................................................................................................................................................

T170.2.2.1_rib .........................................................................................................................................................

T170.2.2.2_rib .........................................................................................................................................................

T170.2.2.3_rib .........................................................................................................................................................

T170.2.2.4_rib .........................................................................................................................................................

T170.2.2.5_rib .........................................................................................................................................................

T170.1.1.1_rib CAACTATCACACATCAACTGCAA.........................................................................................................................................

T170.1.1.2_rib .........................................................................................................................................

T170.1.1.3_rib .........................................................................................................................................

T170.1.1.4_rib .........................................................................................................................................

T170.1.1.5_rib .........................................................................................................................................

T170.1.2.1_rib .................................................................................................A.......................................

T170.1.2.2_rib .........................................................................................................................................

T170.1.2.3_rib .........................................................................................................................................

T170.1.2.4_rib .........................................................................................................................................

T170.1.2.5_rib .........................................................................................................................................

T170.2.1.1_rib .........................................................................................................................................

T170.2.1.2_rib .........................................................................................................................................

T170.2.1.3_rib .........................................................................................................................................

T170.2.1.4_rib .........................................................................................................................................

T170.2.1.5_rib .........................................................................................................................................

T170.2.2.1_rib .........................................................................................................................................

T170.2.2.2_rib .........................................................................................................................................

T170.2.2.3_rib .........................................................................................................................................

T170.2.2.4_rib .........................................................................................................................................

T170.2.2.5_rib .........................................................................................................................................

Consensus TTCTTTCATGGGGAAGCAGATTTGGGTACCACCCAAGTATTGACTCACCCATCAACAACCGCTATGTATTTCGTACATTACTGCCAGCCACCATGAATATTGTACGGTACCATAAATACTTGACCACCTGTAGTACATAAAAACCCAATCCACATCAAAACCCCCTCCCCATGCTTACAAGCAAGTACAGCAATCAACCCTCAACTATCACACATCAACTGCAACTCCAAAGCCACCCCTCACCCACTAGGATACCAACAAACCTACCCACCCTTAACAGTACATAGTACATAAAGCCATTTACCGTACATAGCACATTACAGTCAAATCCCTTCTCGTCCCCATGGATGACCCCCCTCAG

1111111111111111111111111111111111111111111111111111111111111111111111111111111111111111111111111111111111111111111111111111111111111111111111111111111111111111111111111111111111111111111111111111111111111111111111111111111111111111111111111111111111111111111111111111111111111111111111111111111111111111111111111111111111111111111111111111111111111111111111111

6666666666666666666666666666666666666666666666666666666666666666666666666666666666666666666666666666666666666666666666666666666666666666666666666666666666666666666666666666666666666666666666666666666666666666666666666666666666666666666666666666666666666666666666666666666666666666666666666666666666666666666666666666666666666666666666666666666666666666666666666

0000000000000000000000000000000000000000000000000000000000000000000000000000111111111111111111111111111111111111111111111111111111111111111111111111111111111111111111111111111122222222222222222222222222222222222222222222222222222222222222222222222222222222222222222222222222223333333333333333333333333333333333333333333333333333333333333333333333333333333333333

2222223333333333444444444455555555556666666666777777777788888888889999999999000000000011111111112222222222333333333344444444445555555555666666666677777777778888888888999999999900000000001111111111222222222233333333334444444444555555555566666666667777777777888888888899999999990000000000111111111122222222223333333333444444444455555555556666666666777777777788888

4567890123456789012345678901234567890123456789012345678901234567890123456789012345678901234567890123456789012345678901234567890123456789012345678901234567890123456789012345678901234567890123456789012345678901234567890123456789012345678901234567890123456789012345678901234567890123456789012345678901234567890123456789012345678901234567890123456789012345678901234

CRS TTCTTTCATGGGGAAGCAGATTTGGGTACCACCCAAGTATTGACTCACCCATCAACAACCGCTATGTATTTCGTACATTACTGCCAGCCACCATGAATATTGTACGGTACCATAAATACTTGACCACCTGTAGTACATAAAAACCCAATCCACATCAAAACCCCCTCCCCATGCTTACAAGCAAGTACAGCAATCAACCCTCAACTATCACACATCAACTGCAACTCCAAAGCCACCCCTCACCCACTAGGATACCAACAAACCTACCCACCCTTAACAGTACATAGTACATAAAGCCATTTACCGTACATAGCACATTACAGTCAAATCCCTTCTCGTCCCCATGGATGACCCCCCTCAG

>T170

T170.1.1.1_femur ............................................................................................................ACCATAAATACTTGACCACCTGTAGTAC

T170.1.1.2_femur ............................................................................................................

T170.1.1.3_femur ............................................................................................................

T170.1.1.4_femur ............................................................................................................

T170.1.1.5_femur ............................................................................................................

T170.1.2.1_femur ............................................................................................................

T170.1.2.2_femur ............................................................................................................

T170.1.2.3_femur .....................................................................................................A......

T170.1.2.4_femur ............................................................................................................

T170.1.2.5_femur ............................................................................................................

T170.2.1.1_femur ............................................................................................................

T170.2.1.2_femur ............................................................................................................

T170.2.1.3_femur ............................................................................................................

T170.2.1.4_femur ............................................................................................................

T170.2.1.5_femur ............................................................................................................

T170.2.2.1_femur ............................................................................................................

T170.2.2.2_femur ............................................................................................................

T170.2.2.3_femur ............................................................................................................

T170.2.2.4_femur ............................................................................................................

T170.2.2.5_femur ............................................................................................................

T170.1.1.1_femur CGCTATGTATTTCGTACATTACTGC.........................................................................................................................................................CCTCACCCACTAGGATACCA

T170.1.1.2_femur .........................................................................................................................................................

T170.1.1.3_femur .........................................................................................................................................................

T170.1.1.4_femur .........................................................................................................................................................

T170.1.1.5_femur .........................................................................................................................................................

T170.1.2.1_femur .........................................................................................................................................................

T170.1.2.2_femur .........................................................................................................................................................

T170.1.2.3_femur .........................................................................................................................................................

T170.1.2.4_femur .........................................................................................................................................................

T170.1.2.5_femur .........................................................................................................................................................

T170.2.1.1_femur .........................................................................................................................................................

T170.2.1.2_femur .........................................................................................................................................................

T170.2.1.3_femur .........................................................................................................................................................

T170.2.1.4_femur ..................................T......................................................................................................................

T170.2.1.5_femur .........................................................................................................................................................

T170.2.2.1_femur .........................................................................................................................................................

T170.2.2.2_femur .........................................................................................................................................................

T170.2.2.3_femur .........................................................................................................................................................

T170.2.2.4_femur .........................................................................................................................................................

T170.2.2.5_femur .........................................................................................................................................................

T170.1.1.1_femur CAACTATCACACATCAACTGCAA.........................................................................................................................................

T170.1.1.2_femur .........................................................................................................................................

T170.1.1.3_femur .........................................................................................................................................

T170.1.1.4_femur .........................................................................................................................................

T170.1.1.5_femur .........................................................................................................................................

T170.1.2.1_femur .........................................................................................................................................

T170.1.2.2_femur .........................................................................................................................................

T170.1.2.3_femur .........................................................................................................................................

T170.1.2.4_femur .........................................................................................................................................

T170.1.2.5_femur .........................................................................................................................................

T170.2.1.1_femur .........................................................................................................................................

T170.2.1.2_femur .........................................................................................................................................

T170.2.1.3_femur .........................................................................................................................................

T170.2.1.4_femur .........................................................................................................................................

T170.2.1.5_femur .........................................................................................................................................

T170.2.2.1_femur .........................................................................................................................................

T170.2.2.2_femur .........................................................................................................................................

T170.2.2.3_femur .........................................................................................................................................

T170.2.2.4_femur .........................................................................................................................................

T170.2.2.5_femur .........................................................................................................................................

Consensus TTCTTTCATGGGGAAGCAGATTTGGGTACCACCCAAGTATTGACTCACCCATCAACAACCGCTATGTATTTCGTACATTACTGCCAGCCACCATGAATATTGTACGGTACCATAAATACTTGACCACCTGTAGTACATAAAAACCCAATCCACATCAAAACCCCCTCCCCATGCTTACAAGCAAGTACAGCAATCAACCCTCAACTATCACACATCAACTGCAACTCCAAAGCCACCCCTCACCCACTAGGATACCAACAAACCTACCCACCCTTAACAGTACATAGTACATAAAGCCATTTACCGTACATAGCACATTACAGTCAAATCCCTTCTCGTCCCCATGGATGACCCCCCTCAG

1111111111111111111111111111111111111111111111111111111111111111111111111111111111111111111111111111111111111111111111111111111111111111111111111111111111111111111111111111111111111111111111111111111111111111111111111111111111111111111111111111111111111111111111111111111111111111111111111111111111111111111111111111111111111111111111111111111111111111111111111

6666666666666666666666666666666666666666666666666666666666666666666666666666666666666666666666666666666666666666666666666666666666666666666666666666666666666666666666666666666666666666666666666666666666666666666666666666666666666666666666666666666666666666666666666666666666666666666666666666666666666666666666666666666666666666666666666666666666666666666666666

0000000000000000000000000000000000000000000000000000000000000000000000000000111111111111111111111111111111111111111111111111111111111111111111111111111111111111111111111111111122222222222222222222222222222222222222222222222222222222222222222222222222222222222222222222222222223333333333333333333333333333333333333333333333333333333333333333333333333333333333333

2222223333333333444444444455555555556666666666777777777788888888889999999999000000000011111111112222222222333333333344444444445555555555666666666677777777778888888888999999999900000000001111111111222222222233333333334444444444555555555566666666667777777777888888888899999999990000000000111111111122222222223333333333444444444455555555556666666666777777777788888

4567890123456789012345678901234567890123456789012345678901234567890123456789012345678901234567890123456789012345678901234567890123456789012345678901234567890123456789012345678901234567890123456789012345678901234567890123456789012345678901234567890123456789012345678901234567890123456789012345678901234567890123456789012345678901234567890123456789012345678901234

CRS TTCTTTCATGGGGAAGCAGATTTGGGTACCACCCAAGTATTGACTCACCCATCAACAACCGCTATGTATTTCGTACATTACTGCCAGCCACCATGAATATTGTACGGTACCATAAATACTTGACCACCTGTAGTACATAAAAACCCAATCCACATCAAAACCCCCTCCCCATGCTTACAAGCAAGTACAGCAATCAACCCTCAACTATCACACATCAACTGCAACTCCAAAGCCACCCCTCACCCACTAGGATACCAACAAACCTACCCACCCTTAACAGTACATAGTACATAAAGCCATTTACCGTACATAGCACATTACAGTCAAATCCCTTCTCGTCCCCATGGATGACCCCCCTCAG

>T170

T170.1.1.1_ulna ............................................................................................................ACCATAAATACTTGACCACCTGTAGTAC

T170.1.1.2_ulna ............................................................................................................

T170.1.1.3_ulna ............................................................................................................

T170.1.1.4_ulna ............................................................................................................

T170.1.1.5_ulna ............................................................................................................

T170.1.2.1_ulna ............................................................................................................

T170.1.2.2_ulna ............................................................................................................

T170.1.2.3_ulna ............................................................................................................

T170.1.2.4_ulna ............................................................................................................

T170.1.2.5_ulna ............................................................................................................

T170.2.1.1_ulna ............................................................................................................

T170.2.1.2_ulna ............................................................................................................

T170.2.1.3_ulna ............................................................................................................

T170.2.1.4_ulna ............................................................................................................

T170.2.1.5_ulna ............................................................................................................

T170.2.2.1_ulna ............................................................................................................

T170.2.2.2_ulna ............................................................................................................

T170.2.2.3_ulna ............................................................................................................

T170.2.2.4_ulna ............................................................................................................

T170.2.2.5_ulna ............................................................................................................

T170.1.1.1_ulna CGCTATGTATTTCGTACATTACTGC.........................................................................................................................................................CCTCACCCACTAGGATACCA

T170.1.1.2_ulna .........................................................................................................................................................

T170.1.1.3_ulna .........................................................................................................................................................

T170.1.1.4_ulna ..............................................................................................................T..........................................

T170.1.1.5_ulna .........................................................................................................................................................

T170.1.2.1_ulna .........................................................................................................................................................

T170.1.2.2_ulna .........................................................................................................................................................

T170.1.2.3_ulna .........................................................................................................................................................

T170.1.2.4_ulna .........................................................................................................................................................

T170.1.2.5_ulna .........................................................................................................................................................

T170.2.1.1_ulna .........................................................................................................................................................

T170.2.1.2_ulna .........................................................................................................................................................

T170.2.1.3_ulna .................A.......................................................................................................................................

T170.2.1.4_ulna .........................................................................................................................................................

T170.2.1.5_ulna .........................................................................................................................................................

T170.2.2.1_ulna .........................................................................................................................................................

T170.2.2.2_ulna .........................................................................................................................................................

T170.2.2.3_ulna .........................................................................................................................................................

T170.2.2.4_ulna .........................................................................................................................................................

T170.2.2.5_ulna .........................................................................................................................................................

T170.1.1.1_ulna CAACTATCACACATCAACTGCAA.........................................................................................................................................

T170.1.1.2_ulna .........................................................................................................................................

T170.1.1.3_ulna .........................................................................................................................................

T170.1.1.4_ulna .........................................................................................................................................

T170.1.1.5_ulna ....................................................................................................T....................................

T170.1.2.1_ulna .........................................................................................................................................

T170.1.2.2_ulna .........................................................................................................................................

T170.1.2.3_ulna .........................................................................................................................................

T170.1.2.4_ulna .........................................................................................................................................

T170.1.2.5_ulna .........................................................................................................................................

T170.2.1.1_ulna .........................................................................................................................................

T170.2.1.2_ulna .........................................................................................................................................

T170.2.1.3_ulna .........................................................................................................................................

T170.2.1.4_ulna .........................................................................................................................................

T170.2.1.5_ulna .........................................................................................................................................

T170.2.2.1_ulna .........................................................................................................................................

T170.2.2.2_ulna .........................................................................................................................................

T170.2.2.3_ulna .........................................................................................................................................

T170.2.2.4_ulna .........................................................................................................................................

T170.2.2.5_ulna .........................................................................................................................................

Consensus TTCTTTCATGGGGAAGCAGATTTGGGTACCACCCAAGTATTGACTCACCCATCAACAACCGCTATGTATTTCGTACATTACTGCCAGCCACCATGAATATTGTACGGTACCATAAATACTTGACCACCTGTAGTACATAAAAACCCAATCCACATCAAAACCCCCTCCCCATGCTTACAAGCAAGTACAGCAATCAACCCTCAACTATCACACATCAACTGCAACTCCAAAGCCACCCCTCACCCACTAGGATACCAACAAACCTACCCACCCTTAACAGTACATAGTACATAAAGCCATTTACCGTACATAGCACATTACAGTCAAATCCCTTCTCGTCCCCATGGATGACCCCCCTCAG

1111111111111111111111111111111111111111111111111111111111111111111111111111111111111111111111111111111111111111111111111111111111111111111111111111111111111111111111111111111111111111111111111111111111111111111111111111111111111111111111111111111111111111111111111111111111111111111111111111111111111111111111111111111111111111111111111111111111111111111111111

6666666666666666666666666666666666666666666666666666666666666666666666666666666666666666666666666666666666666666666666666666666666666666666666666666666666666666666666666666666666666666666666666666666666666666666666666666666666666666666666666666666666666666666666666666666666666666666666666666666666666666666666666666666666666666666666666666666666666666666666666

0000000000000000000000000000000000000000000000000000000000000000000000000000111111111111111111111111111111111111111111111111111111111111111111111111111111111111111111111111111122222222222222222222222222222222222222222222222222222222222222222222222222222222222222222222222222223333333333333333333333333333333333333333333333333333333333333333333333333333333333333

2222223333333333444444444455555555556666666666777777777788888888889999999999000000000011111111112222222222333333333344444444445555555555666666666677777777778888888888999999999900000000001111111111222222222233333333334444444444555555555566666666667777777777888888888899999999990000000000111111111122222222223333333333444444444455555555556666666666777777777788888

4567890123456789012345678901234567890123456789012345678901234567890123456789012345678901234567890123456789012345678901234567890123456789012345678901234567890123456789012345678901234567890123456789012345678901234567890123456789012345678901234567890123456789012345678901234567890123456789012345678901234567890123456789012345678901234567890123456789012345678901234

CRS TTCTTTCATGGGGAAGCAGATTTGGGTACCACCCAAGTATTGACTCACCCATCAACAACCGCTATGTATTTCGTACATTACTGCCAGCCACCATGAATATTGTACGGTACCATAAATACTTGACCACCTGTAGTACATAAAAACCCAATCCACATCAAAACCCCCTCCCCATGCTTACAAGCAAGTACAGCAATCAACCCTCAACTATCACACATCAACTGCAACTCCAAAGCCACCCCTCACCCACTAGGATACCAACAAACCTACCCACCCTTAACAGTACATAGTACATAAAGCCATTTACCGTACATAGCACATTACAGTCAAATCCCTTCTCGTCCCCATGGATGACCCCCCTCAG

>T176

T176.1.1.1_tooth ............................................................................................................ACCATAAATACTTGACCACCTGTAGTAC

T176.1.1.2_tooth ............................................................................................................

T176.1.1.3_tooth ............................................................................................................

T176.1.1.4_tooth ............................................................................................................

T176.1.1.5_tooth ............................................................................................................

T176.1.2.1_tooth ............................................................................................................

T176.1.2.2_tooth ............................................................................................................

T176.1.2.3_tooth ............................................................................................................

T176.1.2.4_tooth ............................................................................................................

T176.1.2.5_tooth ............................................................................................................

T176.2.1.1_tooth ............................................................................................................

T176.2.1.2_tooth ............................................................................................................

T176.2.1.3_tooth ............................................................................................................

T176.2.1.4_tooth ............................................................................................................

T176.2.1.5_tooth ............................................................................................................

T176.2.2.1_tooth ............................................................................................................

T176.2.2.2_tooth ............................................................................................................

T176.2.2.3_tooth ............................................................................................................

T176.2.2.4_tooth ............................................................................................................

T176.2.2.5_tooth ............................................................................................................

T176.1.1.1_tooth CGCTATGTATTTCGTACATTACTGC................................................................C........................................................................................CCTCACCCACTAGGATACCA

T176.1.1.2_tooth ................................................................C........................................................................................

T176.1.1.3_tooth ................................................................C........................................................................................

T176.1.1.4_tooth ................................................................C........................................................................................

T176.1.1.5_tooth ................................................................C........................................................................................

T176.1.2.1_tooth ................................................................C........................................................................................

T176.1.2.2_tooth ................................................................C........................................................................................

T176.1.2.3_tooth ..................................T.............................C........................................................................................

T176.1.2.4_tooth ................................................................C........................................................................................

T176.1.2.5_tooth ................................................................C........................................................................................

T176.2.1.1_tooth ................................................................C........................................................................................

T176.2.1.2_tooth ................................................................C...............................A........................................................

T176.2.1.3_tooth ................................................................C........................................................................................

T176.2.1.4_tooth ................................................................C........................................................................................

T176.2.1.5_tooth ................................................................C........................................................................................

T176.2.2.1_tooth ................................................................C........................................................................................

T176.2.2.2_tooth ................................................................C........................................................................................

T176.2.2.3_tooth ................................................................C........................................................................................

T176.2.2.4_tooth ................................................................C........................................................................................

T176.2.2.5_tooth ................................................................C........................................................................................

T176.1.1.1_tooth CAACTATCACACATCAACTGCAA.........................................................................................................................................

T176.1.1.2_tooth .........................................................................................................................................

T176.1.1.3_tooth .........................................................................................................................................

T176.1.1.4_tooth .........................................................................................................................................

T176.1.1.5_tooth .........................................................................................................................................

T176.1.2.1_tooth .........................................................................................................................................

T176.1.2.2_tooth .........................................................................................................................................

T176.1.2.3_tooth .........................................................................................................................................

T176.1.2.4_tooth .........................................................................................................................................

T176.1.2.5_tooth .........................................................................................................................................

T176.2.1.1_tooth .........................................................................................................................................

T176.2.1.2_tooth .........................................................................................................................................

T176.2.1.3_tooth .........................................................................................................................................

T176.2.1.4_tooth .........................................................................................................................................

T176.2.1.5_tooth .........................................................................................................................................

T176.2.2.1_tooth ................................................................................................T........................................

T176.2.2.2_tooth .........................................................................................................................................

T176.2.2.3_tooth .........................................................................................................................................

T176.2.2.4_tooth .........................................................................................................................................

T176.2.2.5_tooth .........................................................................................................................................

Consensus TTCTTTCATGGGGAAGCAGATTTGGGTACCACCCAAGTATTGACTCACCCATCAACAACCGCTATGTATTTCGTACATTACTGCCAGCCACCATGAATATTGTACGGTACCATAAATACTTGACCACCTGTAGTACATAAAAACCCAACCCACATCAAAACCCCCTCCCCATGCTTACAAGCAAGTACAGCAATCAACCCTCAACTATCACACATCAACTGCAACTCCAAAGCCACCCCTCACCCACTAGGATACCAACAAACCTACCCACCCTTAACAGTACATAGTACATAAAGCCATTTACCGTACATAGCACATTACAGTCAAATCCCTTCTCGTCCCCATGGATGACCCCCCTCAG

1111111111111111111111111111111111111111111111111111111111111111111111111111111111111111111111111111111111111111111111111111111111111111111111111111111111111111111111111111111111111111111111111111111111111111111111111111111111111111111111111111111111111111111111111111111111111111111111111111111111111111111111111111111111111111111111111111111111111111111111111

6666666666666666666666666666666666666666666666666666666666666666666666666666666666666666666666666666666666666666666666666666666666666666666666666666666666666666666666666666666666666666666666666666666666666666666666666666666666666666666666666666666666666666666666666666666666666666666666666666666666666666666666666666666666666666666666666666666666666666666666666

0000000000000000000000000000000000000000000000000000000000000000000000000000111111111111111111111111111111111111111111111111111111111111111111111111111111111111111111111111111122222222222222222222222222222222222222222222222222222222222222222222222222222222222222222222222222223333333333333333333333333333333333333333333333333333333333333333333333333333333333333

2222223333333333444444444455555555556666666666777777777788888888889999999999000000000011111111112222222222333333333344444444445555555555666666666677777777778888888888999999999900000000001111111111222222222233333333334444444444555555555566666666667777777777888888888899999999990000000000111111111122222222223333333333444444444455555555556666666666777777777788888

4567890123456789012345678901234567890123456789012345678901234567890123456789012345678901234567890123456789012345678901234567890123456789012345678901234567890123456789012345678901234567890123456789012345678901234567890123456789012345678901234567890123456789012345678901234567890123456789012345678901234567890123456789012345678901234567890123456789012345678901234

CRS TTCTTTCATGGGGAAGCAGATTTGGGTACCACCCAAGTATTGACTCACCCATCAACAACCGCTATGTATTTCGTACATTACTGCCAGCCACCATGAATATTGTACGGTACCATAAATACTTGACCACCTGTAGTACATAAAAACCCAATCCACATCAAAACCCCCTCCCCATGCTTACAAGCAAGTACAGCAATCAACCCTCAACTATCACACATCAACTGCAACTCCAAAGCCACCCCTCACCCACTAGGATACCAACAAACCTACCCACCCTTAACAGTACATAGTACATAAAGCCATTTACCGTACATAGCACATTACAGTCAAATCCCTTCTCGTCCCCATGGATGACCCCCCTCAG

>T176

T176.1.1.1_rib ................T...........................................................................................ACCATAAATACTTGACCACCTGTAGTAC

T176.1.1.2_rib ............................................................................................................

T176.1.1.3_rib ............................................................................................................

T176.1.1.4_rib ............................................................................................................

T176.1.1.5_rib ............................................................................................................

T176.1.2.1_rib ............................................................................................................

T176.1.2.2_rib ............................................................................................................

T176.1.2.3_rib ............................................................................................................

T176.1.2.4_rib ............................................................................................................

T176.1.2.5_rib ............................................................................................................

T176.2.1.1_rib ............................................................................................................

T176.2.1.2_rib ............................................................................................................

T176.2.1.3_rib ............................................................................................................

T176.2.1.4_rib ............................................................................................................

T176.2.1.5_rib ......................................................................................A.....................

T176.2.2.1_rib ............................................................................................................

T176.2.2.2_rib ............................................................................................................

T176.2.2.3_rib ............................................................................................................

T176.2.2.4_rib ............................................................................................................

T176.2.2.5_rib ............................................................................................................

T176.1.1.1_rib CGCTATGTATTTCGTACATTACTGC.........................................................................................................................................................CCTCACCCACTAGGATACCA

T176.1.1.2_rib .........................................................................................................................................................

T176.1.1.3_rib .........................................................................................................................................................

T176.1.1.4_rib .........................................................................................................................................................

T176.1.1.5_rib .........................................................................................................................................................

T176.1.2.1_rib .....................................A...................................................................................................................

T176.1.2.2_rib .........................................................................................................................................................

T176.1.2.3_rib .........................................................................................................................................................

T176.1.2.4_rib ...................................................................................................................................T.....................

T176.1.2.5_rib .........................................................................................................................................................

T176.2.1.1_rib .........................................................................................................................................................

T176.2.1.2_rib .........................................................................................................................................................

T176.2.1.3_rib .........................................................................................................................................................

T176.2.1.4_rib .........................................................................................................................................................

T176.2.1.5_rib .........................................................................................................................................................

T176.2.2.1_rib ................................................A........................................................................................................

T176.2.2.2_rib .........................................................................................................................................................

T176.2.2.3_rib ....................................................................................................................................................T....

T176.2.2.4_rib .........................................................................................................................................................

T176.2.2.5_rib .........................................................................................................................................................

T176.1.1.1_rib CAACTATCACACATCAACTGCAA.........................................................................................................................................

T176.1.1.2_rib .........................................................................................................................................

T176.1.1.3_rib .........................................................................................................................................

T176.1.1.4_rib .................................................................T.......................................................................

T176.1.1.5_rib .........................................................................................................................................

T176.1.2.1_rib .........................................................................................................................................

T176.1.2.2_rib .........................................................................................................................................

T176.1.2.3_rib .........................................................................................................................................

T176.1.2.4_rib .........................A...............................................................................................................

T176.1.2.5_rib .........................................................................................................................................

T176.2.1.1_rib .........................................................................................................................................

T176.2.1.2_rib .........................................................................................................................................

T176.2.1.3_rib .........................................................................................................................................

T176.2.1.4_rib .........................................................................................................................................

T176.2.1.5_rib .........................................................................................................................................

T176.2.2.1_rib .........................................................................................................................................

T176.2.2.2_rib .........................................................................................................................................

T176.2.2.3_rib .........................................................................................................................................

T176.2.2.4_rib ....................................................................................................T....................................

T176.2.2.5_rib .........................................................................................................................................

Consensus TTCTTTCATGGGGAAGCAGATTTGGGTACCACCCAAGTATTGACTCACCCATCAACAACCGCTATGTATTTCGTACATTACTGCCAGCCACCATGAATATTGTACGGTACCATAAATACTTGACCACCTGTAGTACATAAAAACCCAATCCACATCAAAACCCCCTCCCCATGCTTACAAGCAAGTACAGCAATCAACCCTCAACTATCACACATCAACTGCAACTCCAAAGCCACCCCTCACCCACTAGGATACCAACAAACCTACCCACCCTTAACAGTACATAGTACATAAAGCCATTTACCGTACATAGCACATTACAGTCAAATCCCTTCTCGTCCCCATGGATGACCCCCCTCAG

1111111111111111111111111111111111111111111111111111111111111111111111111111111111111111111111111111111111111111111111111111111111111111111111111111111111111111111111111111111111111111111111111111111111111111111111111111111111111111111111111111111111111111111111111111111111111111111111111111111111111111111111111111111111111111111111111111111111111111111111111

6666666666666666666666666666666666666666666666666666666666666666666666666666666666666666666666666666666666666666666666666666666666666666666666666666666666666666666666666666666666666666666666666666666666666666666666666666666666666666666666666666666666666666666666666666666666666666666666666666666666666666666666666666666666666666666666666666666666666666666666666

0000000000000000000000000000000000000000000000000000000000000000000000000000111111111111111111111111111111111111111111111111111111111111111111111111111111111111111111111111111122222222222222222222222222222222222222222222222222222222222222222222222222222222222222222222222222223333333333333333333333333333333333333333333333333333333333333333333333333333333333333

2222223333333333444444444455555555556666666666777777777788888888889999999999000000000011111111112222222222333333333344444444445555555555666666666677777777778888888888999999999900000000001111111111222222222233333333334444444444555555555566666666667777777777888888888899999999990000000000111111111122222222223333333333444444444455555555556666666666777777777788888

4567890123456789012345678901234567890123456789012345678901234567890123456789012345678901234567890123456789012345678901234567890123456789012345678901234567890123456789012345678901234567890123456789012345678901234567890123456789012345678901234567890123456789012345678901234567890123456789012345678901234567890123456789012345678901234567890123456789012345678901234

CRS TTCTTTCATGGGGAAGCAGATTTGGGTACCACCCAAGTATTGACTCACCCATCAACAACCGCTATGTATTTCGTACATTACTGCCAGCCACCATGAATATTGTACGGTACCATAAATACTTGACCACCTGTAGTACATAAAAACCCAATCCACATCAAAACCCCCTCCCCATGCTTACAAGCAAGTACAGCAATCAACCCTCAACTATCACACATCAACTGCAACTCCAAAGCCACCCCTCACCCACTAGGATACCAACAAACCTACCCACCCTTAACAGTACATAGTACATAAAGCCATTTACCGTACATAGCACATTACAGTCAAATCCCTTCTCGTCCCCATGGATGACCCCCCTCAG

>T176

T176.1.1.1_femur ............................................................................................................ACCATAAATACTTGACCACCTGTAGTAC

T176.1.1.2_femur ............................................................................................................

T176.1.1.3_femur ............................T...............................................................................

T176.1.1.4_femur ............................................................................................................

T176.1.1.5_femur ............................................................................................................

T176.1.2.1_femur ............................................................................................................

T176.1.2.2_femur ............................................................................................................

T176.1.2.3_femur ............................................................................................................

T176.1.2.4_femur ............................................................................................................

T176.1.2.5_femur ............................................................................................................

T176.2.1.1_femur ............................................................................................................

T176.2.1.2_femur ............................................................................................................

T176.2.1.3_femur ..........................................................................................T.................

T176.2.1.4_femur ............................................................................................................

T176.2.1.5_femur ............................................................................................................

T176.2.2.1_femur ............................................................................................................

T176.2.2.2_femur ............................................................................................................

T176.2.2.3_femur ............................................................................................................

T176.2.2.4_femur ............................................................................................................

T176.2.2.5_femur ............................................................................................................

T176.1.1.1_femur CGCTATGTATTTCGTACATTACTGC.........................................................................................................................................................CCTCACCCACTAGGATACCA

T176.1.1.2_femur .........................................................................................................................................................

T176.1.1.3_femur ......................A..................................................................................................................................

T176.1.1.4_femur .........................................................................................................................................................

T176.1.1.5_femur .........................................................................................................................................................

T176.1.2.1_femur .........................................................................................................................................................

T176.1.2.2_femur ............................................................................................................................................T............

T176.1.2.3_femur .........................................................................................................................................................

T176.1.2.4_femur .........................................................................................................................................................

T176.1.2.5_femur .........................................................................................................................................................

T176.2.1.1_femur .........................................................................................................................................................

T176.2.1.2_femur .........................................................................................................................................................

T176.2.1.3_femur .........................................................................................................................................................

T176.2.1.4_femur .........................................................................................................................................................

T176.2.1.5_femur ..................................T......................................................................................................................

T176.2.2.1_femur .........................................................................................................................................................

T176.2.2.2_femur .........................................................................................................................................................

T176.2.2.3_femur .........................................................................................................................................................

T176.2.2.4_femur .........................................................................................................................................................

T176.2.2.5_femur .........................................................................................................................................................

T176.1.1.1_femur CAACTATCACACATCAACTGCAA............................................................................................................C............................

T176.1.1.2_femur ............................................................................................................C............................

T176.1.1.3_femur ............................................................................................................C............................

T176.1.1.4_femur ............................................................................................................C............................

T176.1.1.5_femur ............................................................................................................C............................

T176.1.2.1_femur ............................................................................................................C............................

T176.1.2.2_femur ............................................................................................................C............................

T176.1.2.3_femur ............................................................................................................C............................

T176.1.2.4_femur .........................A..................................................................................C............................

T176.1.2.5_femur ............................................................................................................C............................

T176.2.1.1_femur ............................................................................................................C............................

T176.2.1.2_femur ............................................................................................................C............................

T176.2.1.3_femur ............................................................................................................C............................

T176.2.1.4_femur ............................................................................................................C............................

T176.2.1.5_femur ................................................................................................T...........C............................

T176.2.2.1_femur ............................................................................................................C............................

T176.2.2.2_femur ............................................................................................................C............................

T176.2.2.3_femur ............................................................................................................C............................

T176.2.2.4_femur ............................................................................................................C............................

T176.2.2.5_femur ............................................................................................................C............................

Consensus TTCTTTCATGGGGAAGCAGATTTGGGTACCACCCAAGTATTGACTCACCCATCAACAACCGCTATGTATTTCGTACATTACTGCCAGCCACCATGAATATTGTACGGTACCATAAATACTTGACCACCTGTAGTACATAAAAACCCAATCCACATCAAAACCCCCTCCCCATGCTTACAAGCAAGTACAGCAATCAACCCTCAACTATCACACATCAACTGCAACTCCAAAGCCACCCCTCACCCACTAGGATACCAACAAACCTACCCACCCTTAACAGTACATAGTACATAAAGCCATTTACCGTACATAGCACATTACAGTCAAATCCCCTCTCGTCCCCATGGATGACCCCCCTCAG

1111111111111111111111111111111111111111111111111111111111111111111111111111111111111111111111111111111111111111111111111111111111111111111111111111111111111111111111111111111111111111111111111111111111111111111111111111111111111111111111111111111111111111111111111111111111111111111111111111111111111111111111111111111111111111111111111111111111111111111111111

6666666666666666666666666666666666666666666666666666666666666666666666666666666666666666666666666666666666666666666666666666666666666666666666666666666666666666666666666666666666666666666666666666666666666666666666666666666666666666666666666666666666666666666666666666666666666666666666666666666666666666666666666666666666666666666666666666666666666666666666666

0000000000000000000000000000000000000000000000000000000000000000000000000000111111111111111111111111111111111111111111111111111111111111111111111111111111111111111111111111111122222222222222222222222222222222222222222222222222222222222222222222222222222222222222222222222222223333333333333333333333333333333333333333333333333333333333333333333333333333333333333

2222223333333333444444444455555555556666666666777777777788888888889999999999000000000011111111112222222222333333333344444444445555555555666666666677777777778888888888999999999900000000001111111111222222222233333333334444444444555555555566666666667777777777888888888899999999990000000000111111111122222222223333333333444444444455555555556666666666777777777788888

4567890123456789012345678901234567890123456789012345678901234567890123456789012345678901234567890123456789012345678901234567890123456789012345678901234567890123456789012345678901234567890123456789012345678901234567890123456789012345678901234567890123456789012345678901234567890123456789012345678901234567890123456789012345678901234567890123456789012345678901234

CRS TTCTTTCATGGGGAAGCAGATTTGGGTACCACCCAAGTATTGACTCACCCATCAACAACCGCTATGTATTTCGTACATTACTGCCAGCCACCATGAATATTGTACGGTACCATAAATACTTGACCACCTGTAGTACATAAAAACCCAATCCACATCAAAACCCCCTCCCCATGCTTACAAGCAAGTACAGCAATCAACCCTCAACTATCACACATCAACTGCAACTCCAAAGCCACCCCTCACCCACTAGGATACCAACAAACCTACCCACCCTTAACAGTACATAGTACATAAAGCCATTTACCGTACATAGCACATTACAGTCAAATCCCTTCTCGTCCCCATGGATGACCCCCCTCAG

>T176

T176.1.1.1_ulna ............................................................................................................ACCATAAATACTTGACCACCTGTAGTAC

T176.1.1.2_ulna ............................................................................................................

T176.1.1.3_ulna ............................................................................................................

T176.1.1.4_ulna ............................................................................................................

T176.1.1.5_ulna ............................................................................................................

T176.1.2.1_ulna ............................................................................................................

T176.1.2.2_ulna ................T...........................................................................................

T176.1.2.3_ulna ............................................................................................................

T176.1.2.4_ulna ............................................................................................................

T176.1.2.5_ulna ............................................................................................................

T176.2.1.1_ulna ............................................................................................................

T176.2.1.2_ulna ............................................................................................................

T176.2.1.3_ulna ............................................................................................................

T176.2.1.4_ulna ............................................................................................................

T176.2.1.5_ulna ............................................................................................................

T176.2.2.1_ulna ............................................................................................................

T176.2.2.2_ulna ............................................................................................................

T176.2.2.3_ulna ............................................................................................................

T176.2.2.4_ulna ....................................A.......................................................................

T176.2.2.5_ulna ............................................................................................................

T176.1.1.1_ulna CGCTATGTATTTCGTACATTACTGC.........................................................................................................................................................CCTCACCCACTAGGATACCA

T176.1.1.2_ulna .........................................................................................................................................................

T176.1.1.3_ulna .........................................................................................................................................................

T176.1.1.4_ulna .........................................................................................................................................................

T176.1.1.5_ulna .........................................................................................................................................................

T176.1.2.1_ulna ..................................T......................................................................................................................

T176.1.2.2_ulna .........................................................................................................................................................

T176.1.2.3_ulna .........................................................................................................................................................

T176.1.2.4_ulna .........................................................................................................................................................

T176.1.2.5_ulna .........................................................................................................................................................

T176.2.1.1_ulna .........................................................................................................................................................

T176.2.1.2_ulna .........................................................................................................................................................

T176.2.1.3_ulna .........................................................................................................................................................

T176.2.1.4_ulna .........................................................................................................................................................

T176.2.1.5_ulna .........................................................................................................................................................

T176.2.2.1_ulna .........................................................................................................................................................

T176.2.2.2_ulna .........................................................................................................................................................

T176.2.2.3_ulna .....................................A...................................................................................................................

T176.2.2.4_ulna .........................................................................................................................................................

T176.2.2.5_ulna .........................................................................................................................................................

T176.1.1.1_ulna CAACTATCACACATCAACTGCAA............................................................................................................C............................

T176.1.1.2_ulna ............................................................................................................C............................

T176.1.1.3_ulna ............................................................................................................C............................

T176.1.1.4_ulna ............................................................................................................C............................

T176.1.1.5_ulna ......................T.....................................................................................C............................

T176.1.2.1_ulna ............................................................................................................C............................

T176.1.2.2_ulna ............................................................................................................C............................

T176.1.2.3_ulna ............................................................................................................C............................

T176.1.2.4_ulna ............................................................................................................C............................

T176.1.2.5_ulna ............................................................................................................C............................

T176.2.1.1_ulna ............................................................................................................C............................

T176.2.1.2_ulna ............................................................................................................C............................

T176.2.1.3_ulna ............................................................................................................C............................

T176.2.1.4_ulna ............................................................................................................C............................

T176.2.1.5_ulna ............................................................................................................C............................

T176.2.2.1_ulna ............................................................................................................C............................

T176.2.2.2_ulna ........................................................................................A...................C............................

T176.2.2.3_ulna ............................................................................................................C............................

T176.2.2.4_ulna ............................................................................................................C............................

T176.2.2.5_ulna ............................................................................................................C............................

Consensus TTCTTTCATGGGGAAGCAGATTTGGGTACCACCCAAGTATTGACTCACCCATCAACAACCGCTATGTATTTCGTACATTACTGCCAGCCACCATGAATATTGTACGGTACCATAAATACTTGACCACCTGTAGTACATAAAAACCCAATCCACATCAAAACCCCCTCCCCATGCTTACAAGCAAGTACAGCAATCAACCCTCAACTATCACACATCAACTGCAACTCCAAAGCCACCCCTCACCCACTAGGATACCAACAAACCTACCCACCCTTAACAGTACATAGTACATAAAGCCATTTACCGTACATAGCACATTACAGTCAAATCCCCTCTCGTCCCCATGGATGACCCCCCTCAG

1111111111111111111111111111111111111111111111111111111111111111111111111111111111111111111111111111111111111111111111111111111111111111111111111111111111111111111111111111111111111111111111111111111111111111111111111111111111111111111111111111111111111111111111111111111111111111111111111111111111111111111111111111111111111111111111111111111111111111111111111

6666666666666666666666666666666666666666666666666666666666666666666666666666666666666666666666666666666666666666666666666666666666666666666666666666666666666666666666666666666666666666666666666666666666666666666666666666666666666666666666666666666666666666666666666666666666666666666666666666666666666666666666666666666666666666666666666666666666666666666666666

0000000000000000000000000000000000000000000000000000000000000000000000000000111111111111111111111111111111111111111111111111111111111111111111111111111111111111111111111111111122222222222222222222222222222222222222222222222222222222222222222222222222222222222222222222222222223333333333333333333333333333333333333333333333333333333333333333333333333333333333333

2222223333333333444444444455555555556666666666777777777788888888889999999999000000000011111111112222222222333333333344444444445555555555666666666677777777778888888888999999999900000000001111111111222222222233333333334444444444555555555566666666667777777777888888888899999999990000000000111111111122222222223333333333444444444455555555556666666666777777777788888

4567890123456789012345678901234567890123456789012345678901234567890123456789012345678901234567890123456789012345678901234567890123456789012345678901234567890123456789012345678901234567890123456789012345678901234567890123456789012345678901234567890123456789012345678901234567890123456789012345678901234567890123456789012345678901234567890123456789012345678901234

CRS TTCTTTCATGGGGAAGCAGATTTGGGTACCACCCAAGTATTGACTCACCCATCAACAACCGCTATGTATTTCGTACATTACTGCCAGCCACCATGAATATTGTACGGTACCATAAATACTTGACCACCTGTAGTACATAAAAACCCAATCCACATCAAAACCCCCTCCCCATGCTTACAAGCAAGTACAGCAATCAACCCTCAACTATCACACATCAACTGCAACTCCAAAGCCACCCCTCACCCACTAGGATACCAACAAACCTACCCACCCTTAACAGTACATAGTACATAAAGCCATTTACCGTACATAGCACATTACAGTCAAATCCCTTCTCGTCCCCATGGATGACCCCCCTCAG

>T189

T189.1.1.1_tooth ............................................................................................................ACCATAAATACTTGACCACCTGTAGTAC

T189.1.1.2_tooth ............................................................................................................

T189.1.1.3_tooth ...........................................T................................................................

T189.1.1.4_tooth ............................................................................................................

T189.1.1.5_tooth ............................................................................................................

T189.1.2.1_tooth ............................................................................................................

T189.1.2.2_tooth ............................................................................................................

T189.1.2.3_tooth ............................................................................................................

T189.1.2.4_tooth ............................................................................................................

T189.1.2.5_tooth ............................................................................................................

T189.2.1.1_tooth ............................................................................................................

T189.2.1.2_tooth ............................................................................................................

T189.2.1.3_tooth ............................................................................................................

T189.2.1.4_tooth ............................................................................................................

T189.2.1.5_tooth ............................................................................................................

T189.2.2.1_tooth ............................................................................................................

T189.2.2.2_tooth ............................................................................................................

T189.2.2.3_tooth ............................................................................................................

T189.2.2.4_tooth ............................................................................................................

T189.2.2.5_tooth ............................................................................................................

T189.1.1.1_tooth CGCTATGTATTTCGTACATTACTGC.........................................................................................................................................................CCTCACCCACTAGGATACCA

T189.1.1.2_tooth .........................................................................................................................................................

T189.1.1.3_tooth .........................................................................................................................................................

T189.1.1.4_tooth .........................................................................................................................................................

T189.1.1.5_tooth .........................................................................................................................................................

T189.1.2.1_tooth .........................................................................................................................................................

T189.1.2.2_tooth ...................................................................................................................................................A.....

T189.1.2.3_tooth .........................................................................................................................................................

T189.1.2.4_tooth .........................................................................................................................................................

T189.1.2.5_tooth .........................................................................................................................................................

T189.2.1.1_tooth .........................................................................................................................................................

T189.2.1.2_tooth .........................................................................................................................................................

T189.2.1.3_tooth .........................................................................................................................................................

T189.2.1.4_tooth .........................................................................................................................................................

T189.2.1.5_tooth .........................................................................................................................................................

T189.2.2.1_tooth .........................................................................................................................................................

T189.2.2.2_tooth .........................................................................................................................................................

T189.2.2.3_tooth .........................................................................................................................................................

T189.2.2.4_tooth .........................................................................................................................................................

T189.2.2.5_tooth .........................................................................................................................................................

T189.1.1.1_tooth CAACTATCACACATCAACTGCAA.........................................................................................................................................

T189.1.1.2_tooth .........................................................................................................................................

T189.1.1.3_tooth .........................................................................................................................................

T189.1.1.4_tooth .........................................................................................................................................

T189.1.1.5_tooth .........................................................................................................................................

T189.1.2.1_tooth .........................................................................................................................................

T189.1.2.2_tooth .........................................................................................................................................

T189.1.2.3_tooth .........................................................................................................................................

T189.1.2.4_tooth .........................................................................................................................................

T189.1.2.5_tooth .........................................................................................................................................

T189.2.1.1_tooth .........................................................................................................................................

T189.2.1.2_tooth .........................................................................................................................................

T189.2.1.3_tooth .........................................................................................................................................

T189.2.1.4_tooth .........................................................................................................................................

T189.2.1.5_tooth .........................................................................................................................................

T189.2.2.1_tooth .........................................................................................................................................

T189.2.2.2_tooth .........................................................................................................................................

T189.2.2.3_tooth .........................................................................................................................................

T189.2.2.4_tooth .........................................................................................................................................

T189.2.2.5_tooth .........................................................................................................................................

Consensus TTCTTTCATGGGGAAGCAGATTTGGGTACCACCCAAGTATTGACTCACCCATCAACAACCGCTATGTATTTCGTACATTACTGCCAGCCACCATGAATATTGTACGGTACCATAAATACTTGACCACCTGTAGTACATAAAAACCCAATCCACATCAAAACCCCCTCCCCATGCTTACAAGCAAGTACAGCAATCAACCCTCAACTATCACACATCAACTGCAACTCCAAAGCCACCCCTCACCCACTAGGATACCAACAAACCTACCCACCCTTAACAGTACATAGTACATAAAGCCATTTACCGTACATAGCACATTACAGTCAAATCCCTTCTCGTCCCCATGGATGACCCCCCTCAG

1111111111111111111111111111111111111111111111111111111111111111111111111111111111111111111111111111111111111111111111111111111111111111111111111111111111111111111111111111111111111111111111111111111111111111111111111111111111111111111111111111111111111111111111111111111111111111111111111111111111111111111111111111111111111111111111111111111111111111111111111

6666666666666666666666666666666666666666666666666666666666666666666666666666666666666666666666666666666666666666666666666666666666666666666666666666666666666666666666666666666666666666666666666666666666666666666666666666666666666666666666666666666666666666666666666666666666666666666666666666666666666666666666666666666666666666666666666666666666666666666666666

0000000000000000000000000000000000000000000000000000000000000000000000000000111111111111111111111111111111111111111111111111111111111111111111111111111111111111111111111111111122222222222222222222222222222222222222222222222222222222222222222222222222222222222222222222222222223333333333333333333333333333333333333333333333333333333333333333333333333333333333333

2222223333333333444444444455555555556666666666777777777788888888889999999999000000000011111111112222222222333333333344444444445555555555666666666677777777778888888888999999999900000000001111111111222222222233333333334444444444555555555566666666667777777777888888888899999999990000000000111111111122222222223333333333444444444455555555556666666666777777777788888

4567890123456789012345678901234567890123456789012345678901234567890123456789012345678901234567890123456789012345678901234567890123456789012345678901234567890123456789012345678901234567890123456789012345678901234567890123456789012345678901234567890123456789012345678901234567890123456789012345678901234567890123456789012345678901234567890123456789012345678901234

CRS TTCTTTCATGGGGAAGCAGATTTGGGTACCACCCAAGTATTGACTCACCCATCAACAACCGCTATGTATTTCGTACATTACTGCCAGCCACCATGAATATTGTACGGTACCATAAATACTTGACCACCTGTAGTACATAAAAACCCAATCCACATCAAAACCCCCTCCCCATGCTTACAAGCAAGTACAGCAATCAACCCTCAACTATCACACATCAACTGCAACTCCAAAGCCACCCCTCACCCACTAGGATACCAACAAACCTACCCACCCTTAACAGTACATAGTACATAAAGCCATTTACCGTACATAGCACATTACAGTCAAATCCCTTCTCGTCCCCATGGATGACCCCCCTCAG

>T189

T189.1.1.1_rib ............................................................................................................ACCATAAATACTTGACCACCTGTAGTAC

T189.1.1.2_rib ............................................................................................................

T189.1.1.3_rib ............................................................................................................

T189.1.1.4_rib ............................................................................................................

T189.1.1.5_rib ....................................A.......................................................................

T189.1.2.1_rib ............................................................................................................

T189.1.2.2_rib ............................................................................................................

T189.1.2.3_rib ............................................................................................................

T189.1.2.4_rib ............................................................................................................

T189.1.2.5_rib ............................................................................................................

T189.2.1.1_rib ............................................................................................................

T189.2.1.2_rib ............................................................................................................

T189.2.1.3_rib ............................................................................................................

T189.2.1.4_rib ............................................................................................................

T189.2.1.5_rib ............................................................................................................

T189.2.2.1_rib ............................................................................................................

T189.2.2.2_rib ............................................................................................................

T189.2.2.3_rib ............................................................................................................

T189.2.2.4_rib .....................................................................................................A......

T189.2.2.5_rib ............................................................................................................

T189.1.1.1_rib CGCTATGTATTTCGTACATTACTGC.........................................................................................................................................................CCTCACCCACTAGGATACCA

T189.1.1.2_rib .........................................................................................................................................................

T189.1.1.3_rib .........................................................................................................................................................

T189.1.1.4_rib .........................................................................................................................................................

T189.1.1.5_rib .........................................................................................................................................................

T189.1.2.1_rib .........................................................................................................................................................

T189.1.2.2_rib .........................................................................................................................................................

T189.1.2.3_rib .........................................................................................................................................................

T189.1.2.4_rib .........................................................................................................................................................

T189.1.2.5_rib .........................................................................................................................................................

T189.2.1.1_rib .........................................................................................................................................................

T189.2.1.2_rib .........................................................................................................................................................

T189.2.1.3_rib .........................................................................................................................................................

T189.2.1.4_rib .........................................................................................................................................................

T189.2.1.5_rib .........................................................................................................................................................

T189.2.2.1_rib .........................................................................................................................................................

T189.2.2.2_rib .........................................................................................................................................................

T189.2.2.3_rib .........................................................................................................................................................

T189.2.2.4_rib .........................................................................................................................................................

T189.2.2.5_rib .........................................................................................................................................................

T189.1.1.1_rib CAACTATCACACATCAACTGCAA.........................................................................................................................................

T189.1.1.2_rib .........................................................................................................................................

T189.1.1.3_rib .........................................................................................................................................

T189.1.1.4_rib .........................................................................................................................................

T189.1.1.5_rib .........................................................................................................................................

T189.1.2.1_rib .........................................................................................................................................

T189.1.2.2_rib .........................................................................................................................................

T189.1.2.3_rib .........................................................................................................................................

T189.1.2.4_rib .........................................................................................................................................

T189.1.2.5_rib .........................................................................................................................................

T189.2.1.1_rib .........................................................................................................................................

T189.2.1.2_rib ................................................................................................T........................................

T189.2.1.3_rib .........................................................................................................................................

T189.2.1.4_rib .........................................................................................................................................

T189.2.1.5_rib .........................................................................................................................................

T189.2.2.1_rib .........................................................................................................................................

T189.2.2.2_rib .........................................................................................................................................

T189.2.2.3_rib .........................................................................................................................................

T189.2.2.4_rib .........................................................................................................................................

T189.2.2.5_rib .........................................................................................................................................

Consensus TTCTTTCATGGGGAAGCAGATTTGGGTACCACCCAAGTATTGACTCACCCATCAACAACCGCTATGTATTTCGTACATTACTGCCAGCCACCATGAATATTGTACGGTACCATAAATACTTGACCACCTGTAGTACATAAAAACCCAATCCACATCAAAACCCCCTCCCCATGCTTACAAGCAAGTACAGCAATCAACCCTCAACTATCACACATCAACTGCAACTCCAAAGCCACCCCTCACCCACTAGGATACCAACAAACCTACCCACCCTTAACAGTACATAGTACATAAAGCCATTTACCGTACATAGCACATTACAGTCAAATCCCTTCTCGTCCCCATGGATGACCCCCCTCAG

1111111111111111111111111111111111111111111111111111111111111111111111111111111111111111111111111111111111111111111111111111111111111111111111111111111111111111111111111111111111111111111111111111111111111111111111111111111111111111111111111111111111111111111111111111111111111111111111111111111111111111111111111111111111111111111111111111111111111111111111111

6666666666666666666666666666666666666666666666666666666666666666666666666666666666666666666666666666666666666666666666666666666666666666666666666666666666666666666666666666666666666666666666666666666666666666666666666666666666666666666666666666666666666666666666666666666666666666666666666666666666666666666666666666666666666666666666666666666666666666666666666

0000000000000000000000000000000000000000000000000000000000000000000000000000111111111111111111111111111111111111111111111111111111111111111111111111111111111111111111111111111122222222222222222222222222222222222222222222222222222222222222222222222222222222222222222222222222223333333333333333333333333333333333333333333333333333333333333333333333333333333333333

2222223333333333444444444455555555556666666666777777777788888888889999999999000000000011111111112222222222333333333344444444445555555555666666666677777777778888888888999999999900000000001111111111222222222233333333334444444444555555555566666666667777777777888888888899999999990000000000111111111122222222223333333333444444444455555555556666666666777777777788888

4567890123456789012345678901234567890123456789012345678901234567890123456789012345678901234567890123456789012345678901234567890123456789012345678901234567890123456789012345678901234567890123456789012345678901234567890123456789012345678901234567890123456789012345678901234567890123456789012345678901234567890123456789012345678901234567890123456789012345678901234

CRS TTCTTTCATGGGGAAGCAGATTTGGGTACCACCCAAGTATTGACTCACCCATCAACAACCGCTATGTATTTCGTACATTACTGCCAGCCACCATGAATATTGTACGGTACCATAAATACTTGACCACCTGTAGTACATAAAAACCCAATCCACATCAAAACCCCCTCCCCATGCTTACAAGCAAGTACAGCAATCAACCCTCAACTATCACACATCAACTGCAACTCCAAAGCCACCCCTCACCCACTAGGATACCAACAAACCTACCCACCCTTAACAGTACATAGTACATAAAGCCATTTACCGTACATAGCACATTACAGTCAAATCCCTTCTCGTCCCCATGGATGACCCCCCTCAG

>T189

T189.1.1.1_femur ............................................................................................................ACCATAAATACTTGACCACCTGTAGTAC

T189.1.1.2_femur ............................................................................................................

T189.1.1.3_femur ............................................................................................................

T189.1.1.4_femur ............................................................................................................

T189.1.1.5_femur ............................................................................................................

T189.1.2.1_femur ............................................................................................................

T189.1.2.2_femur ............................................................................................................

T189.1.2.3_femur ............................................................................................................

T189.1.2.4_femur ............................................................................................................

T189.1.2.5_femur ............................................................................................................

T189.2.1.1_femur ............................................................................................................

T189.2.1.2_femur ............................................................................................................

T189.2.1.3_femur ............................................................................................................

T189.2.1.4_femur ............................................................................................................

T189.2.1.5_femur ...............A............................................................................................

T189.2.2.1_femur ............................................................................................................

T189.2.2.2_femur ............................................................................................................

T189.2.2.3_femur ............................................................................................................

T189.2.2.4_femur ............................................................................................................

T189.2.2.5_femur ............................................................................................................

T189.1.1.1_femur CGCTATGTATTTCGTACATTACTGC.........................................................................................................................................................CCTCACCCACTAGGATACCA

T189.1.1.2_femur .........................................................................................................................................................

T189.1.1.3_femur .........................................................................................................................................................

T189.1.1.4_femur .........................................................................................................................................................

T189.1.1.5_femur .........................................................................................................................................................

T189.1.2.1_femur .........................................................................................................................................................

T189.1.2.2_femur .........................................................................................................................................................

T189.1.2.3_femur .........................................................................................................................................................

T189.1.2.4_femur .........................................................................................................................................................

T189.1.2.5_femur .........................................................................................................................................................

T189.2.1.1_femur .........................................................................................................................................................

T189.2.1.2_femur .........................................................................................................................................................

T189.2.1.3_femur .........................................................................................................................................T...............

T189.2.1.4_femur .........................................................................................................................................................

T189.2.1.5_femur .........................................................................................................................................................

T189.2.2.1_femur .........................................................................................................................................................

T189.2.2.2_femur .........................................................................................................................................................

T189.2.2.3_femur .........................................................................................................................................................

T189.2.2.4_femur .........................................................................................................................................................

T189.2.2.5_femur .........................................................................................................................................................

T189.1.1.1_femur CAACTATCACACATCAACTGCAA.........................................................................................................................................

T189.1.1.2_femur .........................................................................................................................................

T189.1.1.3_femur .........................................................................................................................................

T189.1.1.4_femur .........................................................................................................................................

T189.1.1.5_femur .........................................................................................................................................

T189.1.2.1_femur .........................................................................................................................................

T189.1.2.2_femur .........................................................................................................................................

T189.1.2.3_femur .........................................................................................................................................

T189.1.2.4_femur .........................................................................................................................................

T189.1.2.5_femur .........................................................................................................................................

T189.2.1.1_femur .........................................................................................................................................

T189.2.1.2_femur .........................................................................................................................................

T189.2.1.3_femur .........................................................................................................................................

T189.2.1.4_femur .........................................................................................................................................

T189.2.1.5_femur .........................................................................................................................................

T189.2.2.1_femur .........................................................................................................................................

T189.2.2.2_femur .........................................................................................................................................

T189.2.2.3_femur .........................................................................................................................................

T189.2.2.4_femur .........................................................................................................................................

T189.2.2.5_femur .........................................................................................................................................

Consensus TTCTTTCATGGGGAAGCAGATTTGGGTACCACCCAAGTATTGACTCACCCATCAACAACCGCTATGTATTTCGTACATTACTGCCAGCCACCATGAATATTGTACGGTACCATAAATACTTGACCACCTGTAGTACATAAAAACCCAATCCACATCAAAACCCCCTCCCCATGCTTACAAGCAAGTACAGCAATCAACCCTCAACTATCACACATCAACTGCAACTCCAAAGCCACCCCTCACCCACTAGGATACCAACAAACCTACCCACCCTTAACAGTACATAGTACATAAAGCCATTTACCGTACATAGCACATTACAGTCAAATCCCTTCTCGTCCCCATGGATGACCCCCCTCAG

1111111111111111111111111111111111111111111111111111111111111111111111111111111111111111111111111111111111111111111111111111111111111111111111111111111111111111111111111111111111111111111111111111111111111111111111111111111111111111111111111111111111111111111111111111111111111111111111111111111111111111111111111111111111111111111111111111111111111111111111111

6666666666666666666666666666666666666666666666666666666666666666666666666666666666666666666666666666666666666666666666666666666666666666666666666666666666666666666666666666666666666666666666666666666666666666666666666666666666666666666666666666666666666666666666666666666666666666666666666666666666666666666666666666666666666666666666666666666666666666666666666

0000000000000000000000000000000000000000000000000000000000000000000000000000111111111111111111111111111111111111111111111111111111111111111111111111111111111111111111111111111122222222222222222222222222222222222222222222222222222222222222222222222222222222222222222222222222223333333333333333333333333333333333333333333333333333333333333333333333333333333333333

2222223333333333444444444455555555556666666666777777777788888888889999999999000000000011111111112222222222333333333344444444445555555555666666666677777777778888888888999999999900000000001111111111222222222233333333334444444444555555555566666666667777777777888888888899999999990000000000111111111122222222223333333333444444444455555555556666666666777777777788888

4567890123456789012345678901234567890123456789012345678901234567890123456789012345678901234567890123456789012345678901234567890123456789012345678901234567890123456789012345678901234567890123456789012345678901234567890123456789012345678901234567890123456789012345678901234567890123456789012345678901234567890123456789012345678901234567890123456789012345678901234

CRS TTCTTTCATGGGGAAGCAGATTTGGGTACCACCCAAGTATTGACTCACCCATCAACAACCGCTATGTATTTCGTACATTACTGCCAGCCACCATGAATATTGTACGGTACCATAAATACTTGACCACCTGTAGTACATAAAAACCCAATCCACATCAAAACCCCCTCCCCATGCTTACAAGCAAGTACAGCAATCAACCCTCAACTATCACACATCAACTGCAACTCCAAAGCCACCCCTCACCCACTAGGATACCAACAAACCTACCCACCCTTAACAGTACATAGTACATAAAGCCATTTACCGTACATAGCACATTACAGTCAAATCCCTTCTCGTCCCCATGGATGACCCCCCTCAG

>T189

T189.1.1.1_ulna ............................................................................................................ACCATAAATACTTGACCACCTGTAGTAC

T189.1.1.2_ulna ............................................................................................................

T189.1.1.3_ulna ............................................................................................................

T189.1.1.4_ulna ............................................................................................................

T189.1.1.5_ulna ............................................................................................................

T189.1.2.1_ulna ............................................................................................................

T189.1.2.2_ulna ............................................................................................................

T189.1.2.3_ulna ............................................................................................................

T189.1.2.4_ulna ............................................................................................................

T189.1.2.5_ulna ............................................................................................................

T189.2.1.1_ulna ............................................................................................................

T189.2.1.2_ulna ............................................................................................................

T189.2.1.3_ulna ............................................................................................................

T189.2.1.4_ulna ............................................................................................................

T189.2.1.5_ulna ............................................................................................................

T189.2.2.1_ulna ............................................................................................................

T189.2.2.2_ulna ............................................................................................................

T189.2.2.3_ulna ............................................................................................................

T189.2.2.4_ulna ............................................................................................................

T189.2.2.5_ulna ............................................................................................................

T189.1.1.1_ulna CGCTATGTATTTCGTACATTACTGC.........................................................................................................................................................CCTCACCCACTAGGATACCA

T189.1.1.2_ulna .........................................................................................................................................................

T189.1.1.3_ulna .........................................................................................................................................................

T189.1.1.4_ulna .........................................................................................................................................................

T189.1.1.5_ulna .........................................................................................................................................................

T189.1.2.1_ulna .........................................................................................................................................................

T189.1.2.2_ulna .........................................................................................................................................................

T189.1.2.3_ulna .........................................................................................................................................................

T189.1.2.4_ulna .........................................................................................................................................................

T189.1.2.5_ulna .....................................A...................................................................................................................

T189.2.1.1_ulna .........................................................................................................................................................

T189.2.1.2_ulna .........................................................................................................................................................

T189.2.1.3_ulna .........................................................................................................................................................

T189.2.1.4_ulna .........................................................................................................................................................

T189.2.1.5_ulna .........................................................................................................................................................

T189.2.2.1_ulna .........................................................................................................................................................

T189.2.2.2_ulna .........................................................................................................................................................

T189.2.2.3_ulna .........................................................................................................................................................

T189.2.2.4_ulna .........................................................................................................................................................

T189.2.2.5_ulna .........................................................................................................................................................

T189.1.1.1_ulna CAACTATCACACATCAACTGCAA.........................................................................................................................................

T189.1.1.2_ulna .........................................................................................................................................

T189.1.1.3_ulna .........................................................................................................................................

T189.1.1.4_ulna .........................................................................................................................................

T189.1.1.5_ulna .........................................................................................................................................

T189.1.2.1_ulna .........................................................................................................................................

T189.1.2.2_ulna .........................................................................................................................................

T189.1.2.3_ulna .........................................................................................................................................

T189.1.2.4_ulna .........................................................................................................................................

T189.1.2.5_ulna .........................................................................................................................................

T189.2.1.1_ulna .........................................................................................................................................

T189.2.1.2_ulna .........................................................................................................................................

T189.2.1.3_ulna .........................................................................................................................................

T189.2.1.4_ulna ........T................................................................................................................................

T189.2.1.5_ulna .........................................................................................................................................

T189.2.2.1_ulna .........................................................................................................................................

T189.2.2.2_ulna .........................................................................................................................................

T189.2.2.3_ulna .........................................................................................................................................

T189.2.2.4_ulna .........................................................................................................................................

T189.2.2.5_ulna .........................................................................................................................................

Consensus TTCTTTCATGGGGAAGCAGATTTGGGTACCACCCAAGTATTGACTCACCCATCAACAACCGCTATGTATTTCGTACATTACTGCCAGCCACCATGAATATTGTACGGTACCATAAATACTTGACCACCTGTAGTACATAAAAACCCAATCCACATCAAAACCCCCTCCCCATGCTTACAAGCAAGTACAGCAATCAACCCTCAACTATCACACATCAACTGCAACTCCAAAGCCACCCCTCACCCACTAGGATACCAACAAACCTACCCACCCTTAACAGTACATAGTACATAAAGCCATTTACCGTACATAGCACATTACAGTCAAATCCCTTCTCGTCCCCATGGATGACCCCCCTCAG
