# Supplementary material for: Monitoring DNA Contamination in Handled vs. Directly Excavated Ancient Human Skeletal Remains
Source: PLoS One. 2013 Jan 25;8(1):e52524. doi: 10.1371/journal.pone.0052524 (PMC3556025; doi:10.1371/journal.pone.0052524)
Supplement: Dataset S2 — DNA sequences of clones analysed respectively in “Virgin samples” set and “Lab samples” set. The first lines report the Cambridge Reference Sequence (CRS) with the numbering of the nucleotide positions. In the clones sequences nucleotides identical to CRS are indicated by dots. Clones are identified by sample name and 3 digits indicating respectively the number of extraction, the number of PCR and the number of clone. Also the. (DOC) [file pone.0052524.s002.doc]

"Lab samples" set

1111111111111111111111111111111111111111111111111111111111111111111111111111111111111111111111111111111111111111111111111111111111111111111111111111111111111111111111111111111111111111111111111111111111111111111111111111111111111111111111111111111111111111111111111111111111111111111111111111111111111111111111111111111111111111111111111111111111111111111111111

6666666666666666666666666666666666666666666666666666666666666666666666666666666666666666666666666666666666666666666666666666666666666666666666666666666666666666666666666666666666666666666666666666666666666666666666666666666666666666666666666666666666666666666666666666666666666666666666666666666666666666666666666666666666666666666666666666666666666666666666666

0000000000000000000000000000000000000000000000000000000000000000000000000000111111111111111111111111111111111111111111111111111111111111111111111111111111111111111111111111111122222222222222222222222222222222222222222222222222222222222222222222222222222222222222222222222222223333333333333333333333333333333333333333333333333333333333333333333333333333333333333

2222223333333333444444444455555555556666666666777777777788888888889999999999000000000011111111112222222222333333333344444444445555555555666666666677777777778888888888999999999900000000001111111111222222222233333333334444444444555555555566666666667777777777888888888899999999990000000000111111111122222222223333333333444444444455555555556666666666777777777788888

4567890123456789012345678901234567890123456789012345678901234567890123456789012345678901234567890123456789012345678901234567890123456789012345678901234567890123456789012345678901234567890123456789012345678901234567890123456789012345678901234567890123456789012345678901234567890123456789012345678901234567890123456789012345678901234567890123456789012345678901234

CRS TTCTTTCATGGGGAAGCAGATTTGGGTACCACCCAAGTATTGACTCACCCATCAACAACCGCTATGTATTTCGTACATTACTGCCAGCCACCATGAATATTGTACGGTACCATAAATACTTGACCACCTGTAGTACATAAAAACCCAATCCACATCAAAACCCCCTCCCCATGCTTACAAGCAAGTACAGCAATCAACCCTCAACTATCACACATCAACTGCAACTCCAAAGCCACCCCTCACCCACTAGGATACCAACAAACCTACCCACCCTTAACAGTACATAGTACATAAAGCCATTTACCGTACATAGCACATTACAGTCAAATCCCTTCTCGTCCCCATGGATGACCCCCCTCAG

>T148

T148.1.1.1_tooth ......................................................................................................C.....ACCATAAATACTTGACCACCTGTAGTAC

T148.1.1.2_tooth ......................................................................................................C.....

T148.1.1.3_tooth ......................................................................................................C.....

T148.1.1.4_tooth ......................................................................................................C.....

T148.1.1.5_tooth ......................................................................................................C.....

T148.1.2.1_tooth ......................................................................................................C.....

T148.1.2.2_tooth ......................................................................................................C.....

T148.1.2.3_tooth ......................................................................................................C.....

T148.1.2.4_tooth ......................................................................................................C.....

T148.1.2.5_tooth ......................................................................................................C.....

T148.2.1.1_tooth ......................................................................................................C.....

T148.2.1.2_tooth ......................................................................................................C.....

T148.2.1.3_tooth ......................................................................................................C.....

T148.2.1.4_tooth ......................................................................................................C.....

T148.2.1.5_tooth ......................................................................................................C.....

T148.2.2.1_tooth ......................................................................................................C.....

T148.2.2.2_tooth ......................................................................................................C.....

T148.2.2.3_tooth ......................................................................................................C.....

T148.2.2.4_tooth ......................................................................................................C.....

T148.2.2.5_tooth ......................................................................................................C.....

T148.1.1.1_tooth CGCTATGTATTTCGTACATTACTGC..................C......................................................................................................................................CCTCACCCACTAGGATACCA

T148.1.1.2_tooth ..................C......................................................................................................................................

T148.1.1.3_tooth ..................C......................................................................................................................................

T148.1.1.4_tooth ..................C.......T..............................................................................................................................

T148.1.1.5_tooth ..................C......................................................................................................................................

T148.1.2.1_tooth ..................C......................................................................................................................................

T148.1.2.2_tooth ..................C......................................................................................................................................

T148.1.2.3_tooth ..................C......................................................................................................................................

T148.1.2.4_tooth ..................C......................................................................................................................................

T148.1.2.5_tooth ..................C......................................................................................................................................

T148.2.1.1_tooth ..................C......................................................................................................................................

T148.2.1.2_tooth ..................C......................................................................................................................................

T148.2.1.3_tooth ..................C......................................................................................................................................

T148.2.1.4_tooth ..................C......................................................................................................................................

T148.2.1.5_tooth ..................C......................................................................................................................................

T148.2.2.1_tooth ..................C......................................................................................................................................

T148.2.2.2_tooth ..................C......................................................................................................................................

T148.2.2.3_tooth ..................C......................................................................................................................................

T148.2.2.4_tooth ..................C......................................................................................................................................

T148.2.2.5_tooth ..................C......................................................................................................................................

T148.1.1.1_tooth CAACTATCACACATCAACTGCAA.........................................................................................................................................

T148.1.1.2_tooth .........................................................................................................................................

T148.1.1.3_tooth .........................................................................................................................................

T148.1.1.4_tooth .........................................................................................................................................

T148.1.1.5_tooth .........................................................................................................................................

T148.1.2.1_tooth .........................................................................................................................................

T148.1.2.2_tooth .........................................................................................................................................

T148.1.2.3_tooth .........................................................................................................................................

T148.1.2.4_tooth .........................................................................................................................................

T148.1.2.5_tooth .........................................................................................................................................

T148.2.1.1_tooth .........................................................................................................................................

T148.2.1.2_tooth .........................................................................................................................................

T148.2.1.3_tooth .........................................................................................T...............................................

T148.2.1.4_tooth .........................................................................................................................................

T148.2.1.5_tooth .........................................................................................................................................

T148.2.2.1_tooth .........................................................................................................................................

T148.2.2.2_tooth .........................................................................................................................................

T148.2.2.3_tooth .........................................................................................................................................

T148.2.2.4_tooth .........................................................................................................................................

T148.2.2.5_tooth .........................................................................................................................................

consensus TTCTTTCATGGGGAAGCAGATTTGGGTACCACCCAAGTATTGACTCACCCATCAACAACCGCTATGTATTTCGTACATTACTGCCAGCCACCATGAATATTGCACGGTACCATAAATACTTGACCACCTGTAGTACATAAAAACCCAATCCACATCAAAACCCCCTCCCCATGCTTACAAGCAAGTACAGCAATCAACCCTCAACTATCACACATCAACTGCAACTCCAAAGCCACCCCTCACCCACTAGGATACCAACAAACCTACCCACCCTTAACAGTACATAGTACATAAAGCCATTTACCGTACATAGCACATTACAGTCAAATCCCTTCTCGTCCCCATGGATGACCCCCCTCAG

1111111111111111111111111111111111111111111111111111111111111111111111111111111111111111111111111111111111111111111111111111111111111111111111111111111111111111111111111111111111111111111111111111111111111111111111111111111111111111111111111111111111111111111111111111111111111111111111111111111111111111111111111111111111111111111111111111111111111111111111111

6666666666666666666666666666666666666666666666666666666666666666666666666666666666666666666666666666666666666666666666666666666666666666666666666666666666666666666666666666666666666666666666666666666666666666666666666666666666666666666666666666666666666666666666666666666666666666666666666666666666666666666666666666666666666666666666666666666666666666666666666

0000000000000000000000000000000000000000000000000000000000000000000000000000111111111111111111111111111111111111111111111111111111111111111111111111111111111111111111111111111122222222222222222222222222222222222222222222222222222222222222222222222222222222222222222222222222223333333333333333333333333333333333333333333333333333333333333333333333333333333333333

2222223333333333444444444455555555556666666666777777777788888888889999999999000000000011111111112222222222333333333344444444445555555555666666666677777777778888888888999999999900000000001111111111222222222233333333334444444444555555555566666666667777777777888888888899999999990000000000111111111122222222223333333333444444444455555555556666666666777777777788888

4567890123456789012345678901234567890123456789012345678901234567890123456789012345678901234567890123456789012345678901234567890123456789012345678901234567890123456789012345678901234567890123456789012345678901234567890123456789012345678901234567890123456789012345678901234567890123456789012345678901234567890123456789012345678901234567890123456789012345678901234

CRS TTCTTTCATGGGGAAGCAGATTTGGGTACCACCCAAGTATTGACTCACCCATCAACAACCGCTATGTATTTCGTACATTACTGCCAGCCACCATGAATATTGTACGGTACCATAAATACTTGACCACCTGTAGTACATAAAAACCCAATCCACATCAAAACCCCCTCCCCATGCTTACAAGCAAGTACAGCAATCAACCCTCAACTATCACACATCAACTGCAACTCCAAAGCCACCCCTCACCCACTAGGATACCAACAAACCTACCCACCCTTAACAGTACATAGTACATAAAGCCATTTACCGTACATAGCACATTACAGTCAAATCCCTTCTCGTCCCCATGGATGACCCCCCTCAG

>T148

T148.1.1.1_rib ............................................................................................................ACCATAAATACTTGACCACCTGTAGTAC

T148.1.1.2_rib ............................................................................................................

T148.1.1.3_rib ............................................................................................................

T148.1.1.4_rib ............................................................................................................

T148.1.1.5_rib ............................................................................................................

T148.1.2.1_rib ............................................................................................................

T148.1.2.2_rib ............................................................................................................

T148.1.2.3_rib ............................................................................................................

T148.1.2.4_rib ............................................................................................................

T148.1.2.5_rib ............................................................................................................

T148.2.1.1_rib ............................................................................................................

T148.2.1.2_rib ............................................................................................................

T148.2.1.3_rib ............................................................................................................

T148.2.1.4_rib ............................................................................................................

T148.2.1.5_rib ............................................................................................................

T148.2.2.1_rib ............................................................................................................

T148.2.2.2_rib ............................................................................................................

T148.2.2.3_rib ............................................................................................................

T148.2.2.4_rib ............................................................................................................

T148.2.2.5_rib ............................................................................................................

T148.1.1.1_rib CGCTATGTATTTCGTACATTACTGC.........................................................................................................................................................CCTCACCCACTAGGATACCA

T148.1.1.2_rib .........................................................................................................................................................

T148.1.1.3_rib .........................................................................................................................................................

T148.1.1.4_rib .........................................................................................................................................................

T148.1.1.5_rib .........................................................................................................................................................

T148.1.2.1_rib .........................................................................................................................................................

T148.1.2.2_rib .........................................................................................................................................................

T148.1.2.3_rib .........................................................................................................................................................

T148.1.2.4_rib .........................................................................................................................................................

T148.1.2.5_rib .........................................................................................................................................................

T148.2.1.1_rib .........................................................................................................................................................

T148.2.1.2_rib .........................................................................................................................................................

T148.2.1.3_rib .........................................................................................................................................................

T148.2.1.4_rib .........................................................................................................................................................

T148.2.1.5_rib .........................................................................................................................................................

T148.2.2.1_rib .........................................................................................................................................................

T148.2.2.2_rib .........................................................................................................................................................

T148.2.2.3_rib .........................................................................................................................................................

T148.2.2.4_rib .........................................................................................................................................................

T148.2.2.5_rib .........................................................................................................................................................

T148.1.1.1_rib CAACTATCACACATCAACTGCAA.........................................................................................................................................

T148.1.1.2_rib .........................................................................................................................................

T148.1.1.3_rib .........................................................................................................................................

T148.1.1.4_rib .........................................................................................................................................

T148.1.1.5_rib .........................................................................................................................................

T148.1.2.1_rib .........................................................................................................................................

T148.1.2.2_rib .........................................................................................................................................

T148.1.2.3_rib .........................................................................................................................................

T148.1.2.4_rib .........................................................................................................................................

T148.1.2.5_rib .........................................................................................................................................

T148.2.1.1_rib .........................................................................................................................................

T148.2.1.2_rib .........................................................................................................................................

T148.2.1.3_rib .........................................................................................................................................

T148.2.1.4_rib .........................................................................................................................................

T148.2.1.5_rib .........................................................................................................................................

T148.2.2.1_rib .........................................................................................................................................

T148.2.2.2_rib .........................................................................................................................................

T148.2.2.3_rib .........................................................................................................................................

T148.2.2.4_rib .........................................................................................................................................

T148.2.2.5_rib .........................................................................................................................................

Consensus TTCTTTCATGGGGAAGCAGATTTGGGTACCACCCAAGTATTGACTCACCCATCAACAACCGCTATGTATTTCGTACATTACTGCCAGCCACCATGAATATTGTACGGTACCATAAATACTTGACCACCTGTAGTACATAAAAACCCAATCCACATCAAAACCCCCTCCCCATGCTTACAAGCAAGTACAGCAATCAACCCTCAACTATCACACATCAACTGCAACTCCAAAGCCACCCCTCACCCACTAGGATACCAACAAACCTACCCACCCTTAACAGTACATAGTACATAAAGCCATTTACCGTACATAGCACATTACAGTCAAATCCCTTCTCGTCCCCATGGATGACCCCCCTCAG

1111111111111111111111111111111111111111111111111111111111111111111111111111111111111111111111111111111111111111111111111111111111111111111111111111111111111111111111111111111111111111111111111111111111111111111111111111111111111111111111111111111111111111111111111111111111111111111111111111111111111111111111111111111111111111111111111111111111111111111111111

6666666666666666666666666666666666666666666666666666666666666666666666666666666666666666666666666666666666666666666666666666666666666666666666666666666666666666666666666666666666666666666666666666666666666666666666666666666666666666666666666666666666666666666666666666666666666666666666666666666666666666666666666666666666666666666666666666666666666666666666666

0000000000000000000000000000000000000000000000000000000000000000000000000000111111111111111111111111111111111111111111111111111111111111111111111111111111111111111111111111111122222222222222222222222222222222222222222222222222222222222222222222222222222222222222222222222222223333333333333333333333333333333333333333333333333333333333333333333333333333333333333

2222223333333333444444444455555555556666666666777777777788888888889999999999000000000011111111112222222222333333333344444444445555555555666666666677777777778888888888999999999900000000001111111111222222222233333333334444444444555555555566666666667777777777888888888899999999990000000000111111111122222222223333333333444444444455555555556666666666777777777788888

4567890123456789012345678901234567890123456789012345678901234567890123456789012345678901234567890123456789012345678901234567890123456789012345678901234567890123456789012345678901234567890123456789012345678901234567890123456789012345678901234567890123456789012345678901234567890123456789012345678901234567890123456789012345678901234567890123456789012345678901234

CRS TTCTTTCATGGGGAAGCAGATTTGGGTACCACCCAAGTATTGACTCACCCATCAACAACCGCTATGTATTTCGTACATTACTGCCAGCCACCATGAATATTGTACGGTACCATAAATACTTGACCACCTGTAGTACATAAAAACCCAATCCACATCAAAACCCCCTCCCCATGCTTACAAGCAAGTACAGCAATCAACCCTCAACTATCACACATCAACTGCAACTCCAAAGCCACCCCTCACCCACTAGGATACCAACAAACCTACCCACCCTTAACAGTACATAGTACATAAAGCCATTTACCGTACATAGCACATTACAGTCAAATCCCTTCTCGTCCCCATGGATGACCCCCCTCAG

>T148

T148.1.1.1_femur ......................................................................................................C.....ACCATAAATACTTGACCACCTGTAGTAC

T148.1.1.2_femur ......................................................................................................C.....

T148.1.1.3_femur ......................................................................................................C.....

T148.1.1.4_femur ......................................................................................................C.....

T148.1.1.5_femur ......................................................................................................C.....

T148.1.2.1_femur ......................................................................................................C.....

T148.1.2.2_femur ......................................................................................................C.....

T148.1.2.3_femur ......................................................................................................C.....

T148.1.2.4_femur ......................................................................................................C.....

T148.1.2.5_femur ......................................................................................................C.....

T148.2.1.1_femur ......................................................................................................C.....

T148.2.1.2_femur ......................................................................................................C.....

T148.2.1.3_femur ......................................................................................................C.....

T148.2.1.4_femur ......................................................................................................C.....

T148.2.1.5_femur ......................................................................................................C.....

T148.2.2.1_femur ......................................................................................................C.....

T148.2.2.2_femur ......................................................................................................C.....

T148.2.2.3_femur ......................................................................................................C.....

T148.2.2.4_femur ......................................................................................................C.....

T148.2.2.5_femur ......................................................................................................C.....

T148.1.1.1_femur CGCTATGTATTTCGTACATTACTGC..................C......................................................................................................................................CCTCACCCACTAGGATACCA

T148.1.1.2_femur ..................C......................................................................................................................................

T148.1.1.3_femur ..................C......................................................................................................................................

T148.1.1.4_femur ..................C......................................................................................................................................

T148.1.1.5_femur ..................C......................................................................................................................................

T148.1.2.1_femur ..................C......................................................................................................................................

T148.1.2.2_femur ..................C......................................................................................................................................

T148.1.2.3_femur ..................C......................................................................................................................................

T148.1.2.4_femur ..................C......................................................................................................................................

T148.1.2.5_femur ..................C......................................................................................................................................

T148.2.1.1_femur ..................C......................................................................................................................................

T148.2.1.2_femur ..................C......................................................................................................................................

T148.2.1.3_femur ..................C......................................................................................................................................

T148.2.1.4_femur ..................C......................................................................................................................................

T148.2.1.5_femur ..................C......................................................................................................................................

T148.2.2.1_femur ..................C......................................................................................................................................

T148.2.2.2_femur ..................C......................................................................................................................................

T148.2.2.3_femur ..................C......................................................................................................................................

T148.2.2.4_femur ..................C......................................................................................................................................

T148.2.2.5_femur ..................C......................................................................................................................................

T148.1.1.1_femur CAACTATCACACATCAACTGCAA.........................................................................................................................................

T148.1.1.2_femur .........................................................................................................................................

T148.1.1.3_femur .........................................................................................................................................

T148.1.1.4_femur .........................................................................................................................................

T148.1.1.5_femur .........................................................................................................................................

T148.1.2.1_femur .........................................................................................................................................

T148.1.2.2_femur .........................................................................................................................................

T148.1.2.3_femur .........................................................................................................................................

T148.1.2.4_femur .........................................................................................................................................

T148.1.2.5_femur .........................................................................................................................................

T148.2.1.1_femur .........................................................................................................................................

T148.2.1.2_femur .........................................................................................................................................

T148.2.1.3_femur .........................................................................................................................................

T148.2.1.4_femur .........................................................................................................................................

T148.2.1.5_femur .........................................................................................................................................

T148.2.2.1_femur .........................................................................................................................................

T148.2.2.2_femur .........................................................................................................................................

T148.2.2.3_femur .........................................................................................................................................

T148.2.2.4_femur .........................................................................................................................................

T148.2.2.5_femur .........................................................................................................................................

Consensus TTCTTTCATGGGGAAGCAGATTTGGGTACCACCCAAGTATTGACTCACCCATCAACAACCGCTATGTATTTCGTACATTACTGCCAGCCACCATGAATATTGCACGGTACCATAAATACTTGACCACCTGTAGTACATAAAAACCCAATCCACATCAAAACCCCCTCCCCATGCTTACAAGCAAGTACAGCAATCAACCCTCAACTATCACACATCAACTGCAACTCCAAAGCCACCCCTCACCCACTAGGATACCAACAAACCTACCCACCCTTAACAGTACATAGTACATAAAGCCATTTACCGTACATAGCACATTACAGTCAAATCCCTTCTCGTCCCCATGGATGACCCCCCTCAG

1111111111111111111111111111111111111111111111111111111111111111111111111111111111111111111111111111111111111111111111111111111111111111111111111111111111111111111111111111111111111111111111111111111111111111111111111111111111111111111111111111111111111111111111111111111111111111111111111111111111111111111111111111111111111111111111111111111111111111111111111

6666666666666666666666666666666666666666666666666666666666666666666666666666666666666666666666666666666666666666666666666666666666666666666666666666666666666666666666666666666666666666666666666666666666666666666666666666666666666666666666666666666666666666666666666666666666666666666666666666666666666666666666666666666666666666666666666666666666666666666666666

0000000000000000000000000000000000000000000000000000000000000000000000000000111111111111111111111111111111111111111111111111111111111111111111111111111111111111111111111111111122222222222222222222222222222222222222222222222222222222222222222222222222222222222222222222222222223333333333333333333333333333333333333333333333333333333333333333333333333333333333333

2222223333333333444444444455555555556666666666777777777788888888889999999999000000000011111111112222222222333333333344444444445555555555666666666677777777778888888888999999999900000000001111111111222222222233333333334444444444555555555566666666667777777777888888888899999999990000000000111111111122222222223333333333444444444455555555556666666666777777777788888

4567890123456789012345678901234567890123456789012345678901234567890123456789012345678901234567890123456789012345678901234567890123456789012345678901234567890123456789012345678901234567890123456789012345678901234567890123456789012345678901234567890123456789012345678901234567890123456789012345678901234567890123456789012345678901234567890123456789012345678901234

CRS TTCTTTCATGGGGAAGCAGATTTGGGTACCACCCAAGTATTGACTCACCCATCAACAACCGCTATGTATTTCGTACATTACTGCCAGCCACCATGAATATTGTACGGTACCATAAATACTTGACCACCTGTAGTACATAAAAACCCAATCCACATCAAAACCCCCTCCCCATGCTTACAAGCAAGTACAGCAATCAACCCTCAACTATCACACATCAACTGCAACTCCAAAGCCACCCCTCACCCACTAGGATACCAACAAACCTACCCACCCTTAACAGTACATAGTACATAAAGCCATTTACCGTACATAGCACATTACAGTCAAATCCCTTCTCGTCCCCATGGATGACCCCCCTCAG

>T148

T148.1.1.1_ulna ............................................................................................................ACCATAAATACTTGACCACCTGTAGTAC

T148.1.1.2_ulna ............................................................................................................

T148.1.1.3_ulna ............................................................................................................

T148.1.1.4_ulna ............................................................................................................

T148.1.1.5_ulna ............................................................................................................

T148.1.2.1_ulna ............................................................................................................

T148.1.2.2_ulna ............................................................................................................

T148.1.2.3_ulna ............................................................................................................

T148.1.2.4_ulna ............................................................................................................

T148.1.2.5_ulna ............................................................................................................

T148.2.1.1_ulna ............................................................................................................

T148.2.1.2_ulna ............................................................................................................

T148.2.1.3_ulna ............................................................................................................

T148.2.1.4_ulna ............................................................................................................

T148.2.1.5_ulna ............................................................................................................

T148.2.2.1_ulna ............................................................................................................

T148.2.2.2_ulna ............................................................................................................

T148.2.2.3_ulna ............................................................................................................

T148.2.2.4_ulna ............................................................................................................

T148.2.2.5_ulna ............................................................................................................

T148.1.1.1_ulna CGCTATGTATTTCGTACATTACTGC.........................................................................................................................................................CCTCACCCACTAGGATACCA

T148.1.1.2_ulna .........................................................................................................................................................

T148.1.1.3_ulna .........................................................................................................................................................

T148.1.1.4_ulna .........................................................................................................................................................

T148.1.1.5_ulna .........................................................................................................................................................

T148.1.2.1_ulna .........................................................................................................................................................

T148.1.2.2_ulna .........................................................................................................................................................

T148.1.2.3_ulna .........................................................................................................................................................

T148.1.2.4_ulna .........................................................................................................................................................

T148.1.2.5_ulna .........................................................................................................................................................

T148.2.1.1_ulna .........................................................................................................................................................

T148.2.1.2_ulna .........................................................................................................................................................

T148.2.1.3_ulna .........................................................................................................................................................

T148.2.1.4_ulna .........................................................................................................................................................

T148.2.1.5_ulna .........................................................................................................................................................

T148.2.2.1_ulna .........................................................................................................................................................

T148.2.2.2_ulna .........................................................................................................................................................

T148.2.2.3_ulna .........................................................................................................................................................

T148.2.2.4_ulna .........................................................................................................................................................

T148.2.2.5_ulna .........................................................................................................................................................

T148.1.1.1_ulna CAACTATCACACATCAACTGCAA.........................................................................................................................................

T148.1.1.2_ulna .........................................................................................................................................

T148.1.1.3_ulna .........................................................................................................................................

T148.1.1.4_ulna .........................................................................................................................................

T148.1.1.5_ulna .........................................................................................................................................

T148.1.2.1_ulna .........................................................................................................................................

T148.1.2.2_ulna .........................................................................................................................................

T148.1.2.3_ulna .........................................................................................................................................

T148.1.2.4_ulna .........................................................................................................................................

T148.1.2.5_ulna .........................................................................................................................................

T148.2.1.1_ulna .........................................................................................................................................

T148.2.1.2_ulna .........................................................................................................................................

T148.2.1.3_ulna .........................................................................................................................................

T148.2.1.4_ulna .........................................................................................................................................

T148.2.1.5_ulna .........................................................................................................................................

T148.2.2.1_ulna .........................................................................................................................................

T148.2.2.2_ulna .........................................................................................................................................

T148.2.2.3_ulna .........................................................................................................................................

T148.2.2.4_ulna .........................................................................................................................................

T148.2.2.5_ulna .........................................................................................................................................

Consensus TTCTTTCATGGGGAAGCAGATTTGGGTACCACCCAAGTATTGACTCACCCATCAACAACCGCTATGTATTTCGTACATTACTGCCAGCCACCATGAATATTGTACGGTACCATAAATACTTGACCACCTGTAGTACATAAAAACCCAATCCACATCAAAACCCCCTCCCCATGCTTACAAGCAAGTACAGCAATCAACCCTCAACTATCACACATCAACTGCAACTCCAAAGCCACCCCTCACCCACTAGGATACCAACAAACCTACCCACCCTTAACAGTACATAGTACATAAAGCCATTTACCGTACATAGCACATTACAGTCAAATCCCTTCTCGTCCCCATGGATGACCCCCCTCAG

1111111111111111111111111111111111111111111111111111111111111111111111111111111111111111111111111111111111111111111111111111111111111111111111111111111111111111111111111111111111111111111111111111111111111111111111111111111111111111111111111111111111111111111111111111111111111111111111111111111111111111111111111111111111111111111111111111111111111111111111111

6666666666666666666666666666666666666666666666666666666666666666666666666666666666666666666666666666666666666666666666666666666666666666666666666666666666666666666666666666666666666666666666666666666666666666666666666666666666666666666666666666666666666666666666666666666666666666666666666666666666666666666666666666666666666666666666666666666666666666666666666

0000000000000000000000000000000000000000000000000000000000000000000000000000111111111111111111111111111111111111111111111111111111111111111111111111111111111111111111111111111122222222222222222222222222222222222222222222222222222222222222222222222222222222222222222222222222223333333333333333333333333333333333333333333333333333333333333333333333333333333333333

2222223333333333444444444455555555556666666666777777777788888888889999999999000000000011111111112222222222333333333344444444445555555555666666666677777777778888888888999999999900000000001111111111222222222233333333334444444444555555555566666666667777777777888888888899999999990000000000111111111122222222223333333333444444444455555555556666666666777777777788888

4567890123456789012345678901234567890123456789012345678901234567890123456789012345678901234567890123456789012345678901234567890123456789012345678901234567890123456789012345678901234567890123456789012345678901234567890123456789012345678901234567890123456789012345678901234567890123456789012345678901234567890123456789012345678901234567890123456789012345678901234

CRS TTCTTTCATGGGGAAGCAGATTTGGGTACCACCCAAGTATTGACTCACCCATCAACAACCGCTATGTATTTCGTACATTACTGCCAGCCACCATGAATATTGTACGGTACCATAAATACTTGACCACCTGTAGTACATAAAAACCCAATCCACATCAAAACCCCCTCCCCATGCTTACAAGCAAGTACAGCAATCAACCCTCAACTATCACACATCAACTGCAACTCCAAAGCCACCCCTCACCCACTAGGATACCAACAAACCTACCCACCCTTAACAGTACATAGTACATAAAGCCATTTACCGTACATAGCACATTACAGTCAAATCCCTTCTCGTCCCCATGGATGACCCCCCTCAG

>T164

T164.1.1.1_tooth ............................................................................................................ACCATAAATACTTGACCACCTGTAGTAC

T164.1.1.2_tooth ............................................................................................................

T164.1.1.3_tooth .......................................................................................T....................

T164.1.1.4_tooth ............................................................................................................

T164.1.1.5_tooth ............................................................................................................

T164.1.2.1_tooth ............................................................................................................

T164.1.2.2_tooth ............................................................................................................

T164.1.2.3_tooth ............................................................................................................

T164.1.2.4_tooth ............................................................................................................

T164.1.2.5_tooth ............................................................................................................

T164.2.1.1_tooth ............................................................................................................

T164.2.1.2_tooth ............................................................................................................

T164.2.1.3_tooth ............................................................................................................

T164.2.1.4_tooth ............................................................................................................

T164.2.1.5_tooth ............................................................................................................

T164.2.2.1_tooth ............................................................................................................

T164.2.2.2_tooth ............................................................................................................

T164.2.2.3_tooth ............................................................................................................

T164.2.2.4_tooth ............................................................................................................

T164.2.2.5_tooth ............................................................................................................

T164.1.1.1_tooth CGCTATGTATTTCGTACATTACTGC.........................................................................................................................................................CCTCACCCACTAGGATACCA

T164.1.1.2_tooth .........................................................................................................................................................

T164.1.1.3_tooth .........................................................................................................................................................

T164.1.1.4_tooth .........................................................................................................................................................

T164.1.1.5_tooth .........................................................................................................................................................

T164.1.2.1_tooth .........................................................................................................................................................

T164.1.2.2_tooth .........................................................................................................................................................

T164.1.2.3_tooth ..........A..............................................................................................................................................

T164.1.2.4_tooth .........................................................................................................................................................

T164.1.2.5_tooth .........................................................................................................................................................

T164.2.1.1_tooth .........................................................................................................................................................

T164.2.1.2_tooth .........................................................................................................................................................

T164.2.1.3_tooth .........................................................................................................................................................

T164.2.1.4_tooth .........................................................................................................................................................

T164.2.1.5_tooth .........................................................................................................................................................

T164.2.2.1_tooth .........................................................................................................................................................

T164.2.2.2_tooth .........................................................................................................................................................

T164.2.2.3_tooth .........................................................................................................................................................

T164.2.2.4_tooth .........................................................................................................................................................

T164.2.2.5_tooth .........................................................................................................................................................

T164.1.1.1_tooth CAACTATCACACATCAACTGCAA.........................................................................................................................................

T164.1.1.2_tooth .........................................................................................................................................

T164.1.1.3_tooth .........................................................................................................................................

T164.1.1.4_tooth .........................................................................................................................................

T164.1.1.5_tooth .........................................................................................................................................

T164.1.2.1_tooth .........................................................................................................................................

T164.1.2.2_tooth .........................................................................................................................................

T164.1.2.3_tooth .........................................................................................................................................

T164.1.2.4_tooth .........................................................................................................................................

T164.1.2.5_tooth .........................................................................................................................................

T164.2.1.1_tooth .........................................................................................................................................

T164.2.1.2_tooth .........................................................................................................................................

T164.2.1.3_tooth .........................................................................................................................................

T164.2.1.4_tooth .........................................................................................................................................

T164.2.1.5_tooth .........................................................................................................................................

T164.2.2.1_tooth .........................................................................................................................................

T164.2.2.2_tooth .........................................................................................................................................

T164.2.2.3_tooth .........................................................................................................................................

T164.2.2.4_tooth .........................................................................................................................................

T164.2.2.5_tooth .........................................................................................................................................

Consensus TTCTTTCATGGGGAAGCAGATTTGGGTACCACCCAAGTATTGACTCACCCATCAACAACCGCTATGTATTTCGTACATTACTGCCAGCCACCATGAATATTGTACGGTACCATAAATACTTGACCACCTGTAGTACATAAAAACCCAATCCACATCAAAACCCCCTCCCCATGCTTACAAGCAAGTACAGCAATCAACCCTCAACTATCACACATCAACTGCAACTCCAAAGCCACCCCTCACCCACTAGGATACCAACAAACCTACCCACCCTTAACAGTACATAGTACATAAAGCCATTTACCGTACATAGCACATTACAGTCAAATCCCTTCTCGTCCCCATGGATGACCCCCCTCAG

1111111111111111111111111111111111111111111111111111111111111111111111111111111111111111111111111111111111111111111111111111111111111111111111111111111111111111111111111111111111111111111111111111111111111111111111111111111111111111111111111111111111111111111111111111111111111111111111111111111111111111111111111111111111111111111111111111111111111111111111111

6666666666666666666666666666666666666666666666666666666666666666666666666666666666666666666666666666666666666666666666666666666666666666666666666666666666666666666666666666666666666666666666666666666666666666666666666666666666666666666666666666666666666666666666666666666666666666666666666666666666666666666666666666666666666666666666666666666666666666666666666

0000000000000000000000000000000000000000000000000000000000000000000000000000111111111111111111111111111111111111111111111111111111111111111111111111111111111111111111111111111122222222222222222222222222222222222222222222222222222222222222222222222222222222222222222222222222223333333333333333333333333333333333333333333333333333333333333333333333333333333333333

2222223333333333444444444455555555556666666666777777777788888888889999999999000000000011111111112222222222333333333344444444445555555555666666666677777777778888888888999999999900000000001111111111222222222233333333334444444444555555555566666666667777777777888888888899999999990000000000111111111122222222223333333333444444444455555555556666666666777777777788888

4567890123456789012345678901234567890123456789012345678901234567890123456789012345678901234567890123456789012345678901234567890123456789012345678901234567890123456789012345678901234567890123456789012345678901234567890123456789012345678901234567890123456789012345678901234567890123456789012345678901234567890123456789012345678901234567890123456789012345678901234

CRS TTCTTTCATGGGGAAGCAGATTTGGGTACCACCCAAGTATTGACTCACCCATCAACAACCGCTATGTATTTCGTACATTACTGCCAGCCACCATGAATATTGTACGGTACCATAAATACTTGACCACCTGTAGTACATAAAAACCCAATCCACATCAAAACCCCCTCCCCATGCTTACAAGCAAGTACAGCAATCAACCCTCAACTATCACACATCAACTGCAACTCCAAAGCCACCCCTCACCCACTAGGATACCAACAAACCTACCCACCCTTAACAGTACATAGTACATAAAGCCATTTACCGTACATAGCACATTACAGTCAAATCCCTTCTCGTCCCCATGGATGACCCCCCTCAG

>T164

T164.1.1.1_rib ............................................................................................................ACCATAAATACTTGACCACCTGTAGTAC

T164.1.1.2_rib ............................................................................................................

T164.1.1.3_rib ............................................................................................................

T164.1.1.4_rib ............................................................................................................

T164.1.1.5_rib ............................................................................................................

T164.1.2.1_rib ............................................................................................................

T164.1.2.2_rib ............................................................................................................

T164.1.2.3_rib ............................................................................................................

T164.1.2.4_rib ............................................................................................................

T164.1.2.5_rib ............................................................................................................

T164.2.1.1_rib ............................................................................................................

T164.2.1.2_rib ............................................................................................................

T164.2.1.3_rib ............................................................................................................

T164.2.1.4_rib ............................................................................................................

T164.2.1.5_rib ............................................................................................................

T164.2.2.1_rib ............................................................................................................

T164.2.2.2_rib ............................................................................................................

T164.2.2.3_rib ............................................................................................................

T164.2.2.4_rib ............................................................................................................

T164.2.2.5_rib ............................................................................................................

T164.1.1.1_rib CGCTATGTATTTCGTACATTACTGC.........................................................................................................................................................CCTCACCCACTAGGATACCA

T164.1.1.2_rib .........................................................................................................................................................

T164.1.1.3_rib .........................................................................................................................................................

T164.1.1.4_rib .........................................................................................................................................................

T164.1.1.5_rib .........................................................................................................................................................

T164.1.2.1_rib .........................................................................................................................................................

T164.1.2.2_rib .........................................................................................................................................................

T164.1.2.3_rib .........................................................................................................................................................

T164.1.2.4_rib .........................................................................................................................................................

T164.1.2.5_rib .........................................................................................................................................................

T164.2.1.1_rib .........................................................................................................................................................

T164.2.1.2_rib .........................................................................................................................................................

T164.2.1.3_rib .........................................................................................................................................................

T164.2.1.4_rib .........................................................................................................................................................

T164.2.1.5_rib .........................................................................................................................................................

T164.2.2.1_rib .........................................................................................................................................................

T164.2.2.2_rib .........................................................................................................................................................

T164.2.2.3_rib .........................................................................................................................................................

T164.2.2.4_rib .........................................................................................................................................................

T164.2.2.5_rib .........................................................................................................................................................

T164.1.1.1_rib CAACTATCACACATCAACTGCAA.........................................................................................................................................

T164.1.1.2_rib .........................................................................................................................................

T164.1.1.3_rib .........................................................................................................................................

T164.1.1.4_rib .........................................................................................................................................

T164.1.1.5_rib .........................................................................................................................................

T164.1.2.1_rib .........................................................................................................................................

T164.1.2.2_rib .........................................................................................................................................

T164.1.2.3_rib .........................................................................................................................................

T164.1.2.4_rib .........................................................................................................................................

T164.1.2.5_rib .........................................................................................................................................

T164.2.1.1_rib .........................................................................................................................................

T164.2.1.2_rib .........................................................................................................................................

T164.2.1.3_rib .........................................................................................................................................

T164.2.1.4_rib .........................................................................................................................................

T164.2.1.5_rib .........................................................................................................................................

T164.2.2.1_rib .........................................................................................................................................

T164.2.2.2_rib .........................................................................................................................................

T164.2.2.3_rib .........................................................................................................................................

T164.2.2.4_rib .........................................................................................................................................

T164.2.2.5_rib .........................................................................................................................................

Consensus TTCTTTCATGGGGAAGCAGATTTGGGTACCACCCAAGTATTGACTCACCCATCAACAACCGCTATGTATTTCGTACATTACTGCCAGCCACCATGAATATTGTACGGTACCATAAATACTTGACCACCTGTAGTACATAAAAACCCAATCCACATCAAAACCCCCTCCCCATGCTTACAAGCAAGTACAGCAATCAACCCTCAACTATCACACATCAACTGCAACTCCAAAGCCACCCCTCACCCACTAGGATACCAACAAACCTACCCACCCTTAACAGTACATAGTACATAAAGCCATTTACCGTACATAGCACATTACAGTCAAATCCCTTCTCGTCCCCATGGATGACCCCCCTCAG

1111111111111111111111111111111111111111111111111111111111111111111111111111111111111111111111111111111111111111111111111111111111111111111111111111111111111111111111111111111111111111111111111111111111111111111111111111111111111111111111111111111111111111111111111111111111111111111111111111111111111111111111111111111111111111111111111111111111111111111111111

6666666666666666666666666666666666666666666666666666666666666666666666666666666666666666666666666666666666666666666666666666666666666666666666666666666666666666666666666666666666666666666666666666666666666666666666666666666666666666666666666666666666666666666666666666666666666666666666666666666666666666666666666666666666666666666666666666666666666666666666666

0000000000000000000000000000000000000000000000000000000000000000000000000000111111111111111111111111111111111111111111111111111111111111111111111111111111111111111111111111111122222222222222222222222222222222222222222222222222222222222222222222222222222222222222222222222222223333333333333333333333333333333333333333333333333333333333333333333333333333333333333

2222223333333333444444444455555555556666666666777777777788888888889999999999000000000011111111112222222222333333333344444444445555555555666666666677777777778888888888999999999900000000001111111111222222222233333333334444444444555555555566666666667777777777888888888899999999990000000000111111111122222222223333333333444444444455555555556666666666777777777788888

4567890123456789012345678901234567890123456789012345678901234567890123456789012345678901234567890123456789012345678901234567890123456789012345678901234567890123456789012345678901234567890123456789012345678901234567890123456789012345678901234567890123456789012345678901234567890123456789012345678901234567890123456789012345678901234567890123456789012345678901234

CRS TTCTTTCATGGGGAAGCAGATTTGGGTACCACCCAAGTATTGACTCACCCATCAACAACCGCTATGTATTTCGTACATTACTGCCAGCCACCATGAATATTGTACGGTACCATAAATACTTGACCACCTGTAGTACATAAAAACCCAATCCACATCAAAACCCCCTCCCCATGCTTACAAGCAAGTACAGCAATCAACCCTCAACTATCACACATCAACTGCAACTCCAAAGCCACCCCTCACCCACTAGGATACCAACAAACCTACCCACCCTTAACAGTACATAGTACATAAAGCCATTTACCGTACATAGCACATTACAGTCAAATCCCTTCTCGTCCCCATGGATGACCCCCCTCAG

>T164

T164.1.1.1_femur ............................................................................................................ACCATAAATACTTGACCACCTGTAGTAC

T164.1.1.2_femur ............................................................................................................

T164.1.1.3_femur ............................................................................................................

T164.1.1.4_femur ............................................................................................................

T164.1.1.5_femur ............................................................................................................

T164.1.2.1_femur ............................................................................................................

T164.1.2.2_femur ............................................................................................................

T164.1.2.3_femur ............................................................................................................

T164.1.2.4_femur ............................................................................................................

T164.1.2.5_femur ............................................................................................................

T164.2.1.1_femur ............................................................................................................

T164.2.1.2_femur ............................................................................................................

T164.2.1.3_femur ............................................................................................................

T164.2.1.4_femur ............................................................................................................

T164.2.1.5_femur ............................................................................................................

T164.2.2.1_femur ............................................................................................................

T164.2.2.2_femur ............................................................................................................

T164.2.2.3_femur ............................................................................................................

T164.2.2.4_femur ............................................................................................................

T164.2.2.5_femur ............................................................................................................

T164.1.1.1_femur CGCTATGTATTTCGTACATTACTGC.........................................................................................................................................................CCTCACCCACTAGGATACCA

T164.1.1.2_femur .........................................................................................................................................................

T164.1.1.3_femur .........................................................................................................................................................

T164.1.1.4_femur .........................................................................................................................................................

T164.1.1.5_femur .........................................................................................................................................................

T164.1.2.1_femur .........................................................................................................................................................

T164.1.2.2_femur .........................................................................................................................................................

T164.1.2.3_femur .........................................................................................................................................................

T164.1.2.4_femur .........................................................................................................................................................

T164.1.2.5_femur .........................................................................................................................................................

T164.2.1.1_femur .........................................................................................................................................................

T164.2.1.2_femur .........................................................................................................................................................

T164.2.1.3_femur .........................................................................................................................................................

T164.2.1.4_femur .........................................................................................................................................................

T164.2.1.5_femur .........................................................................................................................................................

T164.2.2.1_femur .........................................................................................................................................................

T164.2.2.2_femur .........................................................................................................................................................

T164.2.2.3_femur .........................................................................................................................................................

T164.2.2.4_femur .........................................................................................................................................................

T164.2.2.5_femur .........................................................................................................................................................

T164.1.1.1_femur CAACTATCACACATCAACTGCAA........................................................C................................................................................

T164.1.1.2_femur ........................................................C................................................................................

T164.1.1.3_femur ........................................................C................................................................................

T164.1.1.4_femur ........................................................C................................................................................

T164.1.1.5_femur ........................................................C................................................................................

T164.1.2.1_femur ........................................................C................................................................................

T164.1.2.2_femur ........................................................C................................................................................

T164.1.2.3_femur ........................................................C................................................................................

T164.1.2.4_femur ........................................................C................................................................................

T164.1.2.5_femur ........................................................C................................................................................

T164.2.1.1_femur ........................................................C................................................................................

T164.2.1.2_femur ........................................................C................................................................................

T164.2.1.3_femur ........................................................C................................................................................

T164.2.1.4_femur ........................................................C................................................................................

T164.2.1.5_femur ........................................................C................................................................................

T164.2.2.1_femur ........................................................C................................................................................

T164.2.2.2_femur ........................................................C................................................................................

T164.2.2.3_femur ........................................................C................................................................................

T164.2.2.4_femur ........................................................C................................................................................

T164.2.2.5_femur ........................................................C................................................................................

Consensus TTCTTTCATGGGGAAGCAGATTTGGGTACCACCCAAGTATTGACTCACCCATCAACAACCGCTATGTATTTCGTACATTACTGCCAGCCACCATGAATATTGTACGGTACCATAAATACTTGACCACCTGTAGTACATAAAAACCCAATCCACATCAAAACCCCCTCCCCATGCTTACAAGCAAGTACAGCAATCAACCCTCAACTATCACACATCAACTGCAACTCCAAAGCCACCCCTCACCCACTAGGATACCAACAAACCTACCCACCCTTAACAGCACATAGTACATAAAGCCATTTACCGTACATAGCACATTACAGTCAAATCCCTTCTCGTCCCCATGGATGACCCCCCTCAG

1111111111111111111111111111111111111111111111111111111111111111111111111111111111111111111111111111111111111111111111111111111111111111111111111111111111111111111111111111111111111111111111111111111111111111111111111111111111111111111111111111111111111111111111111111111111111111111111111111111111111111111111111111111111111111111111111111111111111111111111111

6666666666666666666666666666666666666666666666666666666666666666666666666666666666666666666666666666666666666666666666666666666666666666666666666666666666666666666666666666666666666666666666666666666666666666666666666666666666666666666666666666666666666666666666666666666666666666666666666666666666666666666666666666666666666666666666666666666666666666666666666

0000000000000000000000000000000000000000000000000000000000000000000000000000111111111111111111111111111111111111111111111111111111111111111111111111111111111111111111111111111122222222222222222222222222222222222222222222222222222222222222222222222222222222222222222222222222223333333333333333333333333333333333333333333333333333333333333333333333333333333333333

2222223333333333444444444455555555556666666666777777777788888888889999999999000000000011111111112222222222333333333344444444445555555555666666666677777777778888888888999999999900000000001111111111222222222233333333334444444444555555555566666666667777777777888888888899999999990000000000111111111122222222223333333333444444444455555555556666666666777777777788888

4567890123456789012345678901234567890123456789012345678901234567890123456789012345678901234567890123456789012345678901234567890123456789012345678901234567890123456789012345678901234567890123456789012345678901234567890123456789012345678901234567890123456789012345678901234567890123456789012345678901234567890123456789012345678901234567890123456789012345678901234

CRS TTCTTTCATGGGGAAGCAGATTTGGGTACCACCCAAGTATTGACTCACCCATCAACAACCGCTATGTATTTCGTACATTACTGCCAGCCACCATGAATATTGTACGGTACCATAAATACTTGACCACCTGTAGTACATAAAAACCCAATCCACATCAAAACCCCCTCCCCATGCTTACAAGCAAGTACAGCAATCAACCCTCAACTATCACACATCAACTGCAACTCCAAAGCCACCCCTCACCCACTAGGATACCAACAAACCTACCCACCCTTAACAGTACATAGTACATAAAGCCATTTACCGTACATAGCACATTACAGTCAAATCCCTTCTCGTCCCCATGGATGACCCCCCTCAG

>T170

T170.1.1.1_tooth ............................................................................................................ACCATAAATACTTGACCACCTGTAGTAC

T170.1.1.2_tooth ............................................................................................................

T170.1.1.3_tooth ............................................................................................................

T170.1.1.4_tooth ............................................................................................................

T170.1.1.5_tooth ............................................................................................................

T170.1.2.1_tooth ............................................................................................................

T170.1.2.2_tooth ............................................................................................................

T170.1.2.3_tooth ............................................................................................................

T170.1.2.4_tooth ............................................................................................................

T170.1.2.5_tooth ............................................................................................................

T170.2.1.1_tooth ............................................................................................................

T170.2.1.2_tooth ............................................................................................................

T170.2.1.3_tooth ............................................................................................................

T170.2.1.4_tooth ............................................................................................................

T170.2.1.5_tooth ............................................................................................................

T170.2.2.1_tooth ............................................................................................................

T170.2.2.2_tooth ...........................................................................................T................

T170.2.2.3_tooth ............................................................................................................

T170.2.2.4_tooth ............................................................................................................

T170.2.2.5_tooth ............................................................................................................

T170.1.1.1_tooth CGCTATGTATTTCGTACATTACTGC.........................................................................................................................................................CCTCACCCACTAGGATACCA

T170.1.1.2_tooth .........................................................................................................................................................

T170.1.1.3_tooth .........................................................................................................................................................

T170.1.1.4_tooth .........................................................................................................................................................

T170.1.1.5_tooth .........................................................................................................................................................

T170.1.2.1_tooth .........................................................................................................................................................

T170.1.2.2_tooth .........................................................................................................................................................

T170.1.2.3_tooth .........................................................................................................................................................

T170.1.2.4_tooth .........................................................................................................................................................

T170.1.2.5_tooth .........................................................................................................................................................

T170.2.1.1_tooth .........................................................................................................................................................

T170.2.1.2_tooth .....................................A...................................................................................................................

T170.2.1.3_tooth .........................................................................................................................................................

T170.2.1.4_tooth .........................................................................................................................................................

T170.2.1.5_tooth .........................................................................................................................................................

T170.2.2.1_tooth .........................................................................................................................................................

T170.2.2.2_tooth .........................................................................................................................................................

T170.2.2.3_tooth .........................................................................................................................................................

T170.2.2.4_tooth .........................................................................................................................................................

T170.2.2.5_tooth .........................................................................................................................................................

T170.1.1.1_tooth CAACTATCACACATCAACTGCAA.........................................................................................................................................

T170.1.1.2_tooth .........................................................................................................................................

T170.1.1.3_tooth .........................................................................................................................................

T170.1.1.4_tooth .........................................................................................................................................

T170.1.1.5_tooth .........................................................................................................................................

T170.1.2.1_tooth .........................................................................................................................................

T170.1.2.2_tooth .........................................................................................................................................

T170.1.2.3_tooth .........................................................................................................................................

T170.1.2.4_tooth .........................................................................................................................................

T170.1.2.5_tooth .........................................................................................................................................

T170.2.1.1_tooth .........................................................................................................................................

T170.2.1.2_tooth .........................................................................................................................................

T170.2.1.3_tooth .........................................................................................................................................

T170.2.1.4_tooth .........................................................................................................................................

T170.2.1.5_tooth .........................................................................................................................................

T170.2.2.1_tooth .........................................................................................................................................

T170.2.2.2_tooth .........................................................................................................................................

T170.2.2.3_tooth ..................................................................................................A......................................

T170.2.2.4_tooth .........................................................................................................................................

T170.2.2.5_tooth .........................................................................................................................................

Consensus TTCTTTCATGGGGAAGCAGATTTGGGTACCACCCAAGTATTGACTCACCCATCAACAACCGCTATGTATTTCGTACATTACTGCCAGCCACCATGAATATTGTACGGTACCATAAATACTTGACCACCTGTAGTACATAAAAACCCAATCCACATCAAAACCCCCTCCCCATGCTTACAAGCAAGTACAGCAATCAACCCTCAACTATCACACATCAACTGCAACTCCAAAGCCACCCCTCACCCACTAGGATACCAACAAACCTACCCACCCTTAACAGTACATAGTACATAAAGCCATTTACCGTACATAGCACATTACAGTCAAATCCCTTCTCGTCCCCATGGATGACCCCCCTCAG

1111111111111111111111111111111111111111111111111111111111111111111111111111111111111111111111111111111111111111111111111111111111111111111111111111111111111111111111111111111111111111111111111111111111111111111111111111111111111111111111111111111111111111111111111111111111111111111111111111111111111111111111111111111111111111111111111111111111111111111111111

6666666666666666666666666666666666666666666666666666666666666666666666666666666666666666666666666666666666666666666666666666666666666666666666666666666666666666666666666666666666666666666666666666666666666666666666666666666666666666666666666666666666666666666666666666666666666666666666666666666666666666666666666666666666666666666666666666666666666666666666666

0000000000000000000000000000000000000000000000000000000000000000000000000000111111111111111111111111111111111111111111111111111111111111111111111111111111111111111111111111111122222222222222222222222222222222222222222222222222222222222222222222222222222222222222222222222222223333333333333333333333333333333333333333333333333333333333333333333333333333333333333

2222223333333333444444444455555555556666666666777777777788888888889999999999000000000011111111112222222222333333333344444444445555555555666666666677777777778888888888999999999900000000001111111111222222222233333333334444444444555555555566666666667777777777888888888899999999990000000000111111111122222222223333333333444444444455555555556666666666777777777788888

4567890123456789012345678901234567890123456789012345678901234567890123456789012345678901234567890123456789012345678901234567890123456789012345678901234567890123456789012345678901234567890123456789012345678901234567890123456789012345678901234567890123456789012345678901234567890123456789012345678901234567890123456789012345678901234567890123456789012345678901234

CRS TTCTTTCATGGGGAAGCAGATTTGGGTACCACCCAAGTATTGACTCACCCATCAACAACCGCTATGTATTTCGTACATTACTGCCAGCCACCATGAATATTGTACGGTACCATAAATACTTGACCACCTGTAGTACATAAAAACCCAATCCACATCAAAACCCCCTCCCCATGCTTACAAGCAAGTACAGCAATCAACCCTCAACTATCACACATCAACTGCAACTCCAAAGCCACCCCTCACCCACTAGGATACCAACAAACCTACCCACCCTTAACAGTACATAGTACATAAAGCCATTTACCGTACATAGCACATTACAGTCAAATCCCTTCTCGTCCCCATGGATGACCCCCCTCAG

>T170

T170.1.1.1_femur .....................................................................C......................................ACCATAAATACTTGACCACCTGTAGTAC

T170.1.1.2_femur .....................................................................C......................................

T170.1.1.3_femur .....................................................................C......................................

T170.1.1.4_femur .....................................................................C......................................

T170.1.1.5_femur .....................................................................C......................................

T170.1.2.1_femur .....................................................................C......................................

T170.1.2.2_femur .....................................................................C......................................

T170.1.2.3_femur .....................................................................C......................................

T170.1.2.4_femur .....................................................................C......................................

T170.1.2.5_femur .....................................................................C......................................

T170.2.1.1_femur .....................................................................C......................................

T170.2.1.2_femur .....................................................................C......................................

T170.2.1.3_femur .....................................................................C......................................

T170.2.1.4_femur .....................................................................C......................................

T170.2.1.5_femur .....................................................................C......................................

T170.2.2.1_femur .....................................................................C......................................

T170.2.2.2_femur .....................................................................C......................................

T170.2.2.3_femur .....................................................................C......................................

T170.2.2.4_femur .....................................................................C......................................

T170.2.2.5_femur .....................................................................C......................................

T170.1.1.1_femur CGCTATGTATTTCGTACATTACTGC.........................................................................................................................................................CCTCACCCACTAGGATACCA

T170.1.1.2_femur .........................................................................................................................................................

T170.1.1.3_femur .........................................................................................................................................................

T170.1.1.4_femur .........................................................................................................................................................

T170.1.1.5_femur .........................................................................................................................................................

T170.1.2.1_femur .........................................................................................................................................................

T170.1.2.2_femur .........................................................................................................................................................

T170.1.2.3_femur .........................................................................................................................................................

T170.1.2.4_femur .........................................................................................................................................................

T170.1.2.5_femur .........................................................................................................................................................

T170.2.1.1_femur .........................................................................................................................................................

T170.2.1.2_femur .........................................................................................................................................................

T170.2.1.3_femur .........................................................................................................................................................

T170.2.1.4_femur .........................................................................................................................................................

T170.2.1.5_femur .........................................................................................................................................................

T170.2.2.1_femur .........................................................................................................................................................

T170.2.2.2_femur .........................................................................................................................................................

T170.2.2.3_femur .........................................................................................................................................................

T170.2.2.4_femur .........................................................................................................................................................

T170.2.2.5_femur .........................................................................................................................................................

T170.1.1.1_femur CAACTATCACACATCAACTGCAA.........................................................................................................................................

T170.1.1.2_femur .........................................................................................................................................

T170.1.1.3_femur .........................................................................................................................................

T170.1.1.4_femur .........................................................................................................................................

T170.1.1.5_femur .........................................................................................................................................

T170.1.2.1_femur .........................................................................................................................................

T170.1.2.2_femur .........................................................................................................................................

T170.1.2.3_femur .........................................................................................................................................

T170.1.2.4_femur .........................................................................................................................................

T170.1.2.5_femur .........................................................................................................................................

T170.2.1.1_femur .........................................................................................................................................

T170.2.1.2_femur .........................................................................................................................................

T170.2.1.3_femur .........................................................................................................................................

T170.2.1.4_femur .........................................................................................................................................

T170.2.1.5_femur .........................................................................................................................................

T170.2.2.1_femur .........................................................................................................................................

T170.2.2.2_femur .........................................................................................................................................

T170.2.2.3_femur .........................................................................................................................................

T170.2.2.4_femur .........................................................................................................................................

T170.2.2.5_femur .........................................................................................................................................

Consensus TTCTTTCATGGGGAAGCAGATTTGGGTACCACCCAAGTATTGACTCACCCATCAACAACCGCTATGTATCTCGTACATTACTGCCAGCCACCATGAATATTGTACGGTACCATAAATACTTGACCACCTGTAGTACATAAAAACCCAATCCACATCAAAACCCCCTCCCCATGCTTACAAGCAAGTACAGCAATCAACCCTCAACTATCACACATCAACTGCAACTCCAAAGCCACCCCTCACCCACTAGGATACCAACAAACCTACCCACCCTTAACAGTACATAGTACATAAAGCCATTTACCGTACATAGCACATTACAGTCAAATCCCTTCTCGTCCCCATGGATGACCCCCCTCAG

1111111111111111111111111111111111111111111111111111111111111111111111111111111111111111111111111111111111111111111111111111111111111111111111111111111111111111111111111111111111111111111111111111111111111111111111111111111111111111111111111111111111111111111111111111111111111111111111111111111111111111111111111111111111111111111111111111111111111111111111111

6666666666666666666666666666666666666666666666666666666666666666666666666666666666666666666666666666666666666666666666666666666666666666666666666666666666666666666666666666666666666666666666666666666666666666666666666666666666666666666666666666666666666666666666666666666666666666666666666666666666666666666666666666666666666666666666666666666666666666666666666

0000000000000000000000000000000000000000000000000000000000000000000000000000111111111111111111111111111111111111111111111111111111111111111111111111111111111111111111111111111122222222222222222222222222222222222222222222222222222222222222222222222222222222222222222222222222223333333333333333333333333333333333333333333333333333333333333333333333333333333333333

2222223333333333444444444455555555556666666666777777777788888888889999999999000000000011111111112222222222333333333344444444445555555555666666666677777777778888888888999999999900000000001111111111222222222233333333334444444444555555555566666666667777777777888888888899999999990000000000111111111122222222223333333333444444444455555555556666666666777777777788888

4567890123456789012345678901234567890123456789012345678901234567890123456789012345678901234567890123456789012345678901234567890123456789012345678901234567890123456789012345678901234567890123456789012345678901234567890123456789012345678901234567890123456789012345678901234567890123456789012345678901234567890123456789012345678901234567890123456789012345678901234

CRS TTCTTTCATGGGGAAGCAGATTTGGGTACCACCCAAGTATTGACTCACCCATCAACAACCGCTATGTATTTCGTACATTACTGCCAGCCACCATGAATATTGTACGGTACCATAAATACTTGACCACCTGTAGTACATAAAAACCCAATCCACATCAAAACCCCCTCCCCATGCTTACAAGCAAGTACAGCAATCAACCCTCAACTATCACACATCAACTGCAACTCCAAAGCCACCCCTCACCCACTAGGATACCAACAAACCTACCCACCCTTAACAGTACATAGTACATAAAGCCATTTACCGTACATAGCACATTACAGTCAAATCCCTTCTCGTCCCCATGGATGACCCCCCTCAG

>T176

T176.1.1.1_tooth ............................................................................................................ACCATAAATACTTGACCACCTGTAGTAC

T176.1.1.2_tooth ............................................................................................................

T176.1.1.3_tooth ............................................................................................................

T176.1.1.4_tooth ............................................................................................................

T176.1.1.5_tooth ............................................................................................................

T176.1.2.1_tooth ............................................................................................................

T176.1.2.2_tooth ............................................................................................................

T176.1.2.3_tooth ............................................................................................................

T176.1.2.4_tooth ............................................................................................................

T176.1.2.5_tooth ............................................................................................................

T176.2.1.1_tooth ............................................................................................................

T176.2.1.2_tooth ............................................................................................................

T176.2.1.3_tooth ............................................................................................................

T176.2.1.4_tooth ............................................................................................................

T176.2.1.5_tooth ............................................................................................................

T176.2.2.1_tooth ............................................................................................................

T176.2.2.2_tooth ............................................................................................................

T176.2.2.3_tooth ............................................................................................................

T176.2.2.4_tooth ............................................................................................................

T176.2.2.5_tooth ............................................................................................................

T176.1.1.1_tooth CGCTATGTATTTCGTACATTACTGC................................................................C........................................................................................CCTCACCCACTAGGATACCA

T176.1.1.2_tooth ................................................................C........................................................................................

T176.1.1.3_tooth ................................................................C........................................................................................

T176.1.1.4_tooth ................................................................C.......................................................T................................

T176.1.1.5_tooth ................................................................C........................................................................................

T176.1.2.1_tooth ................................................................C........................................................................................

T176.1.2.2_tooth ................................................................C........................................................................................

T176.1.2.3_tooth ................................................................C........................................................................................

T176.1.2.4_tooth ................................................................C........................................................................................

T176.1.2.5_tooth ................................................................C........................................................................................

T176.2.1.1_tooth ................................................................C........................................................................................

T176.2.1.2_tooth ................................................................C........................................................................................

T176.2.1.3_tooth ..........................T.....................................C........................................................................................

T176.2.1.4_tooth ................................................................C........................................................................................

T176.2.1.5_tooth ................................................................C........................................................................................

T176.2.2.1_tooth ................................................................C........................................................................................

T176.2.2.2_tooth ................................................................C........................................................................................

T176.2.2.3_tooth ................................................................C........................................................................................

T176.2.2.4_tooth ................................................................C........................................................................................

T176.2.2.5_tooth ................................................................C........................................................................................

T176.1.1.1_tooth CAACTATCACACATCAACTGCAA.........................................................................................................................................

T176.1.1.2_tooth .........................................................................................................................................

T176.1.1.3_tooth .........................................................................................................................................

T176.1.1.4_tooth .........................................................................................................................................

T176.1.1.5_tooth .........................................................................................................................................

T176.1.2.1_tooth .........................................................................................................................................

T176.1.2.2_tooth .........................................................................................................................................

T176.1.2.3_tooth .........................................................................................................................................

T176.1.2.4_tooth .........................................................................................................................................

T176.1.2.5_tooth .........................................................................................................................................

T176.2.1.1_tooth .........................................................................................................................................

T176.2.1.2_tooth .........................................................................................................................................

T176.2.1.3_tooth .........................................................................................................................................

T176.2.1.4_tooth .........................................................................................................................................

T176.2.1.5_tooth .........................................................................................................................................

T176.2.2.1_tooth .........................A...............................................................................................................

T176.2.2.2_tooth .........................................................................................................................................

T176.2.2.3_tooth .........................................................................................................................................

T176.2.2.4_tooth .........................................................................................................................................

T176.2.2.5_tooth .........................................................................................................................................

Consensus TTCTTTCATGGGGAAGCAGATTTGGGTACCACCCAAGTATTGACTCACCCATCAACAACCGCTATGTATTTCGTACATTACTGCCAGCCACCATGAATATTGTACGGTACCATAAATACTTGACCACCTGTAGTACATAAAAACCCAACCCACATCAAAACCCCCTCCCCATGCTTACAAGCAAGTACAGCAATCAACCCTCAACTATCACACATCAACTGCAACTCCAAAGCCACCCCTCACCCACTAGGATACCAACAAACCTACCCACCCTTAACAGTACATAGTACATAAAGCCATTTACCGTACATAGCACATTACAGTCAAATCCCTTCTCGTCCCCATGGATGACCCCCCTCAG

1111111111111111111111111111111111111111111111111111111111111111111111111111111111111111111111111111111111111111111111111111111111111111111111111111111111111111111111111111111111111111111111111111111111111111111111111111111111111111111111111111111111111111111111111111111111111111111111111111111111111111111111111111111111111111111111111111111111111111111111111

6666666666666666666666666666666666666666666666666666666666666666666666666666666666666666666666666666666666666666666666666666666666666666666666666666666666666666666666666666666666666666666666666666666666666666666666666666666666666666666666666666666666666666666666666666666666666666666666666666666666666666666666666666666666666666666666666666666666666666666666666

0000000000000000000000000000000000000000000000000000000000000000000000000000111111111111111111111111111111111111111111111111111111111111111111111111111111111111111111111111111122222222222222222222222222222222222222222222222222222222222222222222222222222222222222222222222222223333333333333333333333333333333333333333333333333333333333333333333333333333333333333

2222223333333333444444444455555555556666666666777777777788888888889999999999000000000011111111112222222222333333333344444444445555555555666666666677777777778888888888999999999900000000001111111111222222222233333333334444444444555555555566666666667777777777888888888899999999990000000000111111111122222222223333333333444444444455555555556666666666777777777788888

4567890123456789012345678901234567890123456789012345678901234567890123456789012345678901234567890123456789012345678901234567890123456789012345678901234567890123456789012345678901234567890123456789012345678901234567890123456789012345678901234567890123456789012345678901234567890123456789012345678901234567890123456789012345678901234567890123456789012345678901234

CRS TTCTTTCATGGGGAAGCAGATTTGGGTACCACCCAAGTATTGACTCACCCATCAACAACCGCTATGTATTTCGTACATTACTGCCAGCCACCATGAATATTGTACGGTACCATAAATACTTGACCACCTGTAGTACATAAAAACCCAATCCACATCAAAACCCCCTCCCCATGCTTACAAGCAAGTACAGCAATCAACCCTCAACTATCACACATCAACTGCAACTCCAAAGCCACCCCTCACCCACTAGGATACCAACAAACCTACCCACCCTTAACAGTACATAGTACATAAAGCCATTTACCGTACATAGCACATTACAGTCAAATCCCTTCTCGTCCCCATGGATGACCCCCCTCAG

>T176

T176.1.1.1_rib ............................................................................................................ACCATAAATACTTGACCACCTGTAGTAC

T176.1.1.2_rib ............................................................................................................

T176.1.1.3_rib ............................................................................................................

T176.1.1.4_rib ............................................................................................................

T176.1.1.5_rib ............................................................................................................

T176.1.2.1_rib ............................................................................................................

T176.1.2.2_rib ............................................................................................................

T176.1.2.3_rib ............................................................................................................

T176.1.2.4_rib ............................................................................................................

T176.1.2.5_rib ............................................................................................................

T176.2.1.1_rib ............................................................................................................

T176.2.1.2_rib ............................................................................................................

T176.2.1.3_rib ............................................................................................................

T176.2.1.4_rib ............................................................................................................

T176.2.1.5_rib ............................................................................................................

T176.2.2.1_rib ............................................................................................................

T176.2.2.2_rib ............................................................................................................

T176.2.2.3_rib ............................................................................................................

T176.2.2.4_rib ............................................................................................................

T176.2.2.5_rib ............................................................................................................

T176.1.1.1_rib CGCTATGTATTTCGTACATTACTGC.........................................................................................................................................................CCTCACCCACTAGGATACCA

T176.1.1.2_rib .........................................................................................................................................................

T176.1.1.3_rib .........................................................................................................................................................

T176.1.1.4_rib .........................................................................................................................................................

T176.1.1.5_rib .........................................................................................................................................................

T176.1.2.1_rib .........................................................................................................................................................

T176.1.2.2_rib .........................................................................................................................................................

T176.1.2.3_rib .........................................................................................................................................................

T176.1.2.4_rib .........................................................................................................................................................

T176.1.2.5_rib .........................................................................................................................................................

T176.2.1.1_rib .........................................................................................................................................................

T176.2.1.2_rib .........................................................................................................................................................

T176.2.1.3_rib .........................................................................................................................................................

T176.2.1.4_rib .........................................................................................................................................................

T176.2.1.5_rib .........................................................................................................................................................

T176.2.2.1_rib .........................................................................................................................................................

T176.2.2.2_rib .........................................................................................................................................................

T176.2.2.3_rib .........................................................................................................................................................

T176.2.2.4_rib .........................................................................................................................................................

T176.2.2.5_rib .........................................................................................................................................................

T176.1.1.1_rib CAACTATCACACATCAACTGCAA.........................................................................................................................................

T176.1.1.2_rib .........................................................................................................................................

T176.1.1.3_rib .......................................................................................................................................

T176.1.1.4_rib .........................................................................................................................................

T176.1.1.5_rib .........................................................................................................................................

T176.1.2.1_rib .........................................................................................................................................

T176.1.2.2_rib .........................................................................................................................................

T176.1.2.3_rib .........................................................................................................................................

T176.1.2.4_rib .........................................................................................................................................

T176.1.2.5_rib .........................................................................................................................................

T176.2.1.1_rib .........................................................................................................................................

T176.2.1.2_rib .........................................................................................................................................

T176.2.1.3_rib .........................................................................................................................................

T176.2.1.4_rib .........................................................................................................................................

T176.2.1.5_rib .........................................................................................................................................

T176.2.2.1_rib .........................................................................................................................................

T176.2.2.2_rib .........................................................................................................................................

T176.2.2.3_rib .........................................................................................................................................

T176.2.2.4_rib .........................................................................................................................................

T176.2.2.5_rib .........................................................................................................................................

Consensus TTCTTTCATGGGGAAGCAGATTTGGGTACCACCCAAGTATTGACTCACCCATCAACAACCGCTATGTATTTCGTACATTACTGCCAGCCACCATGAATATTGTACGGTACCATAAATACTTGACCACCTGTAGTACATAAAAACCCAATCCACATCAAAACCCCCTCCCCATGCTTACAAGCAAGTACAGCAATCAACCCTCAACTATCACACATCAACTGCAACTCCAAAGCCACCCCTCACCCACTAGGATACCAACAAACCTACCCACCCTTAACAGTACATAGTACATAAAGCCATTTACCGTACATAGCACATTACAGTCAAATCCCTTCTCGTCCCCATGGATGACCCCCCTCAG

1111111111111111111111111111111111111111111111111111111111111111111111111111111111111111111111111111111111111111111111111111111111111111111111111111111111111111111111111111111111111111111111111111111111111111111111111111111111111111111111111111111111111111111111111111111111111111111111111111111111111111111111111111111111111111111111111111111111111111111111111

6666666666666666666666666666666666666666666666666666666666666666666666666666666666666666666666666666666666666666666666666666666666666666666666666666666666666666666666666666666666666666666666666666666666666666666666666666666666666666666666666666666666666666666666666666666666666666666666666666666666666666666666666666666666666666666666666666666666666666666666666

0000000000000000000000000000000000000000000000000000000000000000000000000000111111111111111111111111111111111111111111111111111111111111111111111111111111111111111111111111111122222222222222222222222222222222222222222222222222222222222222222222222222222222222222222222222222223333333333333333333333333333333333333333333333333333333333333333333333333333333333333

2222223333333333444444444455555555556666666666777777777788888888889999999999000000000011111111112222222222333333333344444444445555555555666666666677777777778888888888999999999900000000001111111111222222222233333333334444444444555555555566666666667777777777888888888899999999990000000000111111111122222222223333333333444444444455555555556666666666777777777788888

4567890123456789012345678901234567890123456789012345678901234567890123456789012345678901234567890123456789012345678901234567890123456789012345678901234567890123456789012345678901234567890123456789012345678901234567890123456789012345678901234567890123456789012345678901234567890123456789012345678901234567890123456789012345678901234567890123456789012345678901234

CRS TTCTTTCATGGGGAAGCAGATTTGGGTACCACCCAAGTATTGACTCACCCATCAACAACCGCTATGTATTTCGTACATTACTGCCAGCCACCATGAATATTGTACGGTACCATAAATACTTGACCACCTGTAGTACATAAAAACCCAATCCACATCAAAACCCCCTCCCCATGCTTACAAGCAAGTACAGCAATCAACCCTCAACTATCACACATCAACTGCAACTCCAAAGCCACCCCTCACCCACTAGGATACCAACAAACCTACCCACCCTTAACAGTACATAGTACATAAAGCCATTTACCGTACATAGCACATTACAGTCAAATCCCTTCTCGTCCCCATGGATGACCCCCCTCAG

>T176

T176.1.1.1_femur ............................................................................................................ACCATAAATACTTGACCACCTGTAGTAC

T176.1.1.2_femur ............................................................................................................

T176.1.1.3_femur ............................................................................................................

T176.1.1.4_femur ............................................................................................................

T176.1.1.5_femur ............................................................................................................

T176.1.2.1_femur ............................................................................................................

T176.1.2.2_femur ............................................................................................................

T176.1.2.3_femur ............................................................................................................

T176.1.2.4_femur ............................................................................................................

T176.1.2.5_femur ............................................................................................................

T176.2.1.1_femur ............................................................................................................

T176.2.1.2_femur ............................................................................................................

T176.2.1.3_femur ............................................................................................................

T176.2.1.4_femur ............................................................................................................

T176.2.1.5_femur ............................................................................................................

T176.2.2.1_femur ............................................................................................................

T176.2.2.2_femur ............................................................................................................

T176.2.2.3_femur ............................................................................................................

T176.2.2.4_femur ............................................................................................................

T176.2.2.5_femur ............................................................................................................

T176.1.1.1_femur CGCTATGTATTTCGTACATTACTGC.........................................................................................................................................................CCTCACCCACTAGGATACCA

T176.1.1.2_femur .........................................................................................................................................................

T176.1.1.3_femur .........................................................................................................................................................

T176.1.1.4_femur .........................................................................................................................................................

T176.1.1.5_femur .........................................................................................................................................................

T176.1.2.1_femur .........................................................................................................................................................

T176.1.2.2_femur .........................................................................................................................................................

T176.1.2.3_femur .........................................................................................................................................................

T176.1.2.4_femur .........................................................................................................................................................

T176.1.2.5_femur .........................................................................................................................................................

T176.2.1.1_femur .........................................................................................................................................................

T176.2.1.2_femur .........................................................................................................................................................

T176.2.1.3_femur .........................................................................................................................................................

T176.2.1.4_femur .........................................................................................................................................................

T176.2.1.5_femur .........................................................................................................................................................

T176.2.2.1_femur .........................................................................................................................................................

T176.2.2.2_femur .........................................................................................................................................................

T176.2.2.3_femur .........................................................................................................................................................

T176.2.2.4_femur .........................................................................................................................................................

T176.2.2.5_femur .........................................................................................................................................................

T176.1.1.1_femur CAACTATCACACATCAACTGCAA........................................................C................................................................................

T176.1.1.2_femur ........................................................C................................................................................

T176.1.1.3_femur ........................................................C................................................................................

T176.1.1.4_femur ........................................................C................................................................................

T176.1.1.5_femur ........................................................C................................................................................

T176.1.2.1_femur ........................................................C................................................................................

T176.1.2.2_femur ........................................................C................................................................................

T176.1.2.3_femur ........................................................C................................................................................

T176.1.2.4_femur ........................................................C................................................................................

T176.1.2.5_femur ........................................................C................................................................................

T176.2.1.1_femur ........................................................C................................................................................

T176.2.1.2_femur ........................................................C................................................................................

T176.2.1.3_femur ........................................................C................................................................................

T176.2.1.4_femur ........................................................C................................................................................

T176.2.1.5_femur ........................................................C................................................................................

T176.2.2.1_femur ........................................................C................................................................................

T176.2.2.2_femur ........................................................C................................................................................

T176.2.2.3_femur ........................................................C................................................................................

T176.2.2.4_femur ........................................................C................................................................................

T176.2.2.5_femur ........................................................C................................................................................

Consensus TTCTTTCATGGGGAAGCAGATTTGGGTACCACCCAAGTATTGACTCACCCATCAACAACCGCTATGTATTTCGTACATTACTGCCAGCCACCATGAATATTGTACGGTACCATAAATACTTGACCACCTGTAGTACATAAAAACCCAATCCACATCAAAACCCCCTCCCCATGCTTACAAGCAAGTACAGCAATCAACCCTCAACTATCACACATCAACTGCAACTCCAAAGCCACCCCTCACCCACTAGGATACCAACAAACCTACCCACCCTTAACAGCACATAGTACATAAAGCCATTTACCGTACATAGCACATTACAGTCAAATCCCTTCTCGTCCCCATGGATGACCCCCCTCAG

1111111111111111111111111111111111111111111111111111111111111111111111111111111111111111111111111111111111111111111111111111111111111111111111111111111111111111111111111111111111111111111111111111111111111111111111111111111111111111111111111111111111111111111111111111111111111111111111111111111111111111111111111111111111111111111111111111111111111111111111111

6666666666666666666666666666666666666666666666666666666666666666666666666666666666666666666666666666666666666666666666666666666666666666666666666666666666666666666666666666666666666666666666666666666666666666666666666666666666666666666666666666666666666666666666666666666666666666666666666666666666666666666666666666666666666666666666666666666666666666666666666

0000000000000000000000000000000000000000000000000000000000000000000000000000111111111111111111111111111111111111111111111111111111111111111111111111111111111111111111111111111122222222222222222222222222222222222222222222222222222222222222222222222222222222222222222222222222223333333333333333333333333333333333333333333333333333333333333333333333333333333333333

2222223333333333444444444455555555556666666666777777777788888888889999999999000000000011111111112222222222333333333344444444445555555555666666666677777777778888888888999999999900000000001111111111222222222233333333334444444444555555555566666666667777777777888888888899999999990000000000111111111122222222223333333333444444444455555555556666666666777777777788888

4567890123456789012345678901234567890123456789012345678901234567890123456789012345678901234567890123456789012345678901234567890123456789012345678901234567890123456789012345678901234567890123456789012345678901234567890123456789012345678901234567890123456789012345678901234567890123456789012345678901234567890123456789012345678901234567890123456789012345678901234

CRS TTCTTTCATGGGGAAGCAGATTTGGGTACCACCCAAGTATTGACTCACCCATCAACAACCGCTATGTATTTCGTACATTACTGCCAGCCACCATGAATATTGTACGGTACCATAAATACTTGACCACCTGTAGTACATAAAAACCCAATCCACATCAAAACCCCCTCCCCATGCTTACAAGCAAGTACAGCAATCAACCCTCAACTATCACACATCAACTGCAACTCCAAAGCCACCCCTCACCCACTAGGATACCAACAAACCTACCCACCCTTAACAGTACATAGTACATAAAGCCATTTACCGTACATAGCACATTACAGTCAAATCCCTTCTCGTCCCCATGGATGACCCCCCTCAG

>T176

T176.1.1.1_ulna ............................................................................................................ACCATAAATACTTGACCACCTGTAGTAC

T176.1.1.2_ulna ............................................................................................................

T176.1.1.3_ulna ............................................................................................................

T176.1.1.4_ulna ............................................................................................................

T176.1.1.5_ulna ............................................................................................................

T176.1.2.1_ulna ............................................................................................................

T176.1.2.2_ulna ............................................................................................................

T176.1.2.3_ulna ............................................................................................................

T176.1.2.4_ulna ............................................................................................................

T176.1.2.5_ulna ............................................................................................................

T176.2.1.1_ulna ............................................................................................................

T176.2.1.2_ulna ............................................................................................................

T176.2.1.3_ulna ............................................................................................................

T176.2.1.4_ulna ............................................................................................................

T176.2.1.5_ulna ............................................................................................................

T176.2.2.1_ulna ............................................................................................................

T176.2.2.2_ulna ............................................................................................................

T176.2.2.3_ulna ............................................................................................................

T176.2.2.4_ulna ............................................................................................................

T176.2.2.5_ulna ............................................................................................................

T176.1.1.1_ulna CGCTATGTATTTCGTACATTACTGC.........................................................................................................................................................CCTCACCCACTAGGATACCA

T176.1.1.2_ulna .........................................................................................................................................................

T176.1.1.3_ulna .........................................................................................................................................................

T176.1.1.4_ulna .........................................................................................................................................................

T176.1.1.5_ulna .........................................................................................................................................................

T176.1.2.1_ulna .........................................................................................................................................................

T176.1.2.2_ulna .........................................................................................................................................................

T176.1.2.3_ulna .........................................................................................................................................................

T176.1.2.4_ulna .........................................................................................................................................................

T176.1.2.5_ulna .........................................................................................................................................................

T176.2.1.1_ulna .........................................................................................................................................................

T176.2.1.2_ulna .........................................................................................................................................................

T176.2.1.3_ulna .........................................................................................................................................................

T176.2.1.4_ulna .........................................................................................................................................................

T176.2.1.5_ulna .........................................................................................................................................................

T176.2.2.1_ulna .........................................................................................................................................................

T176.2.2.2_ulna .........................................................................................................................................................

T176.2.2.3_ulna .........................................................................................................................................................

T176.2.2.4_ulna .........................................................................................................................................................

T176.2.2.5_ulna .........................................................................................................................................................

T176.1.1.1_ulna CAACTATCACACATCAACTGCAA.........................................................................................................................................

T176.1.1.2_ulna .........................................................................................................................................

T176.1.1.3_ulna .........................................................................................................................................

T176.1.1.4_ulna .........................................................................................................................................

T176.1.1.5_ulna .........................................................................................................................................

T176.1.2.1_ulna .........................................................................................................................................

T176.1.2.2_ulna .........................................................................................................................................

T176.1.2.3_ulna .........................................................................................................................................

T176.1.2.4_ulna .........................................................................................................................................

T176.1.2.5_ulna .........................................................................................................................................

T176.2.1.1_ulna .........................................................................................................................................

T176.2.1.2_ulna .........................................................................................................................................

T176.2.1.3_ulna .........................................................................................................................................

T176.2.1.4_ulna .........................................................................................................................................

T176.2.1.5_ulna .........................................................................................................................................

T176.2.2.1_ulna .........................................................................................................................................

T176.2.2.2_ulna .........................................................................................................................................

T176.2.2.3_ulna .........................................................................................................................................

T176.2.2.4_ulna .........................................................................................................................................

T176.2.2.5_ulna .........................................................................................................................................

Consensus TTCTTTCATGGGGAAGCAGATTTGGGTACCACCCAAGTATTGACTCACCCATCAACAACCGCTATGTATTTCGTACATTACTGCCAGCCACCATGAATATTGTACGGTACCATAAATACTTGACCACCTGTAGTACATAAAAACCCAATCCACATCAAAACCCCCTCCCCATGCTTACAAGCAAGTACAGCAATCAACCCTCAACTATCACACATCAACTGCAACTCCAAAGCCACCCCTCACCCACTAGGATACCAACAAACCTACCCACCCTTAACAGTACATAGTACATAAAGCCATTTACCGTACATAGCACATTACAGTCAAATCCCTTCTCGTCCCCATGGATGACCCCCCTCAG

1111111111111111111111111111111111111111111111111111111111111111111111111111111111111111111111111111111111111111111111111111111111111111111111111111111111111111111111111111111111111111111111111111111111111111111111111111111111111111111111111111111111111111111111111111111111111111111111111111111111111111111111111111111111111111111111111111111111111111111111111

6666666666666666666666666666666666666666666666666666666666666666666666666666666666666666666666666666666666666666666666666666666666666666666666666666666666666666666666666666666666666666666666666666666666666666666666666666666666666666666666666666666666666666666666666666666666666666666666666666666666666666666666666666666666666666666666666666666666666666666666666

0000000000000000000000000000000000000000000000000000000000000000000000000000111111111111111111111111111111111111111111111111111111111111111111111111111111111111111111111111111122222222222222222222222222222222222222222222222222222222222222222222222222222222222222222222222222223333333333333333333333333333333333333333333333333333333333333333333333333333333333333

2222223333333333444444444455555555556666666666777777777788888888889999999999000000000011111111112222222222333333333344444444445555555555666666666677777777778888888888999999999900000000001111111111222222222233333333334444444444555555555566666666667777777777888888888899999999990000000000111111111122222222223333333333444444444455555555556666666666777777777788888

4567890123456789012345678901234567890123456789012345678901234567890123456789012345678901234567890123456789012345678901234567890123456789012345678901234567890123456789012345678901234567890123456789012345678901234567890123456789012345678901234567890123456789012345678901234567890123456789012345678901234567890123456789012345678901234567890123456789012345678901234

CRS TTCTTTCATGGGGAAGCAGATTTGGGTACCACCCAAGTATTGACTCACCCATCAACAACCGCTATGTATTTCGTACATTACTGCCAGCCACCATGAATATTGTACGGTACCATAAATACTTGACCACCTGTAGTACATAAAAACCCAATCCACATCAAAACCCCCTCCCCATGCTTACAAGCAAGTACAGCAATCAACCCTCAACTATCACACATCAACTGCAACTCCAAAGCCACCCCTCACCCACTAGGATACCAACAAACCTACCCACCCTTAACAGTACATAGTACATAAAGCCATTTACCGTACATAGCACATTACAGTCAAATCCCTTCTCGTCCCCATGGATGACCCCCCTCAG

>T189

T189.1.1.1_tooth ............................................................................................................ACCATAAATACTTGACCACCTGTAGTAC

T189.1.1.2_tooth ............................................................................................................

T189.1.1.3_tooth ............................................................................................................

T189.1.1.4_tooth ............................................................................................................

T189.1.1.5_tooth ............................................................................................................

T189.1.2.1_tooth ...........................................T................................................................

T189.1.2.2_tooth ............................................................................................................

T189.1.2.3_tooth ............................................................................................................

T189.1.2.4_tooth ............................................................................................................

T189.1.2.5_tooth ............................................................................................................

T189.2.1.1_tooth ............................................................................................................

T189.2.1.2_tooth ............................................................................................................

T189.2.1.3_tooth ............................................................................................................

T189.2.1.4_tooth ............................................................................................................

T189.2.1.5_tooth ............................................................................................................

T189.2.2.1_tooth ............................................................................................................

T189.2.2.2_tooth ............................................................................................................

T189.2.2.3_tooth ............................................................................................................

T189.2.2.4_tooth ............................................................................................................

T189.2.2.5_tooth ............................................................................................................

T189.1.1.1_tooth CGCTATGTATTTCGTACATTACTGC.........................................................................................................................................................CCTCACCCACTAGGATACCA

T189.1.1.2_tooth .........................................................................................................................................................

T189.1.1.3_tooth .........................................................................................................................................................

T189.1.1.4_tooth .........................................................................................................................................................

T189.1.1.5_tooth .........................................................................................................................................................

T189.1.2.1_tooth .........................................................................................................................................................

T189.1.2.2_tooth .........................................................................................................................................................

T189.1.2.3_tooth .........................................................................................................................................................

T189.1.2.4_tooth .........................................................................................................................................................

T189.1.2.5_tooth .........................................................................................................................................................

T189.2.1.1_tooth .........................................................................................................................................................

T189.2.1.2_tooth ......................A............................................................................................................................A.....

T189.2.1.3_tooth .........................................................................................................................................................

T189.2.1.4_tooth .........................................................................................................................................................

T189.2.1.5_tooth .........................................................................................................................................................

T189.2.2.1_tooth .........................................................................................................................................................

T189.2.2.2_tooth .........................................................................................................................................................

T189.2.2.3_tooth .........................................................................................................................................................

T189.2.2.4_tooth .........................................................................................................................................................

T189.2.2.5_tooth .........................................................................................................................................................

T189.1.1.1_tooth CAACTATCACACATCAACTGCAA.........................................................................................................................................

T189.1.1.2_tooth .........................................................................................................................................

T189.1.1.3_tooth .........................................................................................................................................

T189.1.1.4_tooth .........................................................................................................................................

T189.1.1.5_tooth .........................................................................................................................................

T189.1.2.1_tooth .........................................................................................................................................

T189.1.2.2_tooth .........................................................................................................................................

T189.1.2.3_tooth .........................................................................................................................................

T189.1.2.4_tooth .........................................................................................................................................

T189.1.2.5_tooth .........................................................................................................................................

T189.2.1.1_tooth .........................................................................................................................................

T189.2.1.2_tooth .........................................................................................................................................

T189.2.1.3_tooth .........................................................................................................................................

T189.2.1.4_tooth .........................................................................................................................................

T189.2.1.5_tooth .........................................................................................................................................

T189.2.2.1_tooth .........................................................................................................................................

T189.2.2.2_tooth .........................................................................................................................................

T189.2.2.3_tooth .........................................................................................................................................

T189.2.2.4_tooth .........................................................................................................................................

T189.2.2.5_tooth .........................................................................................................................................

Consensus TTCTTTCATGGGGAAGCAGATTTGGGTACCACCCAAGTATTGACTCACCCATCAACAACCGCTATGTATTTCGTACATTACTGCCAGCCACCATGAATATTGTACGGTACCATAAATACTTGACCACCTGTAGTACATAAAAACCCAATCCACATCAAAACCCCCTCCCCATGCTTACAAGCAAGTACAGCAATCAACCCTCAACTATCACACATCAACTGCAACTCCAAAGCCACCCCTCACCCACTAGGATACCAACAAACCTACCCACCCTTAACAGTACATAGTACATAAAGCCATTTACCGTACATAGCACATTACAGTCAAATCCCTTCTCGTCCCCATGGATGACCCCCCTCAG

1111111111111111111111111111111111111111111111111111111111111111111111111111111111111111111111111111111111111111111111111111111111111111111111111111111111111111111111111111111111111111111111111111111111111111111111111111111111111111111111111111111111111111111111111111111111111111111111111111111111111111111111111111111111111111111111111111111111111111111111111

6666666666666666666666666666666666666666666666666666666666666666666666666666666666666666666666666666666666666666666666666666666666666666666666666666666666666666666666666666666666666666666666666666666666666666666666666666666666666666666666666666666666666666666666666666666666666666666666666666666666666666666666666666666666666666666666666666666666666666666666666

0000000000000000000000000000000000000000000000000000000000000000000000000000111111111111111111111111111111111111111111111111111111111111111111111111111111111111111111111111111122222222222222222222222222222222222222222222222222222222222222222222222222222222222222222222222222223333333333333333333333333333333333333333333333333333333333333333333333333333333333333

2222223333333333444444444455555555556666666666777777777788888888889999999999000000000011111111112222222222333333333344444444445555555555666666666677777777778888888888999999999900000000001111111111222222222233333333334444444444555555555566666666667777777777888888888899999999990000000000111111111122222222223333333333444444444455555555556666666666777777777788888

4567890123456789012345678901234567890123456789012345678901234567890123456789012345678901234567890123456789012345678901234567890123456789012345678901234567890123456789012345678901234567890123456789012345678901234567890123456789012345678901234567890123456789012345678901234567890123456789012345678901234567890123456789012345678901234567890123456789012345678901234

CRS TTCTTTCATGGGGAAGCAGATTTGGGTACCACCCAAGTATTGACTCACCCATCAACAACCGCTATGTATTTCGTACATTACTGCCAGCCACCATGAATATTGTACGGTACCATAAATACTTGACCACCTGTAGTACATAAAAACCCAATCCACATCAAAACCCCCTCCCCATGCTTACAAGCAAGTACAGCAATCAACCCTCAACTATCACACATCAACTGCAACTCCAAAGCCACCCCTCACCCACTAGGATACCAACAAACCTACCCACCCTTAACAGTACATAGTACATAAAGCCATTTACCGTACATAGCACATTACAGTCAAATCCCTTCTCGTCCCCATGGATGACCCCCCTCAG

>T189

T189.1.1.1_rib ............................................................................................................ACCATAAATACTTGACCACCTGTAGTAC

T189.1.1.2_rib ............................................................................................................

T189.1.1.3_rib ............................................................................................................

T189.1.1.4_rib ............................................................................................................

T189.1.1.5_rib ............................................................................................................

T189.1.2.1_rib ............................................................................................................

T189.1.2.2_rib ............................................................................................................

T189.1.2.3_rib ............................................................................................................

T189.1.2.4_rib ............................................................................................................

T189.1.2.5_rib ............................................................................................................

T189.2.1.1_rib ............................................................................................................

T189.2.1.2_rib ............................................................................................................

T189.2.1.3_rib ............................................................................................................

T189.2.1.4_rib ............................................................................................................

T189.2.1.5_rib ............................................................................................................

T189.2.2.1_rib ............................................................................................................

T189.2.2.2_rib ............................................................................................................

T189.2.2.3_rib ............................................................................................................

T189.2.2.4_rib ............................................................................................................

T189.2.2.5_rib ............................................................................................................

T189.1.1.1_rib CGCTATGTATTTCGTACATTACTGC.........................................................................................................................................................CCTCACCCACTAGGATACCA

T189.1.1.2_rib .........................................................................................................................................................

T189.1.1.3_rib .........................................................................................................................................................

T189.1.1.4_rib .........................................................................................................................................................

T189.1.1.5_rib .........................................................................................................................................................

T189.1.2.1_rib .........................................................................................................................................................

T189.1.2.2_rib .........................................................................................................................................................

T189.1.2.3_rib .........................................................................................................................................................

T189.1.2.4_rib .........................................................................................................................................................

T189.1.2.5_rib .........................................................................................................................................................

T189.2.1.1_rib .........................................................................................................................................................

T189.2.1.2_rib .........................................................................................................................................................

T189.2.1.3_rib .........................................................................................................................................................

T189.2.1.4_rib .........................................................................................................................................................

T189.2.1.5_rib .........................................................................................................................................................

T189.2.2.1_rib .........................................................................................................................................................

T189.2.2.2_rib .........................................................................................................................................................

T189.2.2.3_rib .........................................................................................................................................................

T189.2.2.4_rib .........................................................................................................................................................

T189.2.2.5_rib .........................................................................................................................................................

T189.1.1.1_rib CAACTATCACACATCAACTGCAA.........................................................................................................................................

T189.1.1.2_rib .........................................................................................................................................

T189.1.1.3_rib .........................................................................................................................................

T189.1.1.4_rib .........................................................................................................................................

T189.1.1.5_rib .........................................................................................................................................

T189.1.2.1_rib .........................................................................................................................................

T189.1.2.2_rib .........................................................................................................................................

T189.1.2.3_rib .........................................................................................................................................

T189.1.2.4_rib .........................................................................................................................................

T189.1.2.5_rib .........................................................................................................................................

T189.2.1.1_rib .........................................................................................................................................

T189.2.1.2_rib .........................................................................................................................................

T189.2.1.3_rib .........................................................................................................................................

T189.2.1.4_rib .........................................................................................................................................

T189.2.1.5_rib .........................................................................................................................................

T189.2.2.1_rib .........................................................................................................................................

T189.2.2.2_rib .........................................................................................................................................

T189.2.2.3_rib .........................................................................................................................................

T189.2.2.4_rib .........................................................................................................................................

T189.2.2.5_rib .........................................................................................................................................

Consensus TTCTTTCATGGGGAAGCAGATTTGGGTACCACCCAAGTATTGACTCACCCATCAACAACCGCTATGTATTTCGTACATTACTGCCAGCCACCATGAATATTGTACGGTACCATAAATACTTGACCACCTGTAGTACATAAAAACCCAATCCACATCAAAACCCCCTCCCCATGCTTACAAGCAAGTACAGCAATCAACCCTCAACTATCACACATCAACTGCAACTCCAAAGCCACCCCTCACCCACTAGGATACCAACAAACCTACCCACCCTTAACAGTACATAGTACATAAAGCCATTTACCGTACATAGCACATTACAGTCAAATCCCTTCTCGTCCCCATGGATGACCCCCCTCAG

1111111111111111111111111111111111111111111111111111111111111111111111111111111111111111111111111111111111111111111111111111111111111111111111111111111111111111111111111111111111111111111111111111111111111111111111111111111111111111111111111111111111111111111111111111111111111111111111111111111111111111111111111111111111111111111111111111111111111111111111111

6666666666666666666666666666666666666666666666666666666666666666666666666666666666666666666666666666666666666666666666666666666666666666666666666666666666666666666666666666666666666666666666666666666666666666666666666666666666666666666666666666666666666666666666666666666666666666666666666666666666666666666666666666666666666666666666666666666666666666666666666

0000000000000000000000000000000000000000000000000000000000000000000000000000111111111111111111111111111111111111111111111111111111111111111111111111111111111111111111111111111122222222222222222222222222222222222222222222222222222222222222222222222222222222222222222222222222223333333333333333333333333333333333333333333333333333333333333333333333333333333333333

2222223333333333444444444455555555556666666666777777777788888888889999999999000000000011111111112222222222333333333344444444445555555555666666666677777777778888888888999999999900000000001111111111222222222233333333334444444444555555555566666666667777777777888888888899999999990000000000111111111122222222223333333333444444444455555555556666666666777777777788888

4567890123456789012345678901234567890123456789012345678901234567890123456789012345678901234567890123456789012345678901234567890123456789012345678901234567890123456789012345678901234567890123456789012345678901234567890123456789012345678901234567890123456789012345678901234567890123456789012345678901234567890123456789012345678901234567890123456789012345678901234

CRS TTCTTTCATGGGGAAGCAGATTTGGGTACCACCCAAGTATTGACTCACCCATCAACAACCGCTATGTATTTCGTACATTACTGCCAGCCACCATGAATATTGTACGGTACCATAAATACTTGACCACCTGTAGTACATAAAAACCCAATCCACATCAAAACCCCCTCCCCATGCTTACAAGCAAGTACAGCAATCAACCCTCAACTATCACACATCAACTGCAACTCCAAAGCCACCCCTCACCCACTAGGATACCAACAAACCTACCCACCCTTAACAGTACATAGTACATAAAGCCATTTACCGTACATAGCACATTACAGTCAAATCCCTTCTCGTCCCCATGGATGACCCCCCTCAG

>T189

T189.1.1.1_femur ............................................................................................................ACCATAAATACTTGACCACCTGTAGTAC

T189.1.1.2_femur ............................................................................................................

T189.1.1.3_femur ............................................................................................................

T189.1.1.4_femur ............................................................................................................

T189.1.1.5_femur ............................................................................................................

T189.1.2.1_femur ............................................................................................................

T189.1.2.2_femur ............................................................................................................

T189.1.2.3_femur ............................................................................................................

T189.1.2.4_femur ............................................................................................................

T189.1.2.5_femur ............................................................................................................

T189.2.1.1_femur ............................................................................................................

T189.2.1.2_femur ............................................................................................................

T189.2.1.3_femur ............................................................................................................

T189.2.1.4_femur ............................................................................................................

T189.2.1.5_femur ............................................................................................................

T189.2.2.1_femur ............................................................................................................

T189.2.2.2_femur ............................................................................................................

T189.2.2.3_femur ............................................................................................................

T189.2.2.4_femur ............................................................................................................

T189.2.2.5_femur ............................................................................................................

T189.1.1.1_femur CGCTATGTATTTCGTACATTACTGC.........................................................................................................................................................CCTCACCCACTAGGATACCA

T189.1.1.2_femur .........................................................................................................................................................

T189.1.1.3_femur .........................................................................................................................................................

T189.1.1.4_femur .........................................................................................................................................................

T189.1.1.5_femur .........................................................................................................................................................

T189.1.2.1_femur .........................................................................................................................................................

T189.1.2.2_femur .........................................................................................................................................................

T189.1.2.3_femur .........................................................................................................................................................

T189.1.2.4_femur .........................................................................................................................................................

T189.1.2.5_femur .........................................................................................................................................................

T189.2.1.1_femur .........................................................................................................................................................

T189.2.1.2_femur .........................................................................................................................................................

T189.2.1.3_femur .........................................................................................................................................................

T189.2.1.4_femur .........................................................................................................................................................

T189.2.1.5_femur .........................................................................................................................................................

T189.2.2.1_femur .........................................................................................................................................................

T189.2.2.2_femur .........................................................................................................................................................

T189.2.2.3_femur .........................................................................................................................................................

T189.2.2.4_femur .........................................................................................................................................................

T189.2.2.5_femur .........................................................................................................................................................

T189.1.1.1_femur CAACTATCACACATCAACTGCAA.........................................................................................................................................

T189.1.1.2_femur .........................................................................................................................................

T189.1.1.3_femur .........................................................................................................................................

T189.1.1.4_femur .........................................................................................................................................

T189.1.1.5_femur .........................................................................................................................................

T189.1.2.1_femur .........................................................................................................................................

T189.1.2.2_femur .........................................................................................................................................

T189.1.2.3_femur .........................................................................................................................................

T189.1.2.4_femur .........................................................................................................................................

T189.1.2.5_femur .........................................................................................................................................

T189.2.1.1_femur .........................................................................................................................................

T189.2.1.2_femur .........................................................................................................................................

T189.2.1.3_femur .........................................................................................................................................

T189.2.1.4_femur .........................................................................................................................................

T189.2.1.5_femur .........................................................................................................................................

T189.2.2.1_femur .........................................................................................................................................

T189.2.2.2_femur .........................................................................................................................................

T189.2.2.3_femur .........................................................................................................................................

T189.2.2.4_femur .........................................................................................................................................

T189.2.2.5_femur .........................................................................................................................................

Consensus TTCTTTCATGGGGAAGCAGATTTGGGTACCACCCAAGTATTGACTCACCCATCAACAACCGCTATGTATTTCGTACATTACTGCCAGCCACCATGAATATTGTACGGTACCATAAATACTTGACCACCTGTAGTACATAAAAACCCAATCCACATCAAAACCCCCTCCCCATGCTTACAAGCAAGTACAGCAATCAACCCTCAACTATCACACATCAACTGCAACTCCAAAGCCACCCCTCACCCACTAGGATACCAACAAACCTACCCACCCTTAACAGTACATAGTACATAAAGCCATTTACCGTACATAGCACATTACAGTCAAATCCCTTCTCGTCCCCATGGATGACCCCCCTCAG
